# Enzyme discovery beyond homology: a unique hydroxynitrile lyase in the Bet v1 superfamily

**DOI:** 10.1038/srep46738

**Published:** 2017-05-03

**Authors:** Elisa Lanfranchi, Tea Pavkov-Keller, Eva-Maria Koehler, Matthias Diepold, Kerstin Steiner, Barbara Darnhofer, Jürgen Hartler, Tom Van Den Bergh, Henk-Jan Joosten, Mandana Gruber-Khadjawi, Gerhard G. Thallinger, Ruth Birner-Gruenberger, Karl Gruber, Margit Winkler, Anton Glieder

**Affiliations:** 1 Austrian Centre of Industrial Biotechnology, Petersgasse 14, 8010 Graz, Austria; 2 University of Graz, Institute of Molecular Biosciences, NAWI Graz, Humboldtstrasse 50, 8010 Graz, Austria; 3 Medical University of Graz, Institute of Pathology, Stiftingtalstrasse 24, 8010 Graz, Austria; 4 Omics Center Graz, BioTechMed Graz, Stiftingtalstraße 24, 8010 Graz, Austria; 5 Graz University of Technology, Institute of Molecular Biotechnology, NAWI Graz, Petersgasse 14, 8010 Graz, Austria; 6 Bio-prodict BV, Nieuwe Marktstraat, 54E 6511 AA, Nijmengen, The Netherlands

## Abstract

Homology and similarity based approaches are most widely used for the identification of new enzymes for biocatalysis. However, they are not suitable to find truly novel scaffolds with a desired function and this averts options and diversity. Hydroxynitrile lyases (HNLs) are an example of non-homologous isofunctional enzymes for the synthesis of chiral cyanohydrins. Due to their convergent evolution, finding new representatives is challenging. Here we show the discovery of unique HNL enzymes from the fern *Davallia tyermannii* by coalescence of transcriptomics, proteomics and enzymatic screening. It is the first protein with a Bet v1-like protein fold exhibiting HNL activity, and has a new catalytic center, as shown by protein crystallography. Biochemical properties of *D. tyermannii* HNLs open perspectives for the development of a complementary class of biocatalysts for the stereoselective synthesis of cyanohydrins. This work shows that systematic integration of -omics data facilitates discovery of enzymes with unpredictable sequences and helps to extend our knowledge about enzyme diversity.

Hydroxynitrile lyases (HNLs) catalyze the cleavage of cyanohydrins into the corresponding aldehyde or ketone and hydrogen cyanide^1^,^2^,^3^. HNLs have been mostly found in plants, but examples exist from bacteria^4^,^5^ and arthropods^6^ as well. HNLs from the three kingdoms show no homology, and in plants three non-homologous HNL families are currently known. HNLs are involved in the cyanogenic pathway, a widespread defense mechanism against herbivores and pathogens. Plants produce cyanogenic glycosides *e.g.* prunasin or amygdalin, store these compounds in different tissues, and enzymatically degrade them upon tissue disruption, in order to finally release toxic hydrogen cyanide^2^. Similarly, arthropods produce HCN from mandelonitrile as a chemical for their defense^6^. The role of HNLs in bacteria is not yet explored.

HNLs are well-established tools for selected biocatalytic industrial processes. They can perform the reverse condensation reaction (carbon-carbon bond formation), and synthesize α-cyanohydrins in a stereospecific manner. Cyanohydrins are building blocks for a number of follow-up reactions, and the products find application in agrochemical, pharmaceutical and cosmetic industries^7^,^8^,^9^,^10^. Although (*R*)- and (*S*)-selective HNLs are exploited in biocatalysis, new enzymes with outstanding characteristics are constantly sought for. Limitations of currently known HNLs are low activity^11^, low stability^12^, low expression levels^6^, or low flexibility in the choice of the expression host^13^. Approximately 3,000 plant species utilize cyanogenic defense, and HNL activity has been shown in a wide variety of families^14^,^15^,^16^,^17^, but only few amino acid sequences of confirmed HNLs are found in common gene and protein databases. However, knowledge of an enzyme’s amino acid sequence is essential for its recombinant expression and engineering. This – in turn – is prerequisite for applications on scale.

The determination of truly new sequences is the weak point for this enzyme class and the reason is related to protein evolution. HNLs are an example of non-homologous isofunctional enzymes (NISE), a group of unrelated proteins that catalyze the same chemical reaction as a result of convergent evolution^18^. Therefore, the typical homology based discovery approaches are unsuitable to elucidate novel HNL sequences. To date, five different protein folds have been associated to HNL activity and they do not share any conserved motif. Crystal structures of HNLs belonging to cupin^11^, GMC oxidoreductase^19^, α/β-hydrolase^20^,^21^,^22^ and peptidase S10^23^ families have been determined. Moreover, by sequence homology, an HNL has been described as a member of the zinc-binding dehydrogenase family^24^. The first HNL sequence from invasive millipedes was discovered by a five step purification of the protein from kilogram quantities of millipede animals and subsequent Edman degradation for elucidation of the amino acid sequence. Its sequence indicates that it does not belong to any of the above mentioned protein folds^6^. Finally there is a number of characterized HNLs with yet unpublished amino acid sequences and protein folds, for example, *Pat*HNL (*Prunus amygdalus turcomanica*)^25^, *Pars*HNL (*Prunus armeniaca* L.)^26^, *Pe*HNL (*Passiflora edulis*)^27^ (sequence information for PeHNL is public since December 2016, see Ref 28), and the fern HNL from *Phlebodium aureum (Pha*HNL)^28^. For the latter enzyme, outstanding specific activity was reported, and also indications that it is distinct to known HNLs^29^. Biochemical characterizations and experiments towards cyanohydrin synthesis were performed with proteins isolated from the natural sources.

A classical HNL identification workflow would comprise of several steps of protein purification starting from large amounts of the natural source to obtain highly pure enzyme. Sequence information is then typically obtained by Edman degradation or tryptic digestion and mass spectrometry. The full sequence can then be identified by PCR using degenerate primers. Altogether, this is a very laborious and time consuming process. Here, we describe an alternative approach for the discovery of a novel and unique HNL from the white rabbit’s foot fern *Davallia tyermannii* (alternative names: *Humata tyermannii*; *Humata tyermannii* T. Moore; *Davallia tyermannii* (T. Moore) Backer) by the combination of different -*omics* data and enzymatic screening. To our knowledge, it is the first time where -*omics* techniques have been combined for the determination of an HNL sequence from scratch. In addition to enzyme characterization and synthesis of cyanohydrins, we determined the tertiary structure of *Davallia tyermannii* HNL (*Dt*HNL) and proposed its catalytic mechanism. Finally, we investigated the presence of HNLs in different fern families.

## Results

### From the enzymatic activity to the sequence

Based on the first report of a highly active HNL in the fern *Phlebodium aureum*^29^, we first screened a selection of ferns and identified cyanogenic activity of *Davallia tyermannii* leaves by olfactory screening of disrupted plant tissue. Cyanogenic phenotype does not always correlate with the presence of a hydroxynitrile lyase (HNL). Therefore, HNL activity was confirmed in the cyanogenic fern *Davallia tyermannii* by the addition of (*RS*)-2-hydroxy-2-phenylacetonitrile (racemic mandelonitrile) to a protein preparation as described in the Methods section and subsequent detection of the release of hydrogen cyanide^30^ (Supplementary Result 1). Mandelonitrile was chosen, as this is the natural cyanohydrin identified in the genus *Davallia* and different other fern genera^28^,^31^,^32^.

The sequence discovery workflow combined several techniques. We collected the information about all expressed genes, which is well described by a sequenced normalized transcriptome. For this purpose, high quality mRNA from *D. tyermannii* leaves and croziers was isolated and the normalized cDNA library was sequenced.

**Figure 1:**
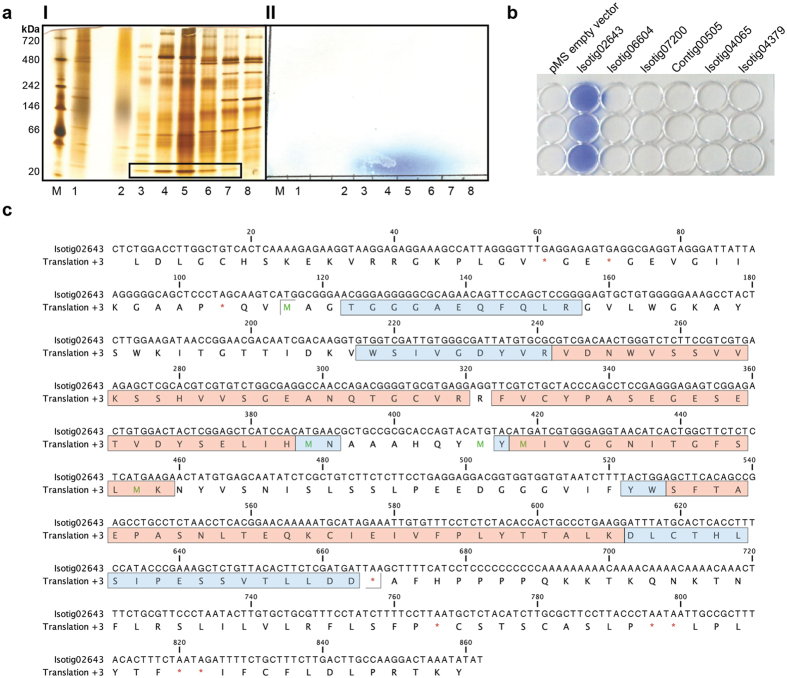
Identification of the *Dt*HNL sequence. A: Blue Native PAGE followed by HNL activity assay. (**a**) I: Concentrated fractions of anion exchange purification were applied separately on BN PAGE. NativeMark^™^ Unstained Protein Standard (Thermo Fisher Scientific); total protein extract from *D. tyermannii* leaves (1); flow through (2); elution fractions (3–8); active bands at 20 kDa are highlighted in the box. (**a**) II: HNL activity is depicted by the blurred blue spot corresponding to purification fractions 2–7. The total protein extract shows a weak signal (1). Assay conditions: 100 mM citrate buffer pH 4.0; substrate: racemic mandelonitrile. Incubation time 8 min. (**b**) Screening of putative HNL sequences. Cell free lysate of *E. coli* TOP 10 F’ strains expressing six putative HNL proteins. Each sample was tested in triplicate: 30 μL (1), 20 μL (2), 10 μL (3) of cell free lysate, respectively. *E. coli* TOP 10F’ transformed with pMS vector was used as negative control. Assay conditions: 100 mM citrate buffer pH 4.5; substrate: racemic mandelonitrile 13 mM. The intensive blue spots developed after few seconds of incubation. Proteins with unknown function were named as the relative isotig or contig number found in the transcriptome. (**c**) Nucleotide and amino acid sequence of isotig 02643 (*Dt*HNL1). Fragments detected by mass spectrometry are labeled. Indicated results were obtained from the protein bands excised from lanes 4 (red) and 5 (red and blue). The peptides identified by mass spectrometry cover 72% of the open reading frame.

Transcriptome data is sufficient for enzyme discovery, only when information about the primary sequence, conserved residues, domains or motifs is available. As homology based approaches produced no hits in the transcriptome, retrieval of information on the protein level was necessary. To pinpoint HNL fragments, we subjected proteins from active tissues to anion exchange chromatography and subsequently to an BN PAGE coupled HCN detection assay^33^. Several elution fractions showed enzymatic activity, which correlated to blue spots at approximately 20 kDa bands (Fig. 1a). These were excised and the protein mixture was subjected to tryptic digestion and LC-MS/MS analysis. LC-MS/MS peptide data were matched by searching the translated nucleotide transcriptome database from *D. tyermannii*. Thirty-six identified protein sequences were further ranked by an exclusion process based on predicted protein size, signal peptide and similarity with known protein sequences (Supplementary Dataset 1). The sequence with the highest probability to represent the protein with HNL activity was isotig02643. Nevertheless, the top six candidates were recombinantly expressed in *E. coli* and tested for HNL activity (Fig. 1b; Supplementary Table 3).

The protein encoded by the open reading frame (ORF) of isotig02643 showed HNL activity, when racemic mandelonitrile was added to cell free lysate from *E. coli* (Fig. 1b). The other five candidate proteins did not show HNL activity and were not investigated further. The peptides retrieved from mass spectrometry cover 72% of the translated ORF of isotig02643 (Fig. 1c). Translated nucleotide transcriptome database revealed three additional highly similar sequences to the confirmed HNL: translated ORFs of isotig02641, isotig07602 and contig00751 show at least 93.5% identity with isotig02643 (Supplementary Fig. 5a). The respective three genes were amplified from *D. tyermannii* gDNA by PCR, and their nucleotide sequences were confirmed by Sanger sequencing (Supplementary Table 4). After recombinant expression in *E. coli*, the HNL activity assay confirmed cyanogenic activity of all three isoenzymes (Supplementary Fig. 5b). The four proteins coded by the ORF of isotig02643, 02641, 07602 and contig00751 were named *Dt*HNL1, *Dt*HNL2, *Dt*HNL3 and *Dt*HNL4, respectively.

The novelty of *Dt*HNL was investigated by BLAST^34^ of known HNLs against the obtained transcriptome. Low identity and sequence coverage in the output confirmed that *Dt*HNL is distinct from known HNL classes (Supplementary Results 3).

### Characterization of *Dt*HNL isoenzymes

**Figure 2:**
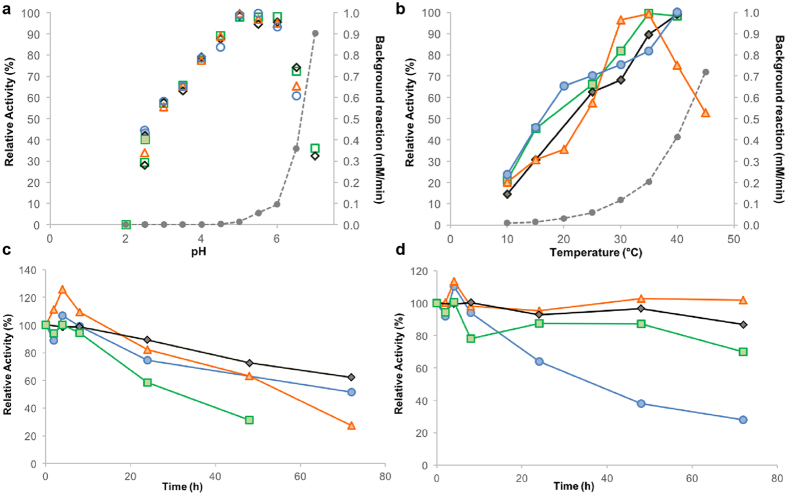
Biochemical properties of *Dt*HNL isoenzymes. *Dt*HNL1 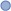; *Dt*HNL2 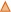; *Dt*HNL3 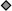; *Dt*HNL4 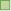. Grey dashed lines indicate the spontaneous degradation of racemic mandelonitrile in a negative control reaction without enzyme addition (background reaction). Standard enzymatic assay was performed by monitoring benzaldehyde formation at 280 nm. Values were obtained from the average of a minimal of two and a maximum of three independent samples, each of which is the average of two or three technical replicates. Standard deviations are within the 20% threshold (or 25% for temperature profile). For clarity, error bars have been omitted. (**a**) pH profile. Relative activity of *Dt*HNL isoenzymes at different pH values from 2.0 to 7.0. The assay was performed in HCl-potassium chloride buffer (filled symbols), or sodium citrate-phosphate buffer (empty symbols). Activity of *Dt*HNL1 and 2 at pH 7.0 is not depicted due to high standard deviations. (**b**) Temperature profile. Relative activity of *Dt*HNL isoenzymes at different temperatures from 10 to 50 °C. The assay was performed at pH 5.0. Omitted points are due to high standard deviations. Enzyme stability at pH 2.5 (**c**) and at pH 4.0 (**d**). Activity after incubation of *Dt*HNL isoenzymes at pH 2.5 or 4.0, respectively, and 8 °C. Relative activity is based on the activity before incubation.

Biochemical features of all four isoenzymes were determined after heterologous expression and purification of the His-tagged proteins by affinity chromatography (Supplementary Results 6). The influence of pH and temperature on the catalytic activity was investigated by following the cleavage reaction of racemic mandelonitrile. Maximum activity was observed at pH 5.0. The activity linearly decreased in more acidic environment and was zero at pH 2.0. Remarkably, all isoforms were active at pH 2.5, and retained about 80% of their activity at pH 4.0 (Fig. 2a). Enzymatic activity at low pH is an essential asset for HNLs in terms of their application, since cyanohydrins easily degrade at pH ≥ 5.0 as indicated by the background reaction line (Fig. 2a). Enzymatic syntheses of cyanohydrins are preferably performed at pH 4.0 or lower and robust biocatalysts in acidic environment are highly desired. Therefore, we investigated *Dt*HNL stability at pH 2.5 and 4.0 by incubation at 8 °C for 72 hours. Residual activity was more than 50% at both pH 2.5 and pH 4.0. (Fig. 2c,d) after 24 hours. Isoform 1 appeared to be less stable at pH 4.0 than *Dt*HNL2, 3 and 4 (Fig. 2d). Additionally, enzymatic stability was tested at pH 5.0, and residual activity was more than 65% at 72 hours of incubation (Supplementary Table 5).

Figure 2b shows *Dt*HNL activity at different temperatures. The optimum was observed at 35 °C for *Dt*HNL2 and 4 and 40 °C for *Dt*HNL1 and 3, respectively. Mandelonitrile spontaneously degrades above pH 5.5 and elevated temperature also contributes to degradation, resulting in increased apparent reaction rates, as indicated by the background reaction curve (Fig. 2a,b). pH 5.0 and 25 °C were used as a good compromise between optimal enzymatic activity and repressed substrate decomposition.

Finally, the Michaelis constants *K*_m_ and turnover numbers *k*_cat_ were determined on basis of the Michaelis-Menten model for the cleavage of (*R)*-mandelonitrile. All *Dt*HNLs have high affinity for (*R*)-mandelonitrile as indicated by *K*_m_ values of 0.30 ± 0.03 mM *Dt*HNL1, 0.45 ± 0.05 mM *Dt*HNL2, 0.75 ± 0.08 mM *Dt*HNL3 and 0.63 ± 0.06 mM *Dt*HNL4, respectively. These results are consistent with *K*_m_ values of reported (*R*)-HNL enzymes^2^. The turnover numbers were 144 ± 2 s^−1^ for *Dt*HNL1, 156 ± 3 s^−1^ for *Dt*HNL2, 356 ± 8 s^−1^ for *Dt*HNL3 and 272 ± 5 s^−1^ for *Dt*HNL4, respectively.

Kinetic parameters for *Dt*HNL1 mediated synthesis of (*R*)-mandelonitrile were determined in a biphasic system (1:2 aqueous:organic phase) at pH 4.0 and 10 °C. Hydrocyanic acid was kept in saturating concentration and the initial rate was measured at different concentrations of benzaldehyde. Kinetic parameters were calculated using the Michaelis-Menten model as described for the reaction in the cleavage direction. The majority of (*R*)-mandelonitrile is in the organic layer and the minor amount in the aqueous buffer was omitted for the quantification. Under these conditions, *v*_max_ of *Dt*HNL1 was 377 ± 47 μmol min^−1^ mg^−1^, and *K*_m_ for benzaldehyde was 14 ± 2 mM. *k*_cat_ was 70 ± 7 s^−1^ and the enzymatic efficiency *k*_cat_/*K*_m_ was 5 ± 1 s^−1^ mM^−1^, assuming *Dt*HNL1 activity in the aqueous phase only.

### *Dt*HNL structure and reaction mechanism

**Figure 3:**
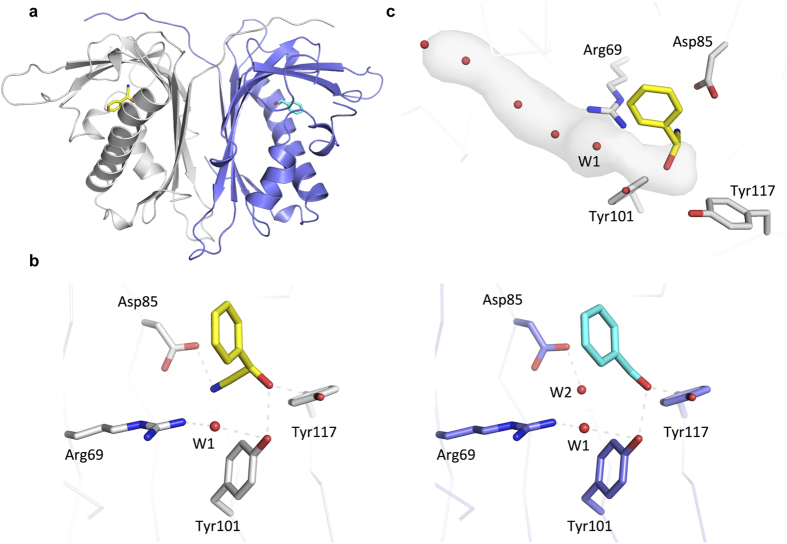
Crystal structure *Dt*HNL1. (**a**) Overall structure of the *Dt*HNL1 dimer. Individual protomers are shown in a grey or blue ribbon representation. Bound ligands are shown in a stick representation (mandelonitrile in yellow and benzaldehyde in cyan). (**b**) Close-up view of the active site. Residues potentially important for the enzymatic activity are shown in a sticks representation and water molecules are depicted as red spheres. The bridging water is labeled W1. Dashed lines indicate plausible hydrogen bonding interactions. Bound benzaldehyde (cyan, right) and (*R*)-mandelonitrile (yellow, left) as observed in a crystal soaked with (*R*)-mandelonitrile are drawn in a sticks representation. (**c**) Access to the active site. Water channel extending from the active site to the surface of the protein as calculated using the program CAVER^49^.

We determined the crystal structure of *Dt*HNL using selenomethionine single-wavelength-anomalous-dispersion (SeMet-SAD) to a resolution of 1.85 Å. The enzyme is a dimer and exhibits a Bet v1-like fold. The Bet v1 superfamily is composed of sequences related to the major Birch (*Betula verrucose*) pollen allergen Bet v1. The fold is composed of an anti-parallel β-sheet, which is wrapped around a long C-terminal α-helix (Fig. 3a). The ligand binding cavity is situated between the β-sheet and the helix. In proteins of the Bet v1 superfamily, this cavity plays important roles in the binding and metabolism of large, hydrophobic compounds such as lipids, hormones, and antibiotics^35^. We also determined the structures of complexes of *Dt*HNL with 4-hydroxy benzaldehyde, benzoic acid and (*R*)-mandelonitrile/benzaldehyde using soaking techniques (Supplementary Table 6). Clear electron density was observed for those ligands bound in the canonical binding cleft of the Bet v1 fold (Supplementary Fig. 7a).

In all three structures, the aromatic moiety of the different ligands is bound in a hydrophobic pocket formed by the side chains of Val44, Val48, Trp47, Val51, Val52, Phe71, Cys73, Ile108, Phe111, Trp138, Leu160 and Ala164. Especially the valine residues at positions 44, 48, 51 and 52 together with Phe71 and Leu160 seem to be important to shape the cavity. A part of the phenyl ring is also pointing towards the solvent, therefore, larger substrate may be bound in a way that substituents are located in the active site access tunnel.

The polar parts of the ligands (the OH-, carbonyl- or carboxyl-group) are hydrogen bonded to Tyr101 and Tyr117 (Fig. 3b and Supplementary Fig. 7b). In the complex with (*R*)-mandelonitrile, the cyano group interacts with the guanidinium group of Arg69 (distance 3.5 Å) and the carboxylic acid group of Asp85 (2.9 Å). The latter interaction requires Asp85 to be protonated in this complex structure. In the other complexes, a water molecule occupies this position. The active site cavity is clearly asymmetric which provides a reasonable, qualitative explanation for the stereospecificity of *Dt*HNL.

**Figure 4:**
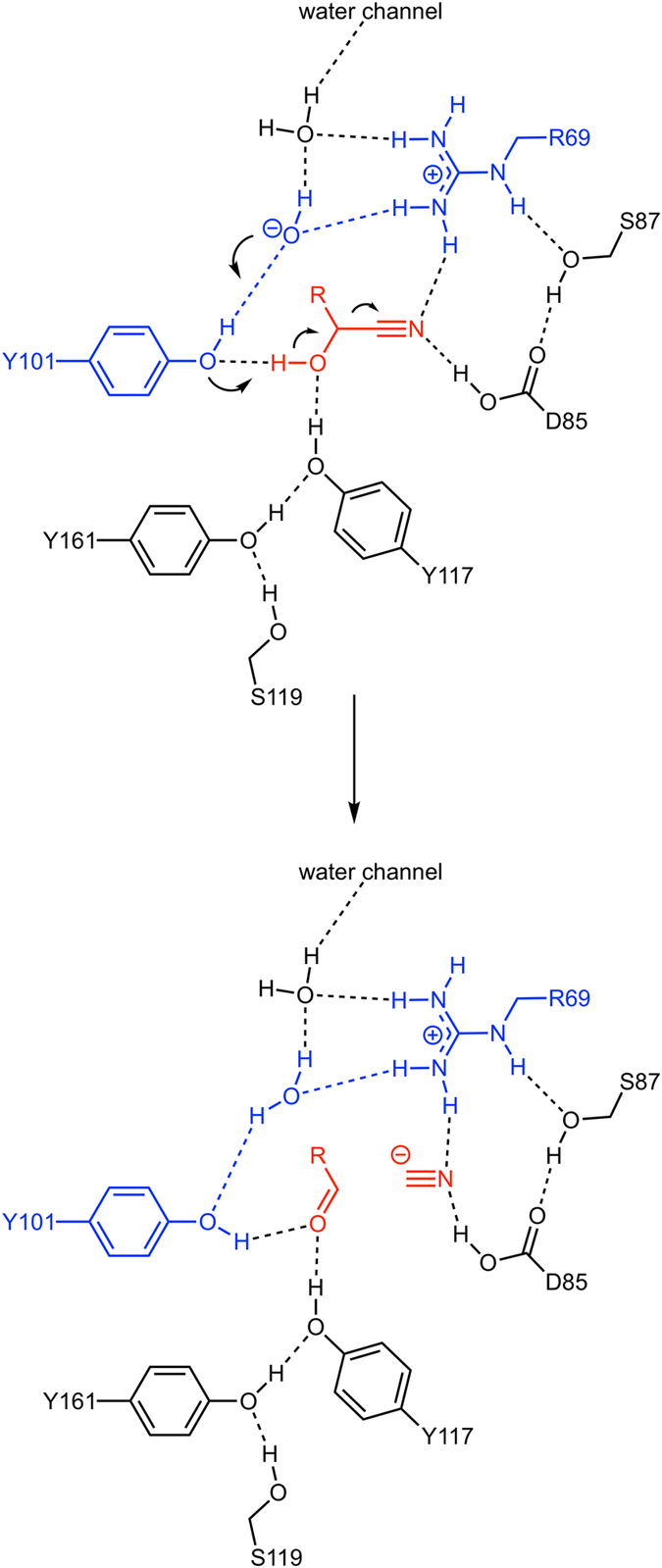
Proposed catalytic mechanism for the *Dt*HNL1. Cyanohydrin cleavage of (*R*)-mandelonitrile based on complex-crystal structures and mutagenesis experiments.

The observed polar interactions between (*R*)-mandelonitrile and *Dt*HNL suggest that the OH-group of the substrate is deprotonated by Tyr101, which is facilitated by the additional hydrogen bond from Tyr117. A tyrosine residue alone, however, is not a typical base, especially at lower pH-values (pK_a_ of tyrosine ~10) and there is no other amino acid residue in vicinity (such as a histidine), which could activate the phenol. A water molecule bridges the OH-group of Tyr101 and the guanidinium group of Arg69 (Fig. 3b). There are several possibilities, how two protons can be distributed between these groups: a) Tyr-OH, OH^−^, Arg^+^, b) Tyr-OH, H_2_O, Arg^0^ and c) Tyr-O^−^, H_2_O, Arg^+^. Based on the x-ray crystal structures, it is not possible to decide which of the three configurations is the correct one, although a positively charged arginine residue appears to be more plausible (Fig. 4). After cyanohydrin cleavage only one configuration (Tyr-OH, H_2_O, Arg^+^) is consistent with the structural data. The negative charge emerging at the cyano group upon C-C bond cleavage is very likely stabilized by the positive charge of Arg69 and a hydrogen bond from the protonated Asp85.

The bridging water is the first of five water molecules, which are nicely aligned in a channel that runs from the active site to the surface of the protein (Fig. 3c). This channel is approximately orthogonal to the main entrance tunnel to the active site and could serve as an access/exit pathway for HCN or as a proton relay to the bulk solvent.

Based on this mechanistic proposal, a number of amino acids were replaced and the enzymatic activity of these variants was determined. Exchange of Tyr101 by phenylalanine led to a complete loss of activity. The replacement of Asp85 and Ser87 by alanine or of Tyr117 and Tyr161 to phenylalanine decreased the activity by at least 90%. Other amino acid exchanges (especially of Arg69) produced insoluble protein and prevented activity measurements (Supplementary Table 7).

### Different HNL classes from ferns

Ferns are a group of sparsely studied vascular plants, which include several classes and consequently thousands of species. Ferns can be considered as an outgroup of the plant kingdom as they maintained the ancestral condition and show different characteristics compared to seed plants. For example, typically they have much higher chromosome numbers and larger genomes^36^. The sequence of *Dt*HNL is the first HNL sequence identified from ferns, although several fern species adopt cyanogenesis as chemical defense strategy. We aimed to investigate whether cyanogenic ferns express homologous HNLs or developed them independently, as often happens in seed plants.

**Figure 5:**
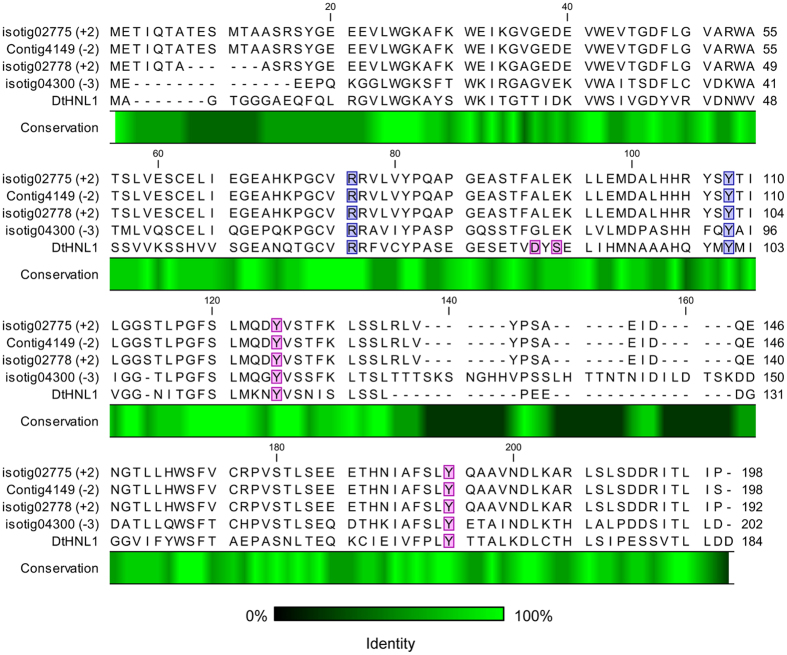
Multiple sequence alignment. Isotig02775 and isotig02778 are proteins with similarity to *Dt*HNL1 from the transcriptome of *P. aquilinum* leaves. Contig4149 was found in the transcriptome obtained from a *P. aquilinum* gametophyte^36^. Isotig04300 is the sequence most similar to isotig02775 and isotig02778, which was found in *D. tyermannii.* Isotig translation frame is indicated in brackets. Conservation % is indicated by a color code. Residues involved in substrate binding and catalysis are highlighted. Alignment was built with CLC Main Workbench 7.6.2 (QIAGEN). Proteins with unknown function were named as the relative isotig or contig number found in the transcriptome.

The braken fern *Pteridium aquilinum* (L.) Kuhn is widespread throughout the northern hemisphere and Africa and belongs to the *Dennstaedtiaceae* family^36^. It is another well-known example of cyanogenic fern in addition to *D. tyermannii* and *P. aureum,* and was easily accessible for our studies from an Austrian forest. First, high quality mRNA was isolated from enzymatically active leaves and croziers. The transcriptome was obtained by sequencing the normalized cDNA library (the quality of the transcriptome and assembly results are reported in Supplementary Results 2). The transcriptome did not show proteins with significant similarity to any known HNL sequences upon TBLASTN search (Supplementary Results 3). When *Dt*HNL1 was used as query for TBLASTN search in the *P. aquilinum* transcriptome database, we obtained 17 hits (Supplementary Table 8). Two sequences with identity to *Dt*HNL1 above 35%: isotig02775 and isotig02778 were chosen. They belong to the same isogroup and their ORFs encode for two 96% identical proteins (Supplementary Table 8). The identity to *Dt*HNL1 is 41% and the catalytic residues Arg69 and Tyr101 as well as Tyr117 and Tyr161 in the active site are conserved (Fig. 5). *Dt*HNL1 was subjected to a second TBLASTN search in a published translated transcriptome from *P. aquilinum* obtained during its gametophyte life stage^36^. The protein coded by the ORF of contig4149 is 99% identical to isotig02775, corroborating our hit (Fig. 5). However, when we subjected the three amino acid sequences obtained by the translation of isotig02275, isotig02778 and contig4149 to a TBLASTN search in the *D. tyermannii* transcriptome database, isotig04300 was obtained as the best hit instead of expected *Dt*HNL1 or its isoenzymes.

Isotig04300 codes for a protein which belongs to the Bet v1 protein superfamily and it is 39% identical to *Dt*HNL1 (55% similarity). However, it was not identified by mass spectrometry in fractions of *D. tyermannii* with HNL activity (Supplementary Dataset 1). This led to the hypothesis that similar proteins from *P. aquilinum* (Isotig02775, isotig02778 and contig4149) do not catalyze cyanohydrin cleavage and formation. To test this assumption, isotig02775 was expressed as soluble protein in *E. coli* and purified by affinity chromatography. The cyanogenic activity of isotig02775 was zero for racemic mandelonitrile, confirming our hypothesis. The protein was also inactive after mutation of Ala92 for Ser (the position corresponding to Asp85 in *Dt*HNL1). A double mutation of isotig02775 Ala92Asp and Glu94Ser, simulating *Dt*HNL1 at this site, lead to quantitative inclusion body formation, preventing the determination of the mutant’s activity. The substitution of Ala92 into aspartate seems to be a destabilizing mutation. This might be due to its strong polar character and bulkier structure of the side chain of aspartate.

### *Dt*HNL catalyzes the synthesis of cyanohydrins

To explore the potential of *Dt*HNL, we studied activity and stereoselectivity for the synthesis of cyanohydrins. In HNL-catalyzed reactions, high yields of enantiomerically pure product are compromised by spontaneous non-enzymatic formation of racemic cyanohydrin and racemization of the product due to equilibration of the reaction. Therefore, it is particularly important to suppress the chemical condensation and racemization of cyanohydrins and opt for conditions which allow the enzyme to outperform the non-enzymatic transformations. The decrease of the water content by the use of a biphasic systems and low pH are two solutions extensively reported in literature^37^. The choice of pH and the organic solvent/water phase ratio partly depend on the activity and stability of the biocatalyst. Here, we chose pH 4.0 and a 2:1 ratio v/v organic phase/aqueous phase. Water is necessary for the activity, as described above. *Dt*HNL was sufficiently stable at pH 4.0 for 24 hours (Fig. 2d) and showed ca. 80% of its maximum activity at this pH (Fig. 2a). We used 3 mg of *Dt*HNL1 (equal to 0.026 mol% for aldehyde substrates or 0.043 mol% for the ketone substrate) and monitored the cyanohydrin synthesis as summarized in Table 1.

*Dt*HNL1 accepted different aldehyde types, and at least one ketone (1-phenylethanone). It showed high selectivity for the (*R*)-products and high reaction rates. The maximum conversion and ≥99% enantiomeric excess were obtained in less than 2 hours for the synthesis of (*R*)-mandelonitrile and (*2 S*)-furan-2-yl-hydroxyacetonitrile (Table 1, entries 1 and 5). As expected for a natural non-engineered enzyme, benzaldehyde was the best substrate, and yield and enantiomeric purity of the product were not affected by decreasing the catalyst loading. We obtained 97.6% conversion and ≥ 99% enantiomeric excess (*ee*) in 24 hours, using more than hundred times less catalyst (0.2 e^−3^ mol% of *Dt*HNL1). Depending on the substrate structure, the enzyme competes more or less with the chemical condensation reaction for substrate availability, as indicated by the control values in Table 1. Low values for 1-phenylethanone and 3-phenylprop-2-enal reflect little or no background reaction (Table 1, entries 4 and 6).

Low enantiomeric excess was obtained for (*2R*)-2-hydroxy-4-phenylbutanenitrile (Table 1, entry 3) as a consequence of slow enzymatic transformation combined with fast unselective background. Consistent results were observed with less biocatalyst loading: 90.4% conversion, 43.5% *ee* and 97.0% conversion, 50.4% *ee* were retrieved with 1.7 e^−3^ mol% and 8.7 e^−3^ mol% of *Dt*HNL1 respectively, after 24 hours. These results indicate that *Dt*HNL1 wild-type displays intrinsically low stereoselectivity for 3-phenylpropanal cyanohydrin formation, due to flexibility of the propanal moiety in the active site and possibly two binding modes. The decrease of *ee* over time for (*R*)-mandelonitrile, (*2R*)-2-(2-chlorophenyl)-2-hydroxyacetonitrile, (*2R*)-2-hydroxy-4-phenylbut-3-enenitrile and (*2S*)-furan-2-yl-hydroxyacetonitrile (Table 1, entries 1,2,4,5) is attributed to the cyanohydrin racemization in buffer^38^. Finally, *Dt*HNL1 can convert aromatic ketones such as 1-phenylethanone, however, in low analytical yield (<24%), which reveals ketones as more difficult substrates for this enzyme (Table 1, entry 6). Lower activities for ketones as substrates in comparison to aldehydes were also observed for α/β-hydrolase fold HNLs^39^ and GMC oxidoreductase fold HNLs^40^. The cupin fold HNL from *Granulicella tundricola*, e.g. does not catalyze the addition of cyanide to ketones^11^.

## Discussion

Several bioinformatic tools have been developed for enzyme discovery, especially for wide screening of metagenomic libraries and sequenced genomes of bacteria and fungi. These tools are mainly based on similarities between sequences or common features such as characteristic protein motifs. Therefore, only homologous genes can be identified, even if they are distantly related.

Species varieties evolved different solutions to address the same issue. For example, alkane hydroxylations are catalyzed by completely dissimilar enzymes such as methane monooxygenase^41^, cytochrome P450s (CYP153A6^42^ or CYP52 from *Candida*^43^, alkB from *Pseudomonas*^44^), or fungal peroxygenases^45^, indicating that convergent evolution is well represented in nature for important biochemical reactions. Plants adopt several defense tactics against herbivory, chemical or mechanical attack. This is also valid at the molecular level, which is exemplified by hydroxynitrile lyases. Nature developed numerous strategies to catalyze the same chemical reaction – cyanogenesis - and the only common feature between the different classes is acid/base catalysis^46^.

We discovered a protein with hydroxynitrile forming activity unique within the Bet v1 superfamily. The most similar characterized protein is the lachrymatory factor synthase from onion where the sequence identity is less than 25%. Other family members are polyketide cyclases2 and abscisic acid receptor; most other sequences are annotated as unknown proteins. Additional examples of sequences with described function in the superfamily are norcoclaurine synthases^47^ or proteins with *in vitro* RNAase activity^35^. However, they are significantly distant from *Dt*HNL and they were classified differently in Pfam (pf00407, *Dt*HNL pf10604).

Based on structural analyses and mutation studies, we identified six residues responsible for the substrate binding and catalysis. Specifically, Arg69 and Tyr101 are directly involved in the catalysis together with a water molecule. Tyr117, Asp85, Ser87 and Tyr161 are also relevant for enzymatic activity. The catalytic residues, Tyr117 and Tyr161 are conserved in isotig02775, however, they are not sufficient for the activity. Aspartate in position 85 and serine in position 87 seem to be most critical (Isotig02775 numbering: 92 and 94) to confer HNL activity. These residues are occupied by alanine and glutamic acid in isotig02275 and the respective protein was inactive. Engineering the two critical residues (Ala92Ser and Ala92Asp-Glu94Ser) resulted in inactive mutants. Interestingly, *Dt*HNL is the only sequence in the entire Bet v1 superfamily which exhibits the six residues simultaneously. A glutamic acid in position 87 (*Dt*HNL numbering) is strongly conserved in a protein subset created with 3DM^48^, where Arg69, Tyr101 and Tyr117 were fixed (Supplementary Fig. 10g), and this residue is likely not compatible with HNL activity in a Bet v1 fold protein. It appears to be unlikely to identify another protein in the Bet v1 protein superfamily with HNL activity, based on today’s knowledge (Supplementary Results 10).

*P. aquilinum* expresses an HNL (*Pta*HNL) with similar protein size, however, this enzyme appears to belong to yet another protein family, as confirmed by the list of putative HNL sequences obtained by our approach (Supplementary Dataset 2).

From the biocatalytic point of view, we discovered promising enzymes for cyanohydrin synthesis. Recombinant *Dt*HNL isoenzymes display excellent specific activities and are tolerant to low pH conditions, the optimal environment for cyanohydrins. *Dt*HNL1 efficiently converts different aldehydes into the respective cyanohydrins, allowing for short reaction times. Excellent *ee* can be achieved by keeping reaction times short and adjusting the biocatalyst amount. Enantiomerically pure products can be obtained with good yields already with the wild type enzyme for entries 1, 2, 4 and 5 (Table 1), whereas engineering of the protein would be necessary for improving the stereoselectivity of *Dt*HNL1 for (*R*)-3-phenylpropionaldehyde cyanohydrin production.

In summary, *Dt*HNL’s novelty and properties open perspectives for the development of a new class of biocatalysts. With our work, we extend the knowledge about HCN release strategies in nature and associate a new function to the Bet v1 superfamily.

The discovery approach reported herein can speed up the process to identify additional enzymes with hydroxynitrile lyase function significantly. Tedious purification steps are avoided, and there is no need for degenerate primers for the amplification of the genes from gDNA or cDNA, which can be a problematic issue for organisms with big genomes. Finally, the concept can be extended to any enzyme of interest, as long as a suitable enzymatic assay is available. Thus, it gives the opportunity to identify novel sequences for a desired function in a reasonable time and the restriction to identify related proteins is no longer a limitation.

## Methods

### General

The fern *Davallia tyermannii* was purchased in a local shop. *Pteridium aquilinum* leaves and croziers were harvested from a local forest (Styria, Austria). Racemic mandelonitrile was purchased from abcr GmbH & Co. KG. (*R*)-Mandelonitrile was purchased from Sigma-Aldrich or kindly donated by DSM Fine Chemicals Austria. All other chemicals were purchased from Sigma-Aldrich or Carl Roth GmbH, if not stated otherwise. Material for molecular biology and protein analysis was obtained from Thermo Fisher Scientific or Promega, if not specifically mentioned. Gibson Assembly^®^ enzymes were purchased from New England Biolabs and BioZym. ÄKTA purifier (GE Healthcare) was employed for protein purification. Protein purification columns were purchased from GE Healthcare. For protein electrophoresis, an XCell SureLock^®^ Mini-Cell equipped with a PowerEase^®^ 500 Programmable Power Supply (Thermo Fisher Scientific) was used. Spectrophotometric measurements were performed with a Synergy Mx plate reader (BioTek) or Cary Series Agilent Technologies spectrophotometer. The nucleotide sequence of proteins reported herein have been submitted to GenBank (Supplementary Table 12).

### Transcriptome generation and sequencing

The total RNA was isolated from *Davallia tyermannii* and *Pteridium aquilinum* following the protocols provided by the Spectrum™ Plant Total RNA Kit (Sigma Aldrich) and RNAqueous^®^ Kit (Ambion^®^, Thermo Fisher Scientific), respectively. Quality assessment to ensure RNA integrity was performed with an Agilent 2100 Bioanalyzer (Agilent Technologies) and agarose gel electrophoresis (1% agarose gel, running conditions: 80 V, 40 min). Normalized transcriptome sequencing was obtained by the commercial service from Microsynth AG as follows: library generation for the 454 FLX sequencing was carried out according to standard protocols (Roche/454 life sciences, Branford, CT 06405, USA). The concatenated inserts were sheared randomly by nebulization to fragments ranging in size from 400 bp to 900 bp. These fragments were end polished and the 454 A and B adaptors that are required for the emulsion PCR and sequencing were ligated to the ends of the fragments. The resulting fragment libraries were sequenced on both halves of a picotiterplate on the GS FLX using the Roche/454 Titanium chemistry. Sequence data can be accessed via the EMBL-EBI European Nucleotide Archive under the study accession number PRJEB10896 (*D. tyermannii*) and PRJEB10897 (*P. aquilinum*).

High-quality reads were selected using Newbler sequence filtering at default settings. The quality controlled reads were assembled into individual isotigs using the Roche/454 Newbler software (454 Life Sciences Corporation, version 2.6.0) with default settings (minimum read length 20, duplicate reads excluded, expected depth 0, seed step 12, seed length 16, seed count 1, minimum overlap length 40 bp, minimum overlap identity 90%, alignment identity score 2, alignment difference score −3).

### Protein isolation from fern leaves and purification

Disruption of *D. tyermannii* leaves using the P-PER^™^ Plant Protein Extraction Reagent was chosen for routine protein isolation according to the manufacturer’s protocol. PD-10 desalting columns (GE Healthcare) were used for buffer exchange (50 mM sodium phosphate buffer, pH 5.7). The partial purification of the HNLs was carried out by using anion exchange chromatography (HiTrap QFF 1 mL column, from HiTrap IEX Selection Kit). The column was previously equilibrated with 20 mM sodium phosphate buffer, pH 5.7. The elution was performed with the following parameters: gradient from 0 to 1 M NaCl in 20 column volumes, flow 1 mL/min and 1 mL elution fractions were collected. All purification fractions were tested for HNL activity using Feigl-Anger test paper^30^ in a 384-well plate in 100 mM citrate buffer pH 4.0 and 3 mM racemic mandelonitrile as the substrate. The mixture was incubated for 20 min. Finally, pH of positive fractions was determined with a pH indicator. The fractions between 100 and 200 mM NaCl elution showed HNL activity. Each active fraction was concentrated 10 times through centrifugation via MCWO 10 Vivaspin 500 (Sartorius) and the buffer was exchanged to 25 mM potassium phosphate buffer, pH 6.0. Protein samples were stored at −20 °C.

### BN PAGE and in gel HNL activity detection

Specifically, 15 μL of each concentrated purification fraction or flow through or 2 μL of total protein extract were applied on a NativePAGE^™^ Novex^®^ 4–16% Bis-Tris protein gel, and HNL activity assay was performed after the electrophoretic run. The procedure was performed similar to that described previously^33^. Afterwards, the gel was stained by silver staining as described^50^ with the following modifications: fix step over-night and 30 min incubation in water after the first ethanol wash step, in order to recover the original gel dimensions, because the gel reduced its size after incubation in ethanol. The gel was stored in 12% acetic acid. Bands of interest were excised and stored at −20 °C in 10% ethanol.

### Mass spectrometry and transcriptome data integration

Excised protein bands were tryptically digested, dissolved in 0.1% formic acid and separated by nano-RP-HPLC using a 70 min gradient. The samples were ionized in the nanospray source equipped with nanospray tips and analyzed in a Thermo LTQ-FT mass spectrometer operated in positive ion mode, applying alternating full scan MS (*m/z* 400 to 2,000, 50,000 resolution) in the ion cyclotron and MS/MS by collision induced dissociation of the five most intense peaks in the ion trap with dynamic exclusion enabled.

The LC-MS/MS data were analyzed by searching the translated *D. tyermannii* transcriptome and known contaminants with Proteome Discoverer 1.3 and Mascot 2.3 (1% FDR, min. two rank 1 peptides with min. Mascot ion score 20 and precursor mass error lower than 10 ppm required for protein identification). Hits were subjected to BLAST alignment against NCBI non-redundant public protein database.

### Isoenzyme gene isolation

Genomic DNA from *D. tyermannii* was isolated with the PowerPlant^®^ Pro DNA Isolation Kit (MO BIO Laboratories Inc.) according to the provided manual. Specific primers were designed for amplification of the isoenzymes (Supplementary Table 13) and genes were amplified by PCR. PCR products were isolated from a 1% agarose gel after electrophoresis and sequenced (LGC Genomics). Results obtained by Sanger and Roche/454 sequencing were compared and corrected in case of inconsistency.

### Cloning

The list of primers, gBlocks^®^ Gene Fragments and synthetic genes is reported in Supplementary Tables 13 and 14. Electrocompetent *E. coli* strains as described below were transformed by electroporation and positive clones were selected on LB agar plates with the appropriate antibiotics. *E. coli* TOP 10F’ strain was used for vector amplification and protein expression after transformation of pMS470 vector. *E. coli* BL21 Star (DE3) strain was employed for expression of *Dt*HNL1–4.

#### Isotigs screening

Synthetic genes were ordered after codon optimization for expression in *E. coli* (GeneArt^®^ Gene synthesis, Thermo Fisher Scientific). The genes were cloned into the pMS470 vector (*Nde*I/*Hind*III). Clones were selected on ampicillin (100 mg/L).

#### DtHNL1 expression

A synthetic gene of isotig02643 was cloned into the pEHISTEV^51^ vector (*Nco*I/*Hind*III), in order to add the His-TEV tag at the *N*-terminus of the protein. Clones were selected on kanamycin (50 mg/L).

#### DtHNL2, 3 and 4 expression

Genes amplified from gDNA were cloned into the pJET1.2 vector (CloneJET PCR Cloning Kit, Thermo Fisher Scientific) and clones were selected on ampicillin (100 mg/L). Plasmids were isolated and used as a template for a second PCR with primers designed for cloning into the pEHISTEV vector (*Nco*I/*Hind*III), in order to add the His-TEV tag at the *N*-terminus of the protein. Clones were selected on kanamycin (50 mg/L). Sequences were confirmed by Sanger sequencing (LGC Genomics).

#### DtHNL1 and PtaIso02775 mutants

Optimized nucleotide sequences coding for parts of the protein and containing the desired mutation(s) were purchased as gBlock^®^ Gene Fragments (Integrated DNA Technologies). pEHISTEV containing *Dt*HNL1 or *Pta*Iso02775 was amplified with appropriate primers and the two fragments (Vector and the specific gBlock^®^) were assembled by the Gibson Assembly^®^ Method. Clones were selected on kanamycin (50 mg/L). The sequences were confirmed by Sanger sequencing (Microsynth AG).

### Protein Expression

Protein expression was performed in shake flasks in LB medium supplemented with the specific antibiotic at 37 °C. Induction was performed by addition of 0.5 mM IPTG at OD_600_ 0.7, followed by incubation at 25 °C for 20 hours. After the cultivation, cell pellets were suspended in the appropriate buffer (*Isotig screening*: 50 mM potassium phosphate buffer, pH 6.0. *Protein purification*: 20 mM sodium phosphate, 0.5 M NaCl, 10 mM imidazole, pH 7.4).

### Protein purification

Cells were disrupted by sonication (80% duty cycle, 7 output, 6 min). Affinity chromatography was performed with a HisTrap FF 5 mL column with standard protocol at 4 °C. Start buffer: 20 mM sodium phosphate, 0.5 M NaCl, 10 mM imidazole at pH 7.4. Elution buffer: 20 mM sodium phosphate, 0.5 M NaCl, 500 mM imidazole, pH 7.4. Elution method: gradient from 0 to 100% elution buffer, 20 column volumes. Fractions containing *Dt*HNL were combined and desalted (HiPrep 26/10 desalting column). Purified protein fractions were stored in 50 mM sodium phosphate pH 6.5, at −80 °C.

Size-exclusion chromatography was performed with ÄKTA Avant 25 (GE Healthcare) equipped with a Superdex 200 10/300 GL column (GE Healthcare) at 4 °C. The column was pre-equilibrated with 150 mM NaCl, 10 mM Tris-HCl pH 8. The protein (0.5 ml; 1 mg/ml in 10 mM Tris-HCl pH 8) was loaded onto a column with a flow rate of 0.1 mL/min. The absorbance of the eluent was monitored at 280 and 254 nm. A Gel-Filtration-Standard (BioRad) was diluted 10x and separated under the same conditions.

### Determination of *Dt*HNL activity

#### Standard assay

The enzymatic activity was quantified as described^52^. The standard reaction was carried out in 96-well plates in 50 mM sodium citrate-phosphate buffer pH 5.0 and 15 mM racemic or (*R*)-mandelonitrile as the substrate, previously dissolved in 3 mM sodium citrate-phosphate buffer pH 3.5. Control reactions contained storage buffer instead of protein. Benzaldehyde formation was detected at 280 nm for 10 min. One unit is defined as the amount of enzyme that catalyzes the formation of 1 μmol of benzaldehyde in 1 min.

*Activity at different pH values* was performed using the following 50 mM buffers: HCl-potassium chloride pH 2.0 and 2.5; sodium citrate-phosphate pH 2.5–6.5.

*Michaelis-Menten* curves were determined at standard conditions using concentrations of (*R*)-mandelonitrile from 0.009 mM to 18 mM and 0.01 μg of *Dt*HNL. *K*_m_ and *v*_max_ were calculated on basis of non-linear regression using Sigma Plot^™^ 11.0. Obtained values are based on the average of three independent experiments.

Activity at different temperatures. Determination of the optimal reaction temperature was performed in cuvettes. A final volume of 1 mL contained 100 μL of purified protein and 700 μL of pre-warmed 50 mM citrate phosphate buffer, pH 5.0. The reaction was initiated by the addition of 200 μL of substrate solution (60 mM racemic mandelonitrile in 3 mM citrate phosphate buffer, pH 3.5). Enzymatic activity was measured from 10 to 50 °C at 280 nm for 10 min.

### Enzyme stability

#### pH stability

1 mg/mL of purified protein was incubated in 50 mM sodium citrate phosphate buffer pH 2.5, 4.0 at 5–8 °C. At certain time-points, an aliquot of enzyme was diluted to 0.01 mg/mL. HNL activity was measured under standard conditions.

### Rapid qualitative hydroxynitrile lyase assay

The assay detects the cyanogenesis reaction and is based on detection of released HCN *via* a Feigl–Anger test paper^30^. Generally, the reaction was performed in 100 mM sodium citrate buffer at pH 4.5 with 13 mM mandelonitrile. The reaction was carried out at room temperature until blue spots were detected.

### SeMet-*Dt*HNL1 expression and purification

*E. coli* BL21 (DE3) Star expressing tagged *Dt*HNL1 were cultivated in a shake flask in minimal medium (M9 salts 5X, 2% (w/v) glucose, 2 mM MgSO_4_, 0.01 mg/mL thiamine, 0.01 mg/mL FeCl_3_) supplemented with 50 mg/L kanamycin and 50 mg/L selenomethionine. Induction was performed at OD_600_ 0.5 by addition of 0.5 mM IPTG, and the culture was incubated at 25 °C for 38 hours. After harvesting and cell disruption, SeMet-*Dt*HNL1 was purified by affinity chromatography (NiSepharose 6 Fast Flow resin, GE Healthcare). Elution was performed with 20 mM sodium phosphate, 0.5 M NaCl, 300 mM imidazole, pH 7.4. Fractions containing SeMet-*Dt*HNL1 were combined and desalted (PD10 Desalting columns, GE Healthcare). Protein was stored at −20 °C in 50 mM potassium phosphate buffer pH 6.0. For crystallization, the buffer was exchanged to 10 mM Tris-HCl pH 8 by diluting and re-concentrating the enzyme in 2 mL Ultra Centrifugal Filters (Amicon). The final protein concentration of native *Dt*HNL1 was 4 mg ml^−1^ and of SeMet *Dt*HNL1 was 3 mg ml^−1^.

### Crystallization and structure determination

Crystallization experiments were performed with an ORYX 8 robot (Douglas Instruments) using the sitting drop vapor-diffusion method in 96-well plates at 16 °C. Screening was performed using commercial screens Morpheus Screen MD 1–46, JCSG + MD1–37 (Molecular Dimensions) and Index HT HR2–144 (Hampton Research). Optimization of crystallization conditions was performed manually by the sitting drop vapor-diffusion method in Crystal Clear Duo crystallization frames at 16 °C.

Native crystals of *Dt*HNL1 were obtained by mixing 0.5 μl 4 mg/mL protein sample (in 10 mM Tris-HCl pH 8.0) with 1 μl reservoir solution (0.9 M NaNO_3_; Na_2_HPO_4_; (NH_4_)_2_SO_4_ mix, 0.1 M Tris-bicine buffer pH 8.5 and 30% (w/v) polyethylene glycol monomethyl ether 550 & polyethylene glycol 20k; Morpheus condition C9). Additionally, native crystals were also grown by mixing 1 μl 4 mg/mL protein sample (in 10 mM Tris-HCl pH 8.0) with 0.5 μl reservoir solution (0.1 M 2-(4-(2-hydroxyethyl)-1-piperazinyl) ethanesulfonic acid pH 7.5 and 10% (w/v) polyethylene glycol; JSCG condition B4). Crystals appeared after 2–3 days. SeMet-*Dt*HNL1 crystals were obtained in 0.2 M sodium thiocyanate, 20% (w/v) polyethylene glycol 3350. A 1:1 ratio of protein and screening solutions was used, using protein concentration of 3 mg/mL (in 10 mM Tris-HCl pH 8.0). Crystals appeared within 2–3 days. After supplementation of 30% glycerol, the crystals were flash-cooled in liquid-nitrogen.

Soaking experiments were performed with the native *Dt*HNL1 crystals (grown as described above). Crystalline 4-hydroxybenzaldehyde - HBA, (*R*)-mandelonitrile – MXN or benzoic acid – BEZ were added to a crystallization drop with a small CryoLoop. After an incubation period of 30 s, 1 min, 5 min and 15 min, crystals were harvested, flash-cooled in liquid nitrogen and used for data collection.

All datasets were collected at 100 K at beamlines ID29 and BM14 at the ESRF (Grenoble, France) and at beamline XRD1 at Elettra (Trieste, Italy). Data were processed using the XDS program package^53^ or iMosflm^54^/SCALA^55^. The AutoSol Program^56^,^57^ and the AutoBuild Program^58^ from the PHENIX software suit^59^ were used to define the selenium heavy metal-atom sites using a SeMet-*Dt*HNL1 SAD data set, as well as to build an initial model. The resulting model was completed manually in Coot^60^ and refined with PHENIX. Difference electron density too large for a water molecule was observed in the putative active site of the enzyme (Supplementary Fig. 7a), which could not be fitted using known buffer components or compounds of the crystallization conditions. Therefore, we did not interpret this portion of the electron density.

For all datasets from soaked crystals, molecular replacement was performed with Phaser-MR^56^. The previously obtained SeMet-*Dt*HNL structure was used as a search template. The resulting model was completed manually in Coot and refined with PHENIX. The occupancies of the ligands refined to values of 70–80%. Final structures were validated using Molprobity^61^. Detailed data processing and structure refinement statistics are summarized in Supplementary Table 6. The atomic coordinates and structure factors have been deposited in the Protein Data Bank under the accession codes 5E46 (*Dt*HNL1 SeMet), 5E4B (*Dt*HNL1-MXN), 5E4D (*Dt*HNL1–BEZ), 5E4M (*Dt*HNL1-HBA).

### Cyanohydrin Synthesis

Synthesis of cyanohydrins was carried out in a biphasic system as described in detail by Wiedner *et al*.^52^, except for 1-phenylethanone which was used in 0.3 M concentration. The aqueous phase contained 3 mg of purified *Dt*HNL1. After acetylation, samples were analyzed by isothermal GC at 110 °C for 20 min. Retention times: internal standard triisopropylbenzene (IS) 1.6 min; 1-phenylethanone 4.1 min; 2-hydroxy-2-phenylpropanenitrile acetate 9.1 min. A negative control reaction (non-enzymatic background) was set up in the same conditions, with buffer instead of the enzyme solution. Apparent kinetic parameters were determined with 0.05 mg of *Dt*HNL1 (final concentration 0.1 mg/mL). Different concentrations of benzaldehyde were used (500–10 mM), while the amount of HCN was constant (2 M). The specific activity was obtained by determination of (*R*)-mandelonitrile formation during the first 30 min. In a biphasic system the benzaldehyde is predominantly in the organic phase and the *de facto* substrate concentration in the buffer phase was calculated by determination of the partition coefficient MTBE-H_2_O of benzaldehyde. Each reaction was performed in duplicate as two different independent experiments. *K*_m_ and *v*_max_ were calculated on basis of non-linear regression by using Sigma Plot^™^ 11.0.

## Additional Information

**How to cite this article:** Lanfranchi, E. *et al*. Enzyme discovery beyond homology: a unique hydroxynitrile lyase in the Bet v1 superfamily. *Sci. Rep.* **7**, 46738; doi: 10.1038/srep46738 (2017).

**Publisher's note:** Springer Nature remains neutral with regard to jurisdictional claims in published maps and institutional affiliations.

## Supplementary Material

Supporting Information

Supplementary Datasets

## Figures and Tables

**Table 1:** Synthesis of cyanohydrins with *Dt*HNL1.

Entry	Substrate		0.5 hours		2 hours		6 hours		24 hours	
	→		conv	*ee*	conv	*ee*	conv	*ee*	conv	*ee*
	Product		%	%	%	%	%	%	%	%
**1**	benzaldehyde → (*2R*)-2-hydroxy-2-phenylacetonitrile	***Dt*****HNL1**	95.1	99.0	97.4	94.5	97.9	85.9	99.5	61.4
		Control	−4.9	–	11.7	0.8	18.5	0.3	39.7	0.5
**2**	2-chlorobenzaldehyde → (*2R*)-2-(2-chlorophenyl)-2-hydroxyacetonitrile	***Dt*****HNL1**	38.6	89.4	75.6	92.1	96.6	88.4	99.6	71.3
		Control	11.3	−14.0	19.6	−5.7	28.7	−2.3	59.8	−1.5
**3**	3-phenylpropanal → (*2R*)-2-hydroxy-4-phenylbutanenitrile	***Dt*****HNL1**	50.9	45.8	84.8	48.3	94.5	48.1	93.2	48.0
		Control	18.0	0.4	36.3	0.2	53.0	0.0	80.2	0.2
**4**	3-phenylprop-2-enal → (*2R*)-2-hydroxy-4-phenylbut-3-enenitrile	***Dt*****HNL1**	5.3	82.3	46.6	95.2	88.2	94.5	98.2	92.6
		Control	−17.0	–	−11.6	–	−12.6	–	−10.5	–
**5**	furan-2-carbaldehyde → (*2S*)-furan-2-yl-hydroxyacetonitrile	***Dt*****HNL1**	95.3	99.3	98.0	98.9	98.0	97.6	98.1	92.9
		Control	10.5	0.0	20.5	0.4	35.9	0.1	71.1	−0.1
**6**	1-phenylethanone → 2-hydroxy-2-phenylpropanenitrile	***Dt*****HNL1**	9.8	n.d.	17.3	n.d.	23.5	n.d.	22.3	n.d.
		Control	6.6	–	6.6	–	6.9	–	7.0	–

The synthesis of cyanohydrins was performed with 3 mg of *Dt*HNL1. 0.5 M aldehyde or 0.3 M of ketone were mixed with 2 M HCN in TBME (final volume 1 mL), 2% v/v triisopropylbenzene was added as internal standard. To monitor the non-enzymatic formation of cyanohydrins, independent control reactions were set up at the same conditions, but omitting the enzyme (Control). Reaction conditions: pH 4.0, 10 °C and 1,000 rpm. Samples were analyzed by GC after acetylation of the product. Conversion (conv) is based on the substrate consumption. Entry 5: change of product configuration as a consequence of the Cahn–Ingold–Prelog rule. Dashes indicate that no product peak was detected. n.d., not determined.
