# Erratum: Enzyme discovery beyond homology: a unique hydroxynitrile lyase in the Bet v1 superfamily

**DOI:** 10.1038/srep46834

**Published:** 2017-05-26

**Authors:** Elisa Lanfranchi, Tea Pavkov-Keller, Eva-Maria Koehler, Matthias Diepold, Kerstin Steiner, Barbara Darnhofer, Jürgen Hartler, Tom Van Den Bergh, Henk-Jan Joosten, Mandana Gruber-Khadjawi, Gerhard G. Thallinger, Ruth Birner-Gruenberger, Karl Gruber, Margit Winkler, Anton Glieder

Scientific Reports 7: Article number: 4673810.1038/srep46738; published online: 05 03 2017; updated: 05 26 2017

The original version of this Article contained errors. In the Introduction section,

“Finally there is a number of characterized HNLs with yet unpublished amino acid sequences and protein folds, for example, *Pat*HNL (*Prunus amygdalus turcomanica*)^25^, *Pars*HNL (*Prunus armeniaca* L.)^26^, *Pe*HNL (*Passiflora edulis*)^27^ (sequence information for PeHNL is public since December 2016, see Ref. 28), and the fern HNL from *Phlebodium aureum (Pha*HNL)^28^”.

should read:

“Finally there is a number of characterized HNLs with yet unpublished amino acid sequences and protein folds, for example, *Pat*HNL (*Prunus amygdalus turcomanica*)^25^, *Pars*HNL (*Prunus armeniaca* L.)^26^, *Pe*HNL (*Passiflora edulis*)^27^ (sequence information for PeHNL is public since December 2016, see Ref. 28), and the fern HNL from *Phlebodium aureum (Pha*HNL)^29^”.

In addition, in the Results section,

“Mandelonitrile was chosen, as this is the natural cyanohydrin identified in the genus *Davallia* and different other fern genera^28,31,32^”.

should read:

“Mandelonitrile was chosen, as this is the natural cyanohydrin identified in the genus *Davallia* and different other fern genera^29,31,32^”.

