# Supplementary material for: Enzyme discovery beyond homology: a unique hydroxynitrile lyase in the Bet v1 superfamily
Source: Sci Rep. 2017 May 3;7:46738. doi: 10.1038/srep46738 (PMC5413884; doi:10.1038/srep46738)
Supplement: Supporting Information [file srep46738-s1.pdf]

Enzyme discovery beyond homology: a unique hydroxynitrile lyase in the Bet v1 superfamily

Elisa Lanfranchi, Tea Pavkov-Keller, Eva-Maria Koehler, Matthias Diepold, Kerstin Steiner, Barbara Darnhofer, Jürgen Hartler, Tom Van Den Bergh, Henk-Jan Joosten, Mandana Gruber-Khadjawi, Gerhard G. Thallinger, Ruth Birner-Gruenberger, Karl Gruber, Margit Winkler*, Anton Glieder

Supplementary Information

Supplementary Result 1. Screening for HNL activity in cyanogenic ferns

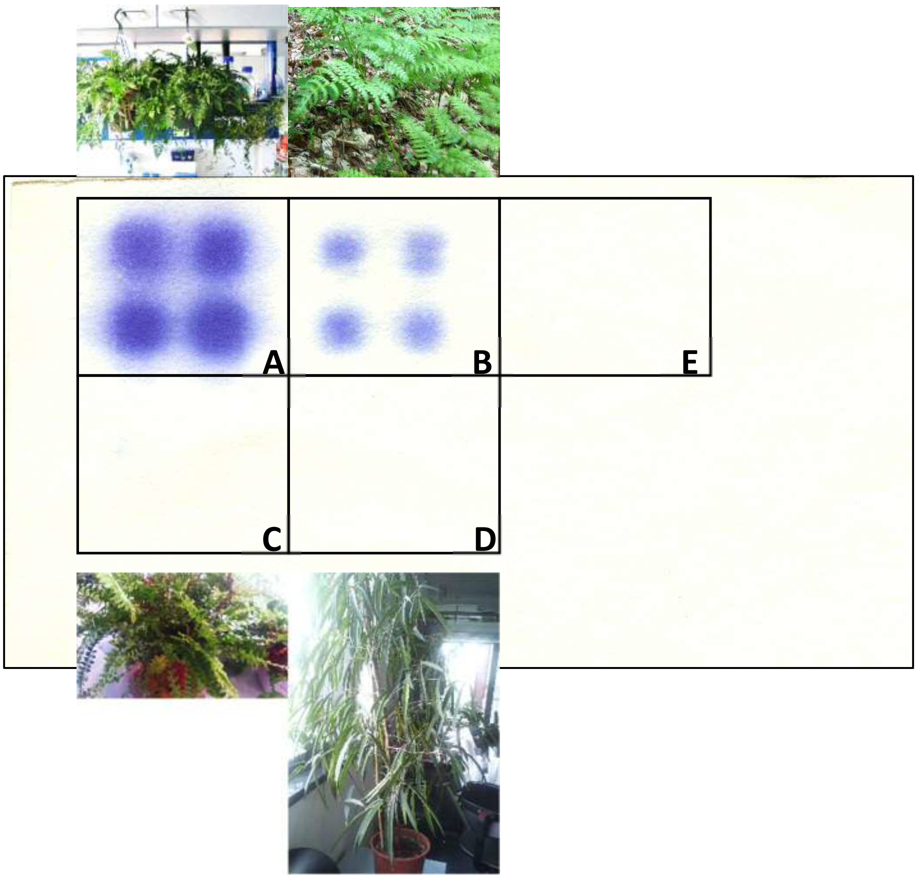

Supplementary Figure 1a. HNL activity screening in different plants.

Two cyanogenic ferns *Davallia tyermannii* and *Pteridium aquilinum* were screened for hydroxynitrile lyase (HNL) activity. Fresh leaves were disrupted and the resultant protein extract was mixed with 100 mM citrate buffer pH 4.5 containing racemic mandelonitrile. Cyanide release was detected via a Feigl–Anger test paper.[1]

Davallia tyermannii (A); *Pteridium aquilinum* (B). Negative controls: Non cyanogenic fern from *Nephrolepsis* genus (C); Non cyanogenic plant from *Ficus* genus (D). Blank: 100 mM citrate-phosphate buffer pH 4.5 (E).

Supplementary Result 2. Transcriptome sequencing and assembly

Poly A mRNA was isolated from fresh tissues (leaves and croziers) of the two fern species *P. aquilinum* and *D. tyermannii.* The major results are summarized in Supplementary Table 1 and Supplementary Figure 2a-2h.

Supplementary Table 1. Summary of transcriptome sequencing and assembly.

	P. aquilinum		***D. tyermannii***	
	**Number**	**% of total**	**Number**	**% of total**
Total number of reads	834,642	-	560,161	-
Reads after Newbler quality control	828,772	100.0	557,396	100.0
Reads aligned	657,753	79.4	447,265	80.2
Reads assembled	526,742	53.6	291,066	52.2
Reads partially assembled	101,168	12.2	155,950	28.0
Singletons	69,936	8.4	48,729	8.7
Contigs	78,726	-	15,964	-
Isogroups	18,357	-	7,792	-
Isotigs	48,207	-	11,497	-
Average isotig length	568.6		808.5	-
Median isotig length	514.0	-	737.0	-
Largest isotig length	2,019	-	2,772	-

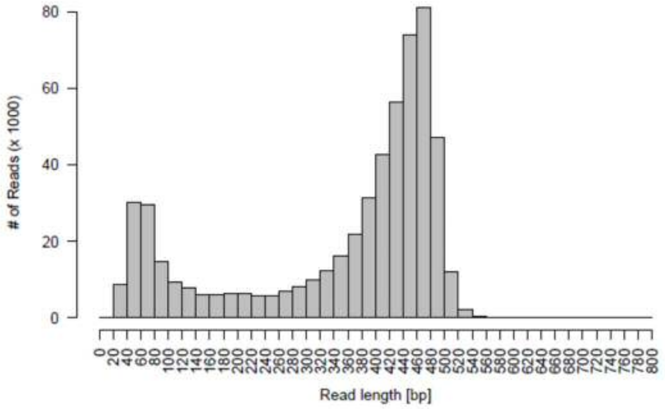

Supplementary Figure 2a. *D. tyermannii* transcriptome read length distribution.

Total reads: 560161; average/median length: 350.5/418.0.

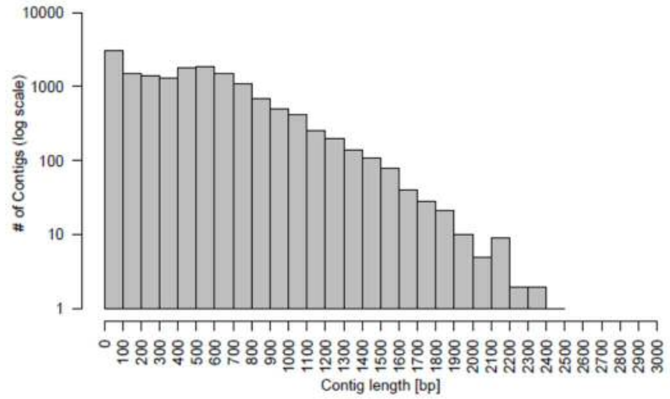

Supplementary Figure 2b. *D. tyermannii* transcriptome contig length distribution.

Total contigs: 15964; average/median length: 466.0/454.0.

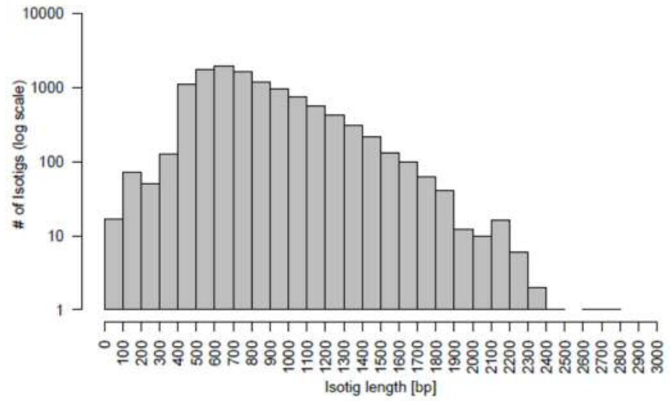

Supplementary Figure 2c. *D. tyermannii* transcriptome isotig length distribution.

Total isotigs 11497; average/median length: 808.5/737.0.

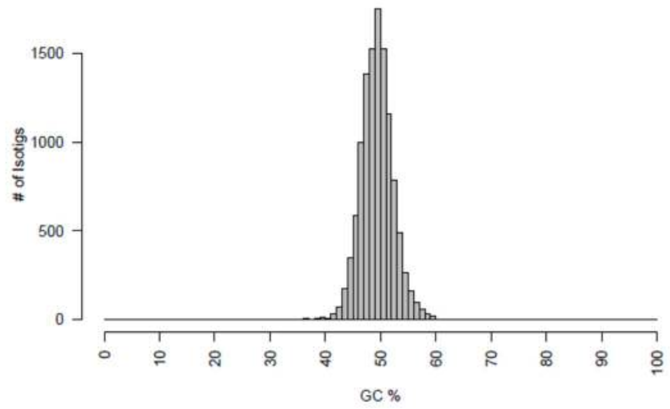

Supplementary Figure 2d. *D. tyermannii* transcriptome isotig GC content distribution.

Total isotigs: 11497; average/median: %: 49.4/49.4.

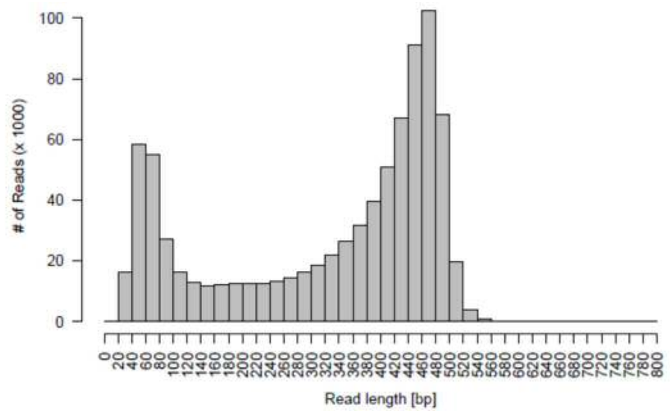

Supplementary Figure 2e. *P. aquilinum* transcriptome read length distribution.

Total reads: 834642: average/median length: 325.6/395.0.

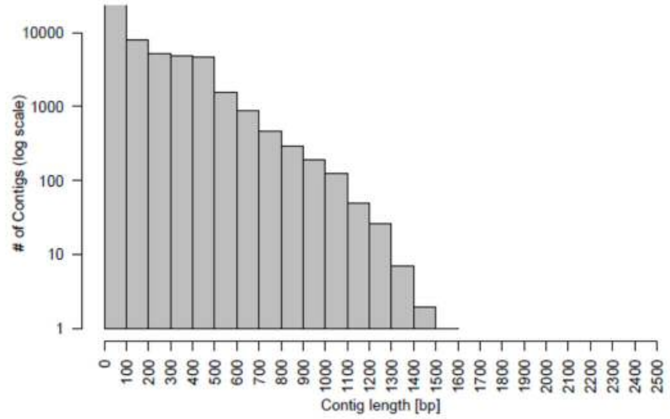

Supplementary Figure 2f. *P. aquilinum* transcriptome contig length distribution.

Total contigs 78726; average/median length: 129.8/40.0.

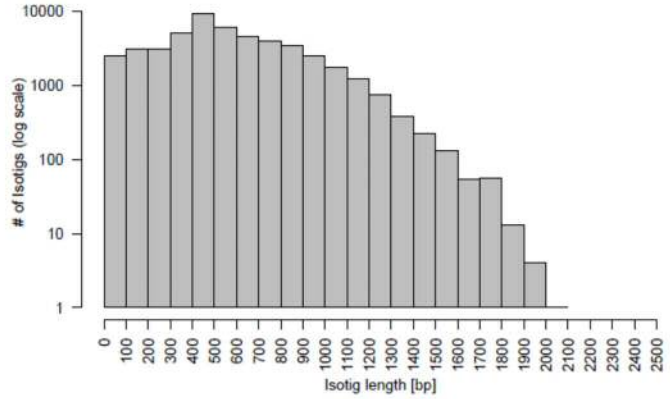

Supplementary Figure 2g. *P. aquilinum* transcriptome isotig length distribution.

Total isotigs: 48207: average/median: 568.6/514.0.

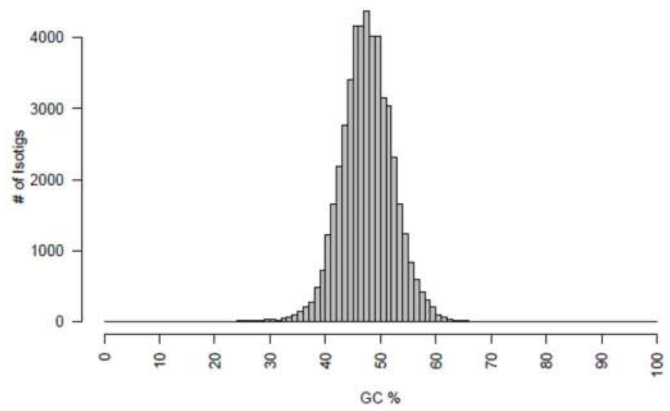

Supplementary Figure 2h. *P. aquilinum* transcriptome isotig GC content distribution.

Total isotigs: 48207; average/median %: 47.6/47.5.

Supplementary Result 3. *In silico* HNL search in fern transcriptomes

10 known HNL sequences were subjected to a tblastn search in both transcriptomes of *D. tyermannii* and *P. aquilinum*, respectively, using CLC Main Workbench 7.6 (QIAGEN Aarhus A/S), with the default parameters of the software (program: tblastn; expectation value: 100; word size: 3; mask lower case: no; filter low complexity: yes; maximum number of hits: 500; protein matrix and gap costs: BLOSUM62 existence 11 extension 1; number of threads: 1; genetic code: 1). Sequence queries are summarized in Supplementary Table 2).

All hits show low sequence identity and/or low sequence coverage (Supplementary Fig. 3a-3t). This bioinformatic analysis indicated that generally a new HNL type(s) might exist in ferns, as already claimed by Wajant and and coworkers.[2]

Supplementary Table 2. List of HNLs employed as sequence queries.

Entry	UniProtKB	ID	Organism	Protein Family (pfam database)
1	Q95K2	*Pa*HNL	*Prunus amygdalus*	GMC oxidoreductase
2	P52706	*Ps*HNL	*Prunus serotina*	GMC oxidoreductase
3	B7YF77	*Ej*HNL	*Eriobotrya japonica*	GMC oxidoreductase
4	Q9LFT6	*At*HNL	*Arabidopsis thaliana*	Alpha/beta hydrolase
5	P52704	*Hb*HNL	*Hevea brasiliensis*	Alpha/beta hydrolase
6	P52705	*Me*HNL	*Manihot esculenta*	Alpha/beta hydrolase
7	D1MX73	*Bm*HNL	*Baliospermum montanum*	Alpha/beta hydrolase
8	E8WN5	*Gt*HNL	*Granulicella tundricola*	Cupin
9	P52708	*Sb*HNL	*Sorghum bicolor*	Peptidase S10
10	P93243	*Lu*HNL	*Linum usitatissimum*	Zinc binding alcohol dehydrogenase

Supplementary Figures 3a–3t. Qualitative visualization of the tblatn results for each query in *D. tyermannii* (Supplementary Fig. 3a–3j) and *P. aquilinum* (Supplementary Fig. 3k–3t) transcriptomes. First 20 hits are depicted. 0% 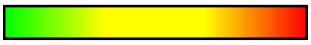 100% Identity

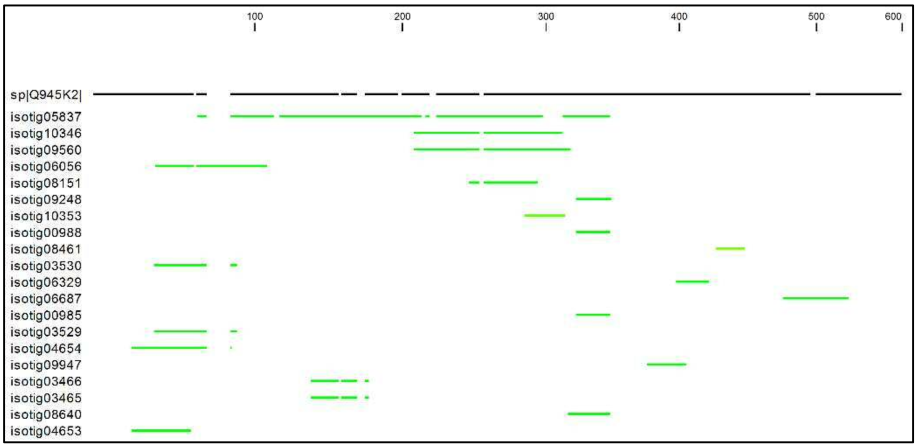

Supplementary Figure 3a. tblastn *Pa*HNL – *D. tyermannii* transcriptome.

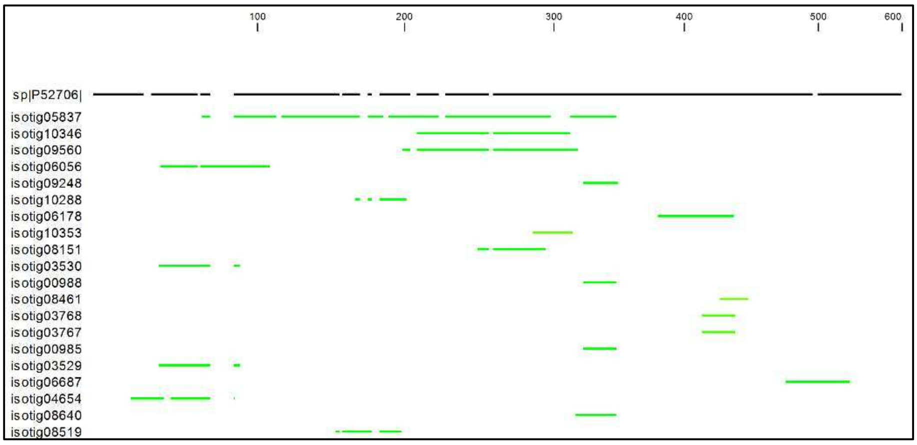

Supplementary Figure 3b. tblastn *Ps*HNL – *D. tyermannii* transcriptome.

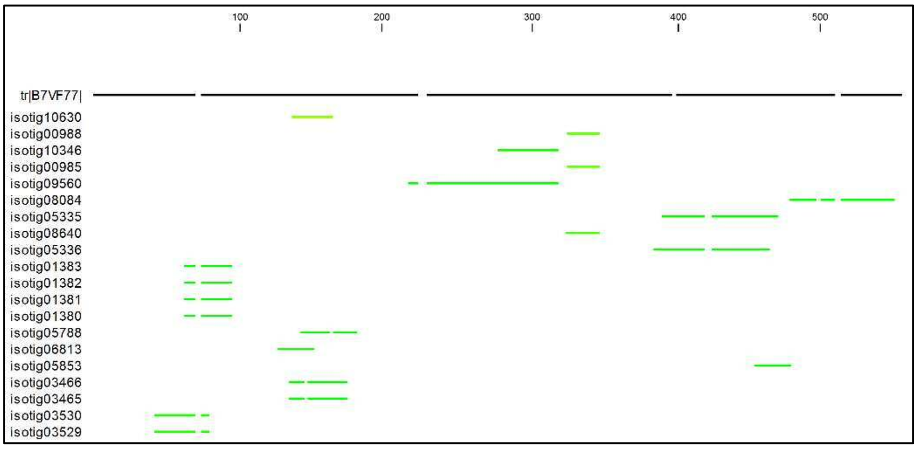

Supplementary Figure 3c. tblastn *Ej*HNL – *D. tyermannii* transcriptome.

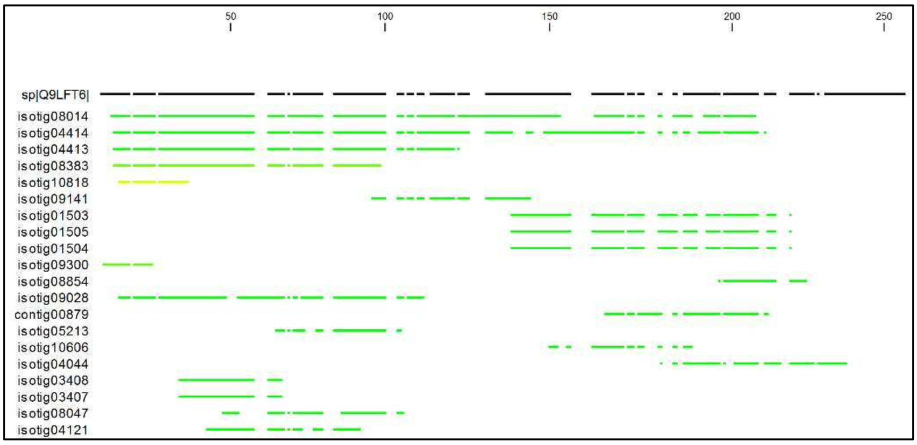

Supplementary Figure 3d. tblastn *At*HNL – *D. tyermannii* transcriptome.

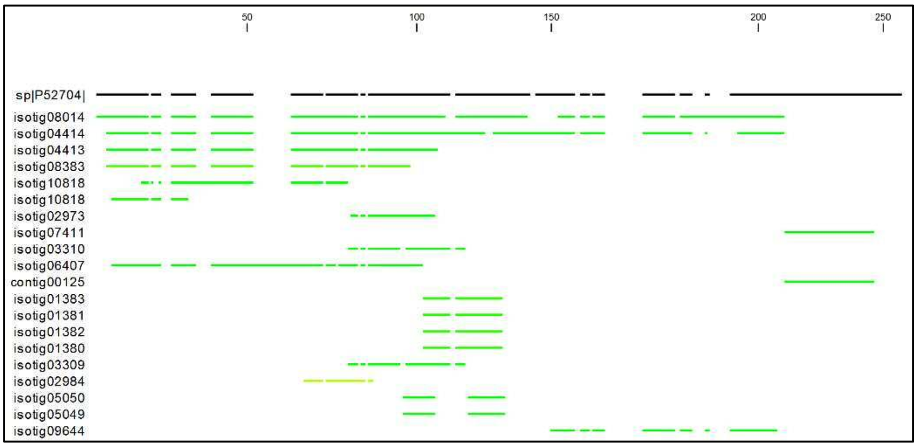

Supplementary Figure 3e. tblastn *Hb*HNL – *D. tyermannii* transcriptome.

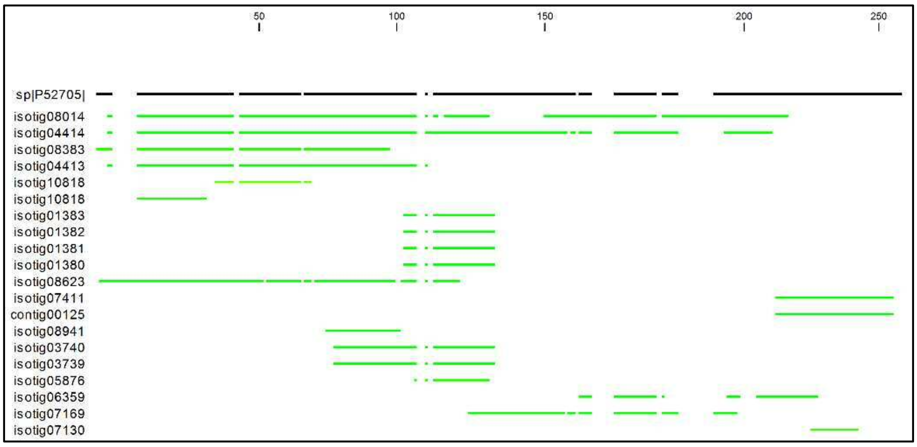

Supplementary Figure 3f. tblastn *Me*HNL – *D. tyermannii* transcriptome.

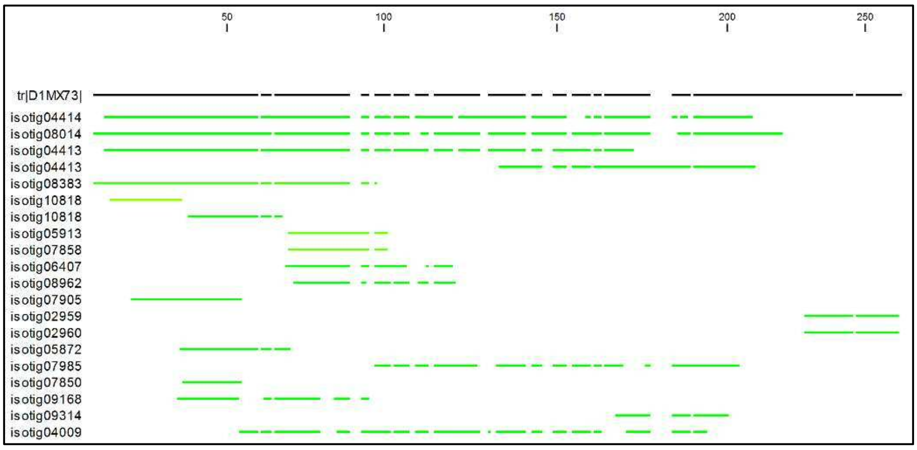

Supplementary Figure 3g. tblastn *Bm*HNL – *D. tyermannii* transcriptome.

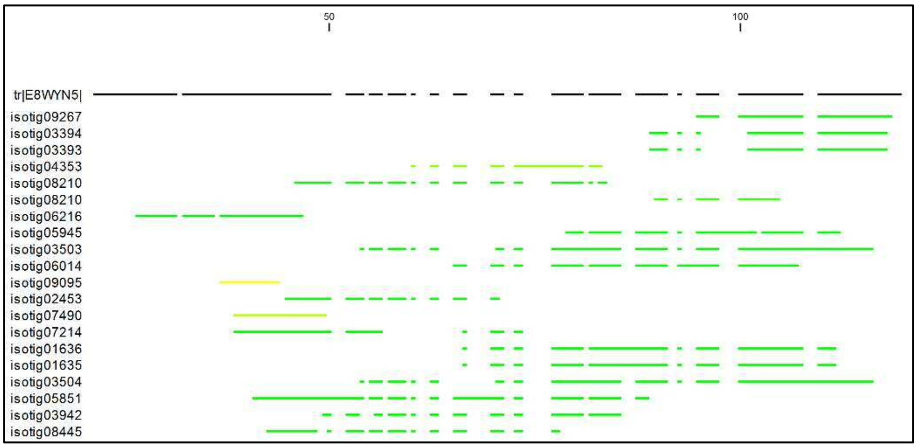

Supplementary Figure 3h. tblastn *Gt*HNL – *D. tyermannii* transcriptome.

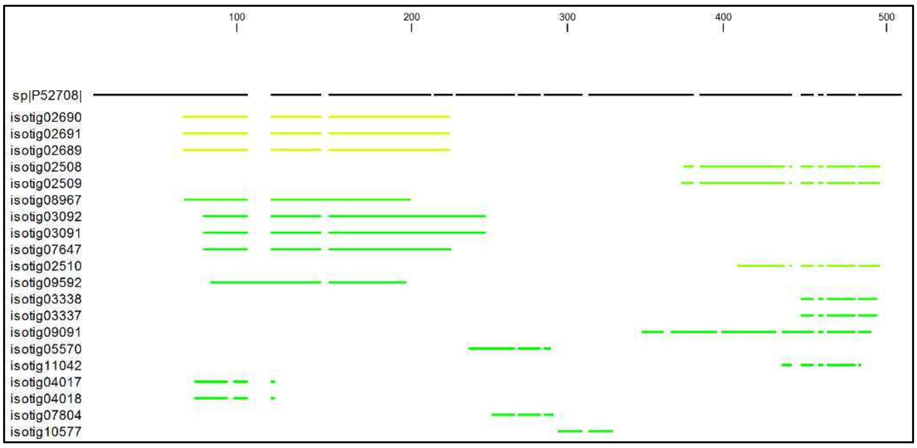

Supplementary Figure 3i. tblastn *Sb*HNL – *D. tyermannii* transcriptome.

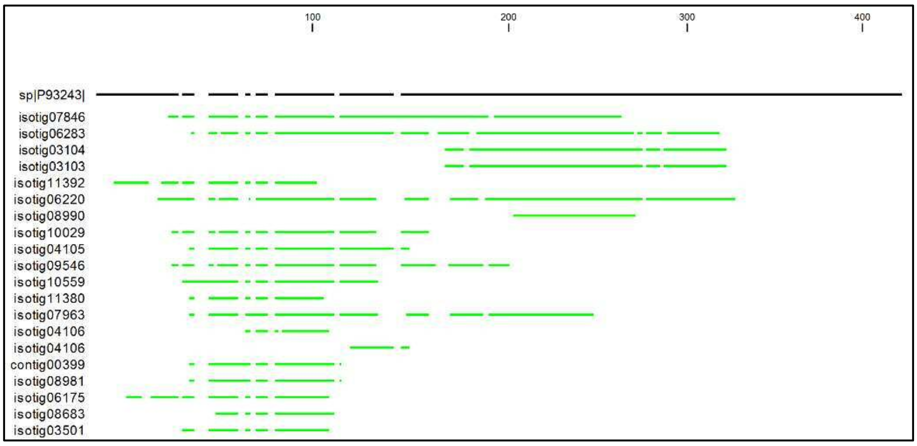

Supplementary Figure 3j. tblastn *Lu*HNL – *D. tyermannii* transcriptome.

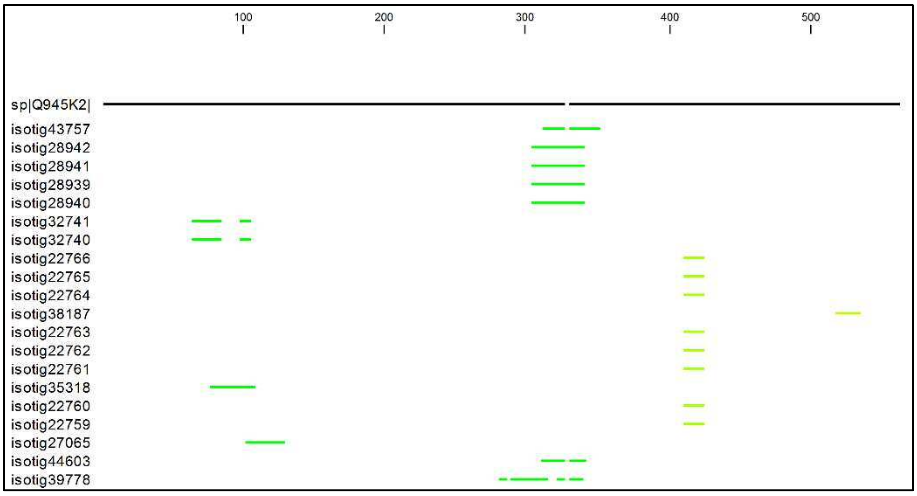

Supplementary Figure 3k. tblastn *Pa*HNL – *P. aquilinum* transcriptome.

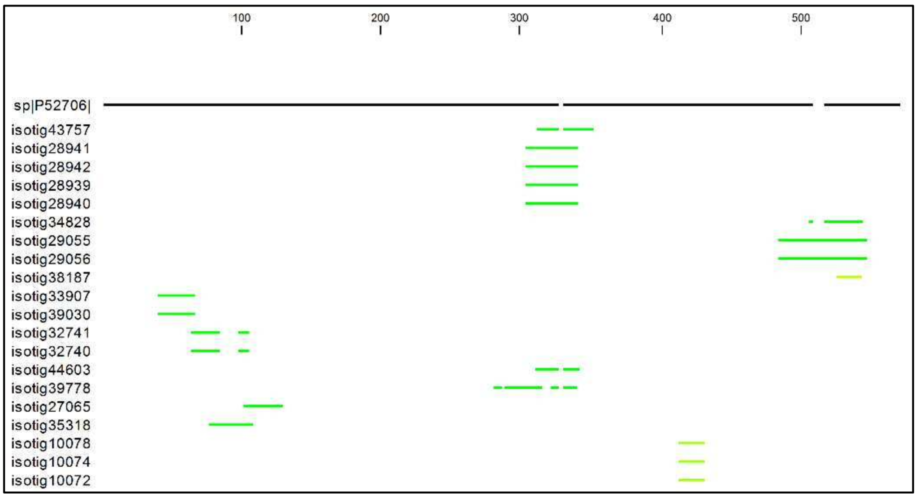

Supplementary Figure 3l. tblastn *Ps*HNL – *P. aquilinum* transcriptome.

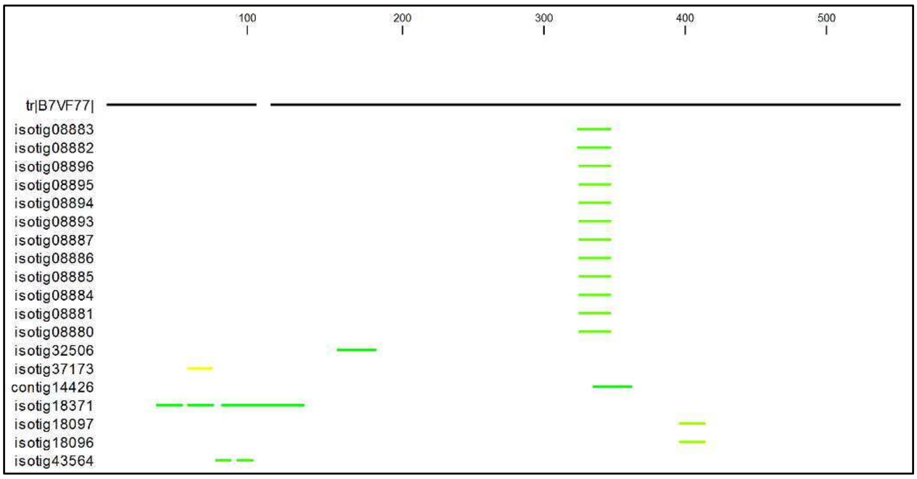

Supplementary Figure 3m. tblastn *Ej*HNL – *P. aquilinum* transcriptome.

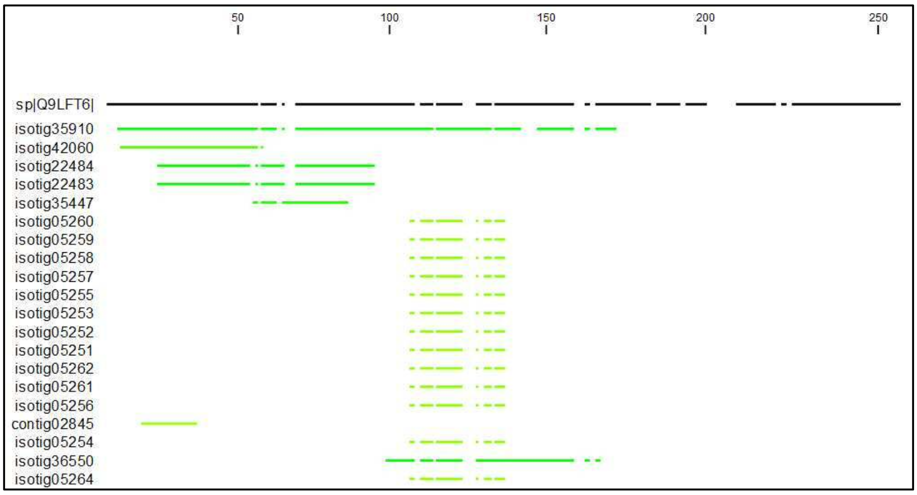

Supplementary Figure 3n. tblastn AtHNL – *P. aquilinum* transcriptome.

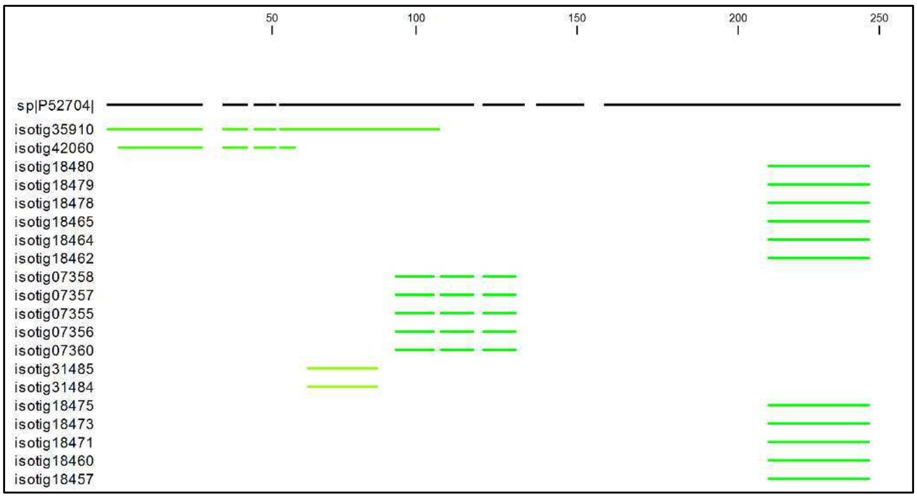

Supplementary Figure 3o. tblastn *Hb*HNL – *P. aquilinum* transcriptome.

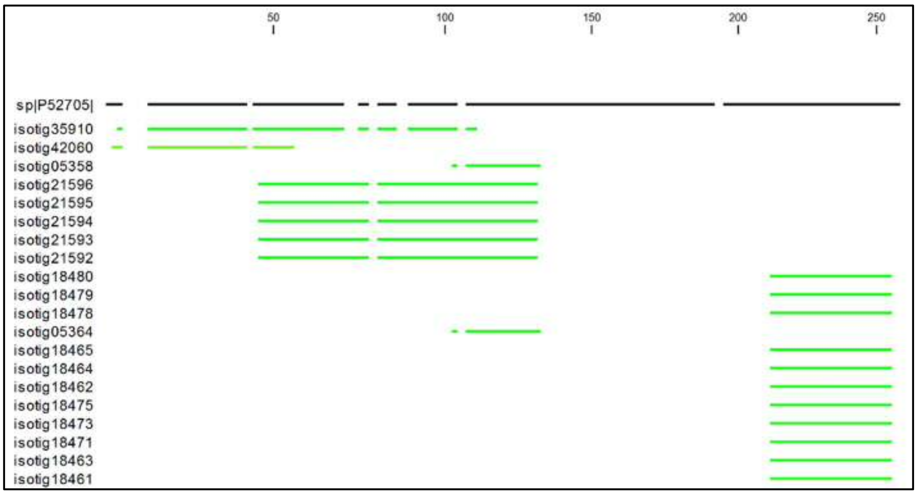

Supplementary Figure 3p. tblastn *Me*HNL – *P. aquilinum* transcriptome.

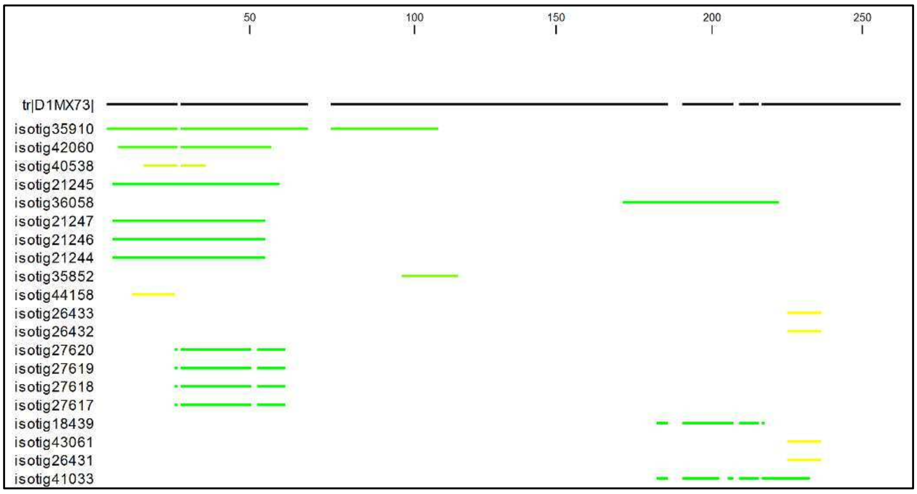

Supplementary Figure 3q. tblastn *Bm*HNL – *P. aquilinum* transcriptome.

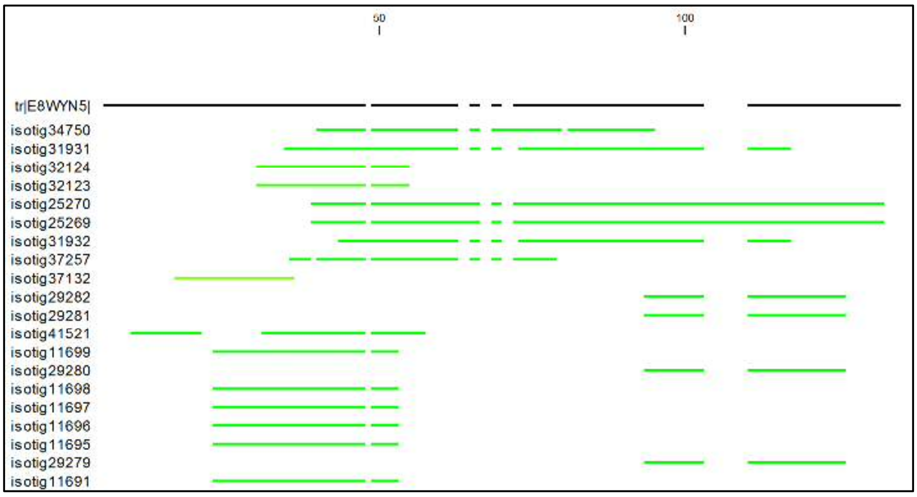

Supplementary Figure 3r. tblastn *Gt*HNL – *P. aquilinum* transcriptome.

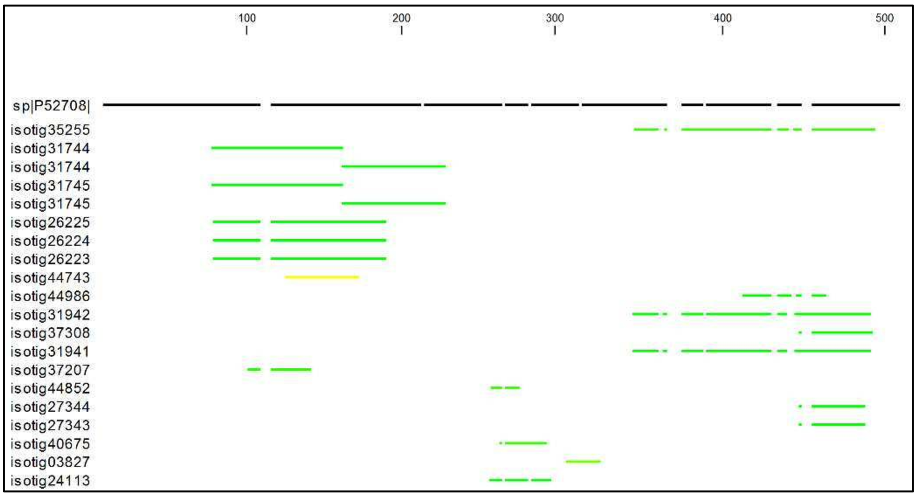

Supplementary Figure 3s. tblastn *Sb*HNL – *P. aquilinum* transcriptome.

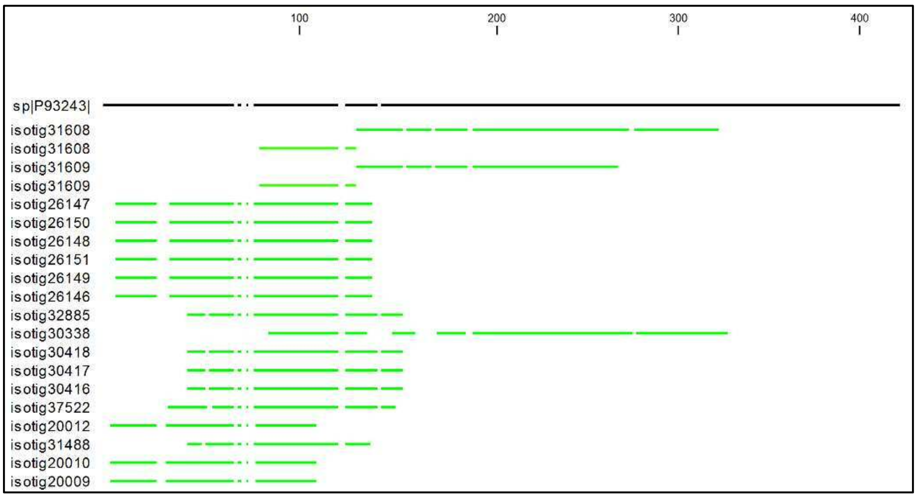

Supplementary Figure 3t. tblastn *Lu*HNL – *P. aquilinum* transcriptome.

Supplementary Result 4. Mass Spectrometry

Supplementary Table 3. Summary of the proteins screened for HNL activity

	Entry	1	2	3	4	5	6
	**ID**	**Isotig 02643**	**Isotig 06604**	**Isotig 07200**	**Contig 00505**	**Isotig 04065**	**Isotig 04379**
	**Family members**	Isotig 02641<br>Isotig 07602<br>Contig 00751			Contig 00644<br>Contig 00096	Isotig 04066<br>Isotig 07043	Isotig 04380
**MS/MS**	Σ# Unique Peptides	5	8	3	1	2	2
	Σ# PSMs	32	13	3	8	4	3
**Blastp (ncbi)**	Best Hit	XP_009405224.1<br>Predicted Lachrymatory factor synthase like	NP_001051733.1<br>Os03g0822200	XP_0068559057.2<br>Predicted plasma membrane associated cation binding protein 1	XP_010555234.1<br>Predicted thaumatin-like protein 1b	XP_002313728.1<br>Disease resistance responsive family protein	XP_002971933.1<br>Hypothetical protein SELMODRAFT 270941
	Identity	28	77	43	35	48	49
	Query coverage	72	100	100	96	55	93
	E value	$8e^{-12}$	$6e^{-141}$	$1e^{-35}$	$1e^{-20}$	$6e^{-19}$	$2e^{-68}$
	**pfam**	Polyketide Cyc2	NAD binding 10	DREPP	Thaumatin	Dirigent	Thioredoxin 4

Supplementary Result 5. *Dt*HNL isoenzymes

Supplementary Table 4. Sequence confirmation.

Genes were amplified by PCR from the isolated gDNA and analyzed by Sanger sequencing. None of them contained introns. Except for *Dt*HNL2, the derived amino acid sequences were identical to the one obtained from the translated transcriptome. The observed difference in case of *Dt*HNL2 might be a mistake originating from error prone reverse transcription or during transcriptome sequencing.

***Dt*HNL**	**Transcriptome**	**Genome**
	**aa**	**aa**
*Dt*HNL1	Identical	
*Dt*HNL2	GGVSIF (130-136)	GGGVIF (130-136)
*Dt*HNL3	Identical	
*Dt*HNL4	Identical	

```
DtHNL1          MAGTGGGAEQFQLRGVLWGKAYSWKITGTTIDKVWSIVGDYVRVDNWVSSVVKSSHVVSG 60
DtHNL2          MAGTRGGAEEFQLRGVLWGKAYSWKITGTTIDKVWSIVGDYVRVDNWVSSVVKSSHVVSG 60
DtHNL3          MAGTGGGAEEFQLRGVLWGKAYSWKISGTTIDKVWAIVGDYVRVDNWVSSVVKSSHVVSG 60
DtHNL4          MAGTGGGAEEFQLRGVLWGKAYSWKITGTTIDKVWSIVGDYVRVDNWVSSVVKSSHVVSG 60
                **** ****:****************:********:************************

DtHNL1          EANQTGCVRRFVCYPASEGESETVDYSELIHMNAAAHQYMYMIVGGNITGFSLMKNYVSN 120
DtHNL2          DANQTGCVRRFVCYPASDGESETVDYSELIHMNAAAHQYMYMIVGGNITGFSLMKNYVSN 120
DtHNL3          DANKTGCVRRFVCYPASEGESETVDYSELIHMNAAAHQYMYMIVGGNITGFSLMKNYVSN 120
DtHNL4          DANKTGCVRRFVCYPASEGESETVDYSELIHMNAAAHQYMYMIVGGNITGFSLMKNYVSN 120
                :**:*************:******************************************

DtHNL1          ISLSSLPEEDGGGVIFYWSFTAEPASNLTEQKCIEIVFPLYTTALKDLCTHLSIPESSVT 180
DtHNL2          ISLSSLPEEDGGGVIFYWSFTAEPASNLTEQKCIEIVFPLYTTALKDLCTHLSIPESSVT 180
DtHNL3          ISLNSLPEADGGGVILHWSFTAEPASNLTEQKCIEIVFPLYTTALKDLCTHLSIPESSVT 180
DtHNL4          ISLNSLPEADGGGVIFHWSFTAEPASNLTEQKCIEIVFPLYTTALKDLCTHLSIPESSVT 180
                ***.**** ******::*******************************************

DtHNL1          LLDD 184
DtHNL2          LLDD 184
DtHNL3          LLGD 184
DtHNL4          LLGD 184
                **.*
```

Supplementary Figure 5a. Multiple sequence alignment of *Dt*HNL isoenzymes.

Highlighted residues: differences (red); conserved residues in *Dt*HNL1 and *Dt*HNL2 only (magenta); conserved residues in *Dt*HNL3 and *Dt*HNL4 only (green). Protein sequence alignment was performed with Clustal Omega.[3]

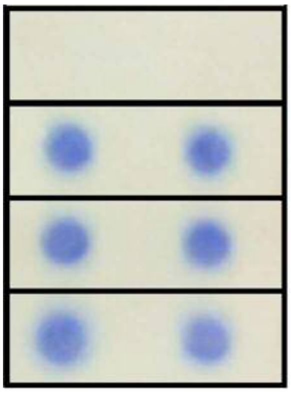

Supplementary Figure 5b. HNL activity assay for *Dt*HNL2, 3 and 4.

Each protein was expressed by *E. coli* with standard cultivation protocol as described in Online Methods. Cells were disrupted with BugBuster^{TM} Protein extraction reagent (Novagen) according to the provided manual. 50 µL of clear protein lysate was mixed with 100 µL 50 mM sodium citrate – phosphate buffer pH 5.0 and 50 µL of racemic mandelonitrile, previously dissolved in 3 mM sodium citrate – phosphate buffer pH 3.5 (8 µL/mL). Negative control: 50 µL BugBuster™ Protein extraction reagent (Novagen). Cyanide release was detected throughout a Feigl–Anger test paper.[1] All three isoenzymes displayed similar activity.

Supplementary Result 6. *Dt*HNLs characterization

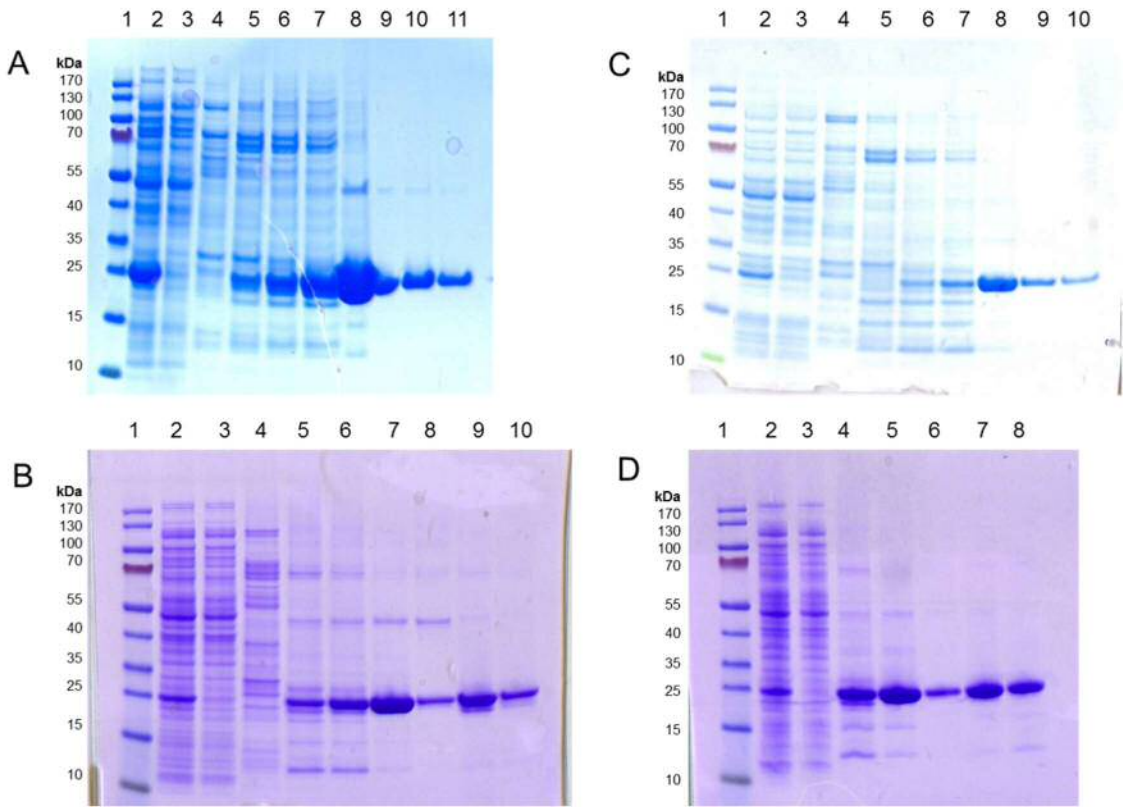

Supplementary Figure 6a. SDS PAGE of *Dt*HNL purification.

*Dt*HNL isoenzymes with N-terminal HIS-tags were purified by affinity chromatography as described in Online Methods. Molecular weight standard: PageRuler^{TM} Prestained Protein Ladder (Thermo Fisher Scientific) (1). Purification of *Dt*HNL1 (A): cell free lysate (2); flow through (3); elution fractions (4-11). Purification of *Dt*HNL2 (B): cell free lysate (2); flow through (3); elution fractions (4-8); desalted fractions (9, 10). Purification of *Dt*HNL3 (C): cell free lysate (2); flow through (3); elution fractions (4-10). Purification of *Dt*HNL4 (D): cell free lysate (2); flow through (3); elution fractions (4-6); desalted fractions (7, 8). Expected molecular weight of His-tagged *Dt*HNL isoenzymes is approximately 23 kDa.

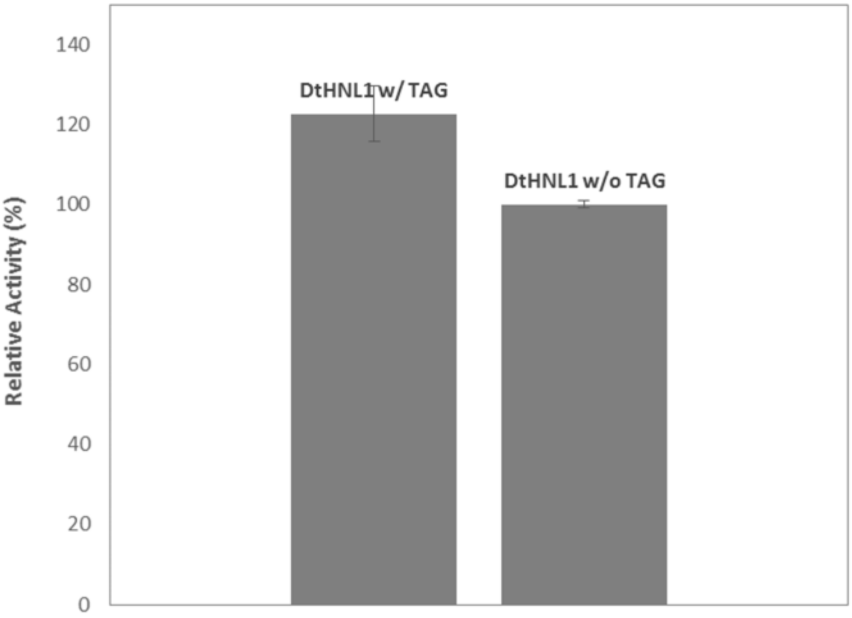

Supplementary Figure 6b. Specific activity of HisTEV-*Dt*HNL1 *vs* untagged *Dt*HNL1.

0.5 mg of purified HisTEV-*Dt*HNL1 were incubated with TEV protease (His tagged protein, recombinantly expressed and purified by Ni-affinity chromatography). The reaction was carried out over night at 4°C. The obtained untagged *Dt*HNL1 was separated from the remaining non-cleaved protein and TEV protease by Ni-affinity chromatography. Pure *Dt*HNL1 eluted in the flow through, whereas HisTEV-*Dt*HNL1 and TEV protease bind to the nickel resin. Specific activity of tagged and untagged pure *Dt*HNL1 were determined by the standard assay. The tag in *N*-terminal position does not negatively affect *Dt*HNL1 activity (*Dt*HNL1 w/TAG). Therefore, all experiments reported in this work were performed with the purified HisTEV tagged enzymes, which have been named *Dt*HNL1-4 for convenience.

Supplementary Table 5. *Dt*HNL pH stability

Enzyme stability at different pH conditions was performed as described in Online Methods. Additionally, pH 5.0 (50 mM sodium citrate phosphate buffer) and pH 6.5 (50 mM sodium phosphate buffer) were tested. Standard deviations are based on the average of three or two samples, each of which are obtained from the average of three or two independent technical triplicates. Relative activity is based on the specific activity at time 0 of each samples.

***Dt*HNL1**								
**Time of incubation**	**pH 2.5**		**pH 4.0**		**pH 5.0**		**pH 6.5**	
**[h]**	**Relative activity**		**Relative activity**		**Relative activity**		**Relative activity**	
	**%**	**SD**	**%**	**SD**	**%**	**SD**	**%**	**SD**
0	100	0	100	0	100	0	100	0
2	89	12	92	13	87	15	96	18
4	107	18	110	13	117	20	123	22
8	99	6	94	8	105	22	103	27
24	75	4	64	22	96	11	100	21
48	n.d.	n.d.	38	18	53	14	52	16
72	52	13	28	5	75	1	70	14
***Dt*HNL2**								
**Time of incubation**	**pH 2.5**		**pH 4.0**		**pH 5.0**		**pH 6.5**	
**[h]**	**Relative activity**		**Relative activity**		**Relative activity**		**Relative activity**	
	**%**	**SD**	**%**	**SD**	**%**	**SD**	**%**	**SD**
0	100	0	100	0	100	0	100	0
2	111	10	100	2	119	6	111	15
4	126	19	114	11	125	10	117	13
8	109	5	98	5	105	18	94	3
24	82	8	96	3	95	4	104	7
48	63	7	103	5	90	13	110	3
72	27	8	102	6	104	1	103	1

*Dt*HNL3								
**Time of incubation**	**pH 2.5**		**pH 4.0**		**pH 5.0**		**pH 6.5**	
**[h]**	**Relative activity**		**Relative activity**		**Relative activity**		**Relative activity**	
	**%**	**SD**	**%**	**SD**	**%**	**SD**	**%**	**SD**
0	100	0	100	0	100	0	100	0
2	n.d.	n.d.	n.d.	n.d.	n.d.	n.d.	n.d.	n.d.
4	99	13	99	14	116	14	115	6
8	99	20	100	2	94	3	106	13
24	89	15	93	14	89	15	86	14
48	73	3	97	12	88	3	87	2
72	62	7	87	9	81	5	86	17
*Dt*HNL4								
**Time of incubation**	**pH 2.5**		**pH 4.0**		**pH 5.0**		**pH 6.5**	
**[h]**	**Relative activity**		**Relative activity**		**Relative activity**		**Relative activity**	
	**%**	**SD**	**%**	**SD**	**%**	**SD**	**%**	**SD**
0	100	0	100	0	100	0	100	0
2	94	7	95	1	95	14	112	0
4	100	3	101	3	99	7	120	11
8	94	11	78	8	85	3	111	22
24	59	10	87	10	84	8	107	4
48	31	3	87	11	83	10	102	13
72	n.d.	n.d.	70	11	67	5	74	2

Supplementary Result 7. *Dt*HNL1 Structure

Supplementary Table 6. Data-collection and processing statistics.

Statistics for the highest-resolution shell are shown in parentheses.

	***Dt*HNL1-SeMet**	***Dt*HNL1-MXN (10 sec)**	***Dt*HNL1-BEZ (1 min)**	***Dt*HNL1-HBA (15 min)**
Wavelength (Å)	0.9790	0.9184	0.9184	0.9184
Resolution range (Å)	57.97-1.85 (1.92-1.85)	35.8-1.50 (1.55-1.50)	57.93-1.85 (1.90-1.85)	36.76-1.80 (1.86-1.80)
Space group	*I*222	*I*222	*I*222	*I*222
Unit cell parameters (Å, °)	73.63, 94.02, 117.05 90, 90, 90	73.36, 94.14, 116.14 90, 90, 90	73.62, 93.87, 116.17 90, 90, 90	73.51, 93.47, 117.86 90, 90, 90
Total reflections	462708 (43863)	260107 (15412)	240033 (17990)	265397 (18210)
Unique reflections	34750 (3382)	63070 (5168)	34309 (3230)	37654 (3567)
Multiplicity	13.3 (13.0)	4.1 (3.0)	7.0 (5.6)	7.0 (5.1)
Completeness (%)	99.84 (98.60)	97.48 (80.88)	98.55 (93.69)	99.27 (95.25)
Mean I/σ(I)	9.97 (2.57)	13.74 (1.86)	13.72 (2.35)	16.66 (1.84)
Wilson B-factor	20.10	14.55	13.27	17.12
R-merge	0.188 (0.796)	0.066 (0.611)	0.150 (0.862)	0.108 (0.782)
R-meas	0.195	0.075	0.162	0.116
CC1/2	0.995 (0.825)	0.998 (0.713)	0.995 (0.925)	0.998 (0.634)
CC*	0.999 (0.951)	1 (0.912)	0.999 (0.98)	0.999 (0.881)
R-work	0.155 (0.212)	0.157 (0.270)	0.180 (0.383)	0.165 (0.294)
R-free	0.192 (0.270)	0.180 (0.277)	0.235 (0.470)	0.198 (0.345)
Number of non-hydrogen atoms	3399	3500	3287	3294
macromolecules	2932	2924	2830	2856
ligands	-	36	45	54
water	467	540	412	384
Protein residues	351	352	351	354
RMS(bonds)	0.007	0.007	0.007	0.007
RMS(angles)	1.02	1.03	1.01	1.00
Ramachandran favored (%)	97	98	98	97
Ramachandran allowed (%)	3	2	2	3
Ramachandran outliers (%)	0	0	0	0
Average B-factor	21.00	19.80	17.60	21.10
macromolecules	19.20	17.30	16.20	19.60
ligands	-	18.30	22.70	32.10
solvent	32.20	33.20	26.40	31.10
PDB code	5E46	5E4B	5E4D	5E4M

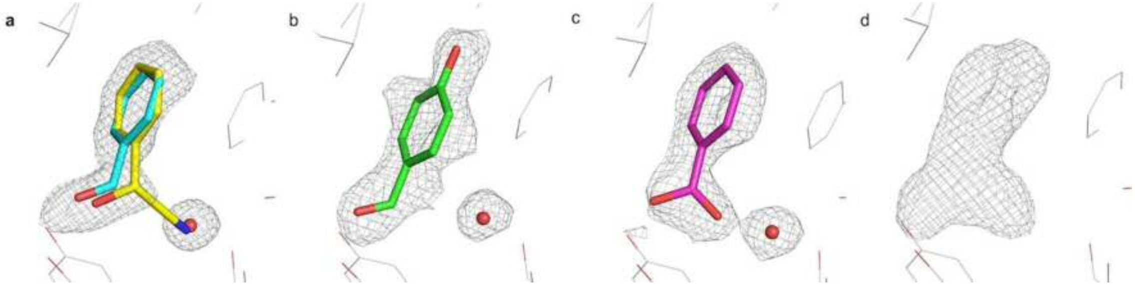

Supplementary Figure 7a. Electron density maps.

Fo-Fc omit density within the active site (contoured at 2σ) of the *Dt*HNL1 complexes with *(R)*-mandelonitrile (yellow) / benzaldehyde (cyan) (**a**), 4-hydroxybenzaldehyde (green) (**b**) and benzoic acid (magenta) (**c**); and for the native structure (**d**). Amino acid residues are shown as grey lines, the bound ligands as sticks, and water molecule as red sphere. The figure was prepared using the program PyMOL (Schrodinger Inc.).

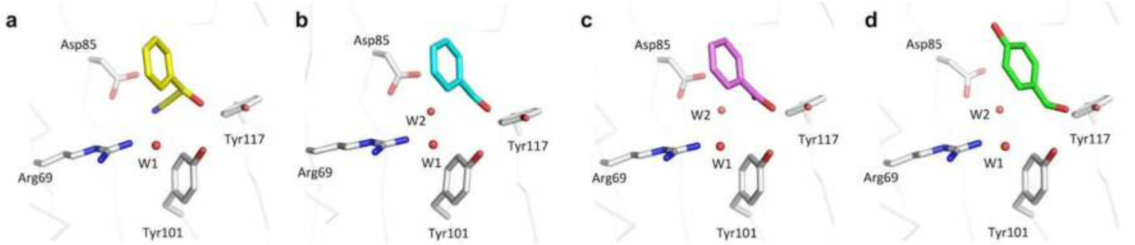

Supplementary Figure 7b. Ligand binding site of all determined *Dt*HNL1 complex structures.

Amino acid residues are shown in grey sticks, the bound ligands in yellow (*(R)*-mandelonitrile), cyan (benzaldehyde), magenta (benzoic acid) and green (4-hydroxybenzaldehyde) sticks, and the water molecules as red spheres. The figure was prepared using the program PyMOL (Schrodinger Inc.).

Supplementary Result 8. Reaction mechanism and *Dt*HNL1 mutants.

Supplementary Table 7. Summary of specific activity of *Dt*HNL1 mutants.

Standard activity assay was performed with the purified mutants in the presence of 15 mM racemic mandelonitrile pH 5.0 at 25°C. Benzaldehyde formation was followed at 280 nm. n.d.: activity was not determined, due to insoluble expression of the protein.

Residue	Mutation	Activity	Activity Loss
		U/mg	%
R69	A69	n.d.	n.d.
R69	K69	n.d.	n.d.
D85	A85	1.5 ± 0.1	≥99
S87	A87	29 ± 1	91
Y101	A101	n.d.	n.d.
Y101	F101	0	100
Y117	A117	n.d.	n.d.
Y117	F117	≤ 0.5	≥99
Y161	A161	n.d.	n.d.
Y161	F161	26 ± 3.8	92
D85-S87	S85-D87	≤ 0.5	≥99

Supplementary Result 9. HNL from *P. aquilinum*

Supplementary Table 8. *Dt*HNL1 tblastn in *P. aquilinum* transcriptome (Accession no. PRJEB10897)

A tblastn search was performed with CLC Main Workbench 7.6.2 (QIAGEN Aarhus A/S), with the default parameters reported by the software (program: tblastn; expectation value: 100; word size: 3; mask lower case: no; filter low complexity: yes; maximum number of hits: 50; protein matrix and gap costs: BLOSUM62 existence 11 extension 1; number of threads: 1; genetic code: 1).

Entry	Hit	E-value	Score	Overlap	Identity	Positive	Gaps	Note
					%	%	%	
**1**	isotig02778	1.39E-39	350	285.3261	41	62.28571	4.571429	
**2**	isotig02777	1.41E-39	350	285.3261	41	62.28571	4.571429	Identical to entry 1
**3**	isotig02776	1.58E-39	350	285.3261	41	62.28571	4.571429	Identical to entry 1
**4**	isotig02775	1.58E-39	351	285.3261	41	62.28571	4.571429	
**5**	isotig02773	1.69E-39	350	285.3261	41	62.28571	4.571429	Identical to entry 1
**6**	isotig02771	1.71E-39	351	285.3261	41	62.28571	4.571429	Identical to entry 4
**7**	isotig02774	1.78E-39	350	285.3261	41	62.28571	4.571429	Identical to entry 1
**8**	isotig02772	1.81E-39	350	285.3261	41	62.28571	4.571429	Identical to entry 1
**9**	isotig02770	2.49E-39	351	285.3261	41	62.28571	4.571429	Identical to entry 4
**10**	isotig02779	3.38E-32	296	265.7609	39	60.7362	4.907975	Not full protein
**11**	contig56214	7.2E-22	191	180.9783	33	54.95495	4.504505	Mistakes in the sequence
**12**	contig56214	7.2E-22	96	107.6087	28	59.09091	0	
**13**	isotig32801	3.83E-08	118	210.3261	30	45.65217	12.31884	Low sequence query coverage
**14**	isotig32800	7.55E-08	117	203.8043	30	45.52239	12.68657	
**15**	isotig32290	3.29E-07	112	143.4783	27	52.17391	4.347826	
**16**	isotig32289	5.03E-07	112	143.4783	27	52.17391	4.347826	
**17**	isotig35067	6.26E-07	112	171.1957	26	47.16981	3.773585	

Supplementary Table 9. *Dt*HNL1 pblastn in gametophyte transcriptome of *P. aquilinum*.[4]

A tblastn search was performed with CLC Main Workbench 7.6.2 (QIAGEN Aarhus A/S), with the default parameters reported by the software (program: tblastn; expectation value: 100; word size: 3; mask lower case: no; filter low complexity: yes; maximum number of hits: 50; protein matrix and gap costs: BLOSUM62 existence 11 extension 1; number of threads: 1; genetic code: 1).

**Entry**	**Hit**	**E-value**	**Score**	**Overlap**	**Identity**	**Positive**	**Gaps**
					**%**	**%**	**%**
**1**	Contig4149	$1.41e^{-39}$	350	285.3261	41.71429	61.71429	4.571429
**2**	PtaqEST_c54074	$3.23e^{-06}$	103	146.7391	31.63265	51.02041	8.163265
**3**	PtaqEST_s93201	0.000521	88	127.1739	28.04878	51.21951	4.878049
**4**	Contig5171	3.52346	60	79.8913	32.65306	46.93878	4.081633
**5**	PtaqEST_c12935	6.24495	57	35.86957	50	72.72727	0
**6**	Contig2423	8.93556	57	35.86957	50	72.72727	0
**7**	Contig21095	9.31704	57	44.02174	40.74074	55.55556	0

```
DtHNL1           -------MAGTGGGAEQFQLRGVLWGKAYSWKITGTTIDKVWSIVGDYVRVDNWVSSVVK   53
DtHNL2           -------MAGTRGGAEEFQLRGVLWGKAYSWKITGTTIDKVWSIVGDYVRVDNWVSSVVK   53
DtHNL3           -------MAGTGGGAEEFQLRGVLWGKAYSWKISGTTIDKVWAIVGDYVRVDNWVSSVVK   53
DtHNL4           -------MAGTGGGAEEFQLRGVLWGKAYSWKITGTTIDKVWSIVGDYVRVDNWVSSVVK   53
Contig56214      -----------------MGEGV-RVGDKGGRVGAGVAGNER------LCGIGQVGDHARA   36
Isotig02779      -----------------------------LWTL-AVTQNEVWEVTGDFLGVARWATSLVE   30
Isotig02778      MET------IQTAASRSYGEEEVLWGKAFKWEIKGAGEDEVWEVTGDFLGVARWATSLVE   54
Isotig02777      MET------IQTAASRSYGEEEVLWGKAFKWEIKGAGEDEVWEVTGDFLGVARWATSLVE   54
Isotig02776      MET------IQTAASRSYGEEEVLWGKAFKWEIKGAGEDEVWEVTGDFLGVARWATSLVE   54
Isotig02773      MET------IQTAASRSYGEEEVLWGKAFKWEIKGAGEDEVWEVTGDFLGVARWATSLVE   54
Isotig02774      MET------IQTAASRSYGEEEVLWGKAFKWEIKGAGEDEVWEVTGDFLGVARWATSLVE   54
Isotig02772      MET------IQTAASRSYGEEEVLWGKAFKWEIKGAGEDEVWEVTGDFLGVARWATSLVE   54
Isotig02775      METIQTATESMTAASRSYGEEEVLWGKAFKWEIKGVGEDEVWEVTGDFLGVARWATSLVE   60
Isotig02771      METIQTATESMTAASRSYGEEEVLWGKAFKWEIKGVGEDEVWEVTGDFLGVARWATSLVE   60
Isotig02770      METIQTATESMTAASRSYGEEEVLWGKAFKWEIKGVGEDEVWEVTGDFLGVARWATSLVE   60
Contig4149       METIQTATESMTAASRSYGEEEVLWGKAFKWEIKGVGEDEVWEVTGDFLGVARWATSLVE   60
                                                   ..  ::          : .

DtHNL1           SSHVVSGEANQ-TGCVRRFVCYPASEGESETVDYSELIHMNAAAHQYMYMIVGG-NITGF   111
DtHNL2           SSHVVSGDANQ-TGCVRRFVCYPASDGESETVDYSELIHMNAAAHQYMYMIVGG-NITGF   111
DtHNL3           SSHVVSGDANK-TGCVRRFVCYPASEGESETVDYSELIHMNAAAHQYMYMIVGG-NITGF   111
DtHNL4           SSHVVSGDANK-TGCVRRFVCYPASEGESETVDYSELIHMNAAAHQYMYMIVGG-NITGF   111
Contig56214      KLRAYRRRAPKSQVA*ESPFFTQRHPGSPSPFAFEKLLEMDEIHHHYTYTILSG-TLPGF   94
Isotig02779      SCELIEGEAHK-PGCVRRVLVYPQAPGEASTFALEKLLEMDALHHRYSYTILGGSTLPGF   89
Isotig02778      SCELIEGEAHK-PGCVRRVLVYPQAPGEASTFALEKLLEMDALHHRYSYTILGGSTLPGF   113
Isotig02777      SCELIEGEAHK-PGCVRRVLVYPQAPGEASTFALEKLLEMDALHHRYSYTILGGSTLPGF   113
Isotig02776      SCELIEGEAHK-PGCVRRVLVYPQAPGEASTFALEKLLEMDALHHRYSYTILGGSTLPGF   113
Isotig02773      SCELIEGEAHK-PGCVRRVLVYPQAPGEASTFALEKLLEMDALHHRYSYTILGGSTLPGF   113
Isotig02774      SCELIEGEAHK-PGCVRRVLVYPQAPGEASTFALEKLLEMDALHHRYSYTILGGSTLPGF   113
Isotig02772      SCELIEGEAHK-PGCVRRVLVYPQAPGEASTFALEKLLEMDALHHRYSYTILGGSTLPGF   113
Isotig02775      SCELIEGEAHK-PGCVRRVLVYPQAPGEASTFALEKLLEMDALHHRYSYTILGGSTLPGF   119
Isotig02771      SCELIEGEAHK-PGCVRRVLVYPQAPGEASTFALEKLLEMDALHHRYSYTILGGSTLPGF   119
Isotig02770      SCELIEGEAHK-PGCVRRVLVYPQAPGEASTFALEKLLEMDALHHRYSYTILGGSTLPGF   119
Contig4149       SCELIEGEAHK-PGCVRRVLVYPQAPGEASTFALEKLLEMDALHHHYSYTILGGSTLPGF   119
                 . .     * :   . .  .      *. . .  .:*:.*:   *:* * *:.* .: **

DtHNL1           SLMKNYVSNISLSSLPE-------EDGGGVIFYWSFTAEPASNLTEQKCIEIVFPLYTTA   164
DtHNL2           SLMKNYVSNISLSSLPE-------EDGGGVIFYWSFTAEPASNLTEQKCIEIVFPLYTTA   164
DtHNL3           SLMKNYVSNISLNSLPE-------ADGGGVILHWSFTAEPASNLTEQKCIEIVFPLYTTA   164
DtHNL4           SLMKNYVSNISLNSLPE-------ADGGGVIFHWSFTAEPASNLTEQKCIEIVFPLYTTA   164
Contig56214      SLMRDYISTFKLLPLPK--DDTKEGEDKGTLLNWSFVCSPVPTLSKEQTHTIAFSLYKAA   152
Isotig02779      SLMQDYVSTFKLSSLRLVYPSAEIDQENGTLLHWSFVCRPVSTLSEEETHNIAFSLYQAA   149
Isotig02778      SLMQDYVSTFKLSSLRLVYPSAEIDQENGTLLHWSFVCRPVSTLSEEETHNIAFSLYQAA   173
Isotig02777      SLMQDYVSTFKLSSLRLVYPSAEIDQENGTLLHWSFVCRPVSTLSEEETHNIAFSLYQAA   173
Isotig02776      SLMQDYVSTFKLSSLRLVYPSAEIDQENGTLLHWSFVCRPVSTLSEEETHNIAFSLYQAA   173
Isotig02773      SLMQDYVSTFKLSSLRLVYPSAEIDQENGTLLHWSFVCRPVSTLSEEETHNIAFSLYQAA   173
Isotig02774      SLMQDYVSTFKLSSLRLVYPSAEIDQENGTLLHWSFVCRPVSTLSEEETHNIAFSLYQAA   173
Isotig02772      SLMQDYVSTFKLSSLRLVYPSAEIDQENGTLLHWSFVCRPVSTLSEEETHNIAFSLYQAA   173
Isotig02775      SLMQDYVSTFKLSSLRLVYPSAEIDQENGTLLHWSFVCRPVSTLSEEETHNIAFSLYQAA   179
Isotig02771      SLMQDYVSTFKLSSLRLVYPSAEIDQENGTLLHWSFVCRPVSTLSEEETHNIAFSLYQAA   179
Isotig02770      SLMQDYVSTFKLSSLRLVYPSAEIDQENGTLLHWSFVCRPVSTLSEEETHNIAFSLYQAA   179
Contig4149       SLMQDYVSTFKLSSLRLVYPSAEIDQENGTLLHWSFVCRPVSTLSEEETHNIAFSLYQAA   179
                 ***::*:*.:.*  *          :  *.:: ***.. *. .*::::   *.* ** :*

DtHNL1           LKDLCTHLSIPESSVTLLDD--    184
DtHNL2           LKDLCTHLSIPESSVTLLDD--    184
DtHNL3           LKDLCTHLSIPESSVTLLGD--    184
DtHNL4           LKDLCTHLSIPESSVTLLGD--    184
Contig56214      VNDLKTYLSLSDDNITLISEAS    174
Isotig02779      VNDLKARLSLSDDRITLIP---    168
Isotig02778      VNDLKARLSLSDDRITLIP---    192
Isotig02777      VNDLKARLSLSDDRITLIP---    192
Isotig02776      VNDLKARLSLSDDRITLIP---    192
Isotig02773      VNDLKARLSLSDDRITLIP---    192
Isotig02774      VNDLKARLSLSDDRITLIP---    192
Isotig02772      VNDLKARLSLSDDRITLIP---    192
Isotig02775      VNDLKARLSLSDDRITLIP---    198
Isotig02771      VNDLKARLSLSDDRITLIP---    198
Isotig02770      VNDLKARLSLSDDRITLIP---    198
Contig4149       VNDLKARLSLSDDRITLIS---    198
                 ::** : **: :. :**:
```

Supplementary Figure 9a. Multiple sequence alignment of *Dt*HNLs and putative *Pta*HNLs.

Color code: stop codon (red); catalytic residues (green); important residues for HNL activity (cyan).

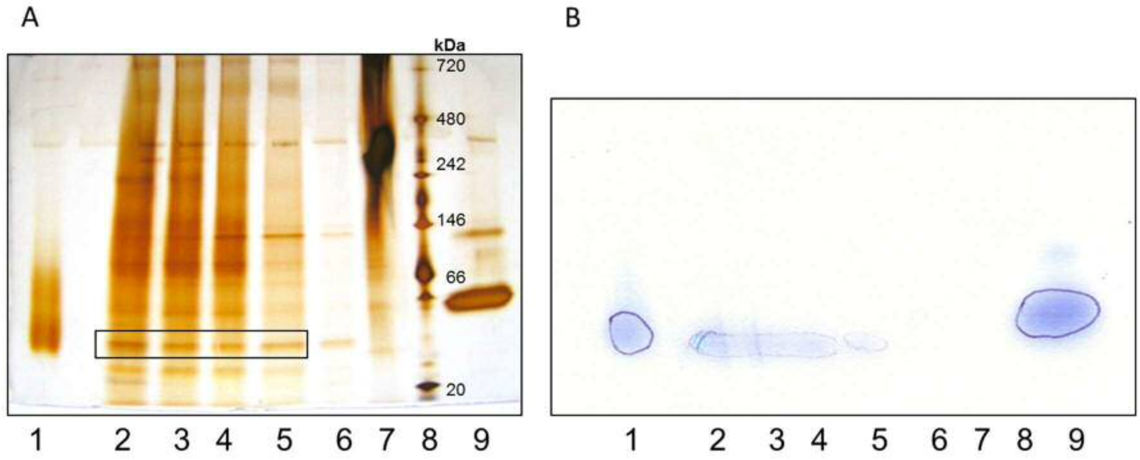

Supplementary Figure 9b. BLUE NATIVE PAGE followed by HNL activity assay.

In order to identify the HNL from *P. aquilinum*, the protein preparation from disrupted leaves was subjected to an anion exchange chromatography as described in Online Method. Elution fractions were concentrated and applied on a BN PAGE and then assayed for HNL activity.[5] A weak signal appears after 10 minutes at similar height as His-tagged *Dt*HNL1 dimer (about 46 kDa). The result is different from the one previously obtained for *Dt*HNL (activity at 20 kDa). The corresponding bands were analyzed by mass spectrometry and obtained peptides were matched against the *P. aquilinum* transcriptome. A list of isotigs is reported in the Appendix, Supplementary Dataset 2. None of the sequences found by blast were among the hits.

A: Different elution fractions after anion exchange purification and protein concentration were applied separately on BN PAGE (lane 2-6); flow through (7); positive control: purified *Dt*HNL1 (1); positive control *Pa*HNL (9); NativeMark^{TM} Unstained Protein Standard (Thermo Fisher Scientific) (8). B: HNL activity is depicted by the blue spot in correspondence to the different purification fractions and the two positive controls *Dt*HNL (1) and *Pa*HNL (9).

Supplementary Result 10. *Dt*HNL: a unique sequence within Bet v 1 superfamily

The Bet v 1 protein superfamily is characterized by small acidic proteins moderately conserved in their tertiary structure, but definitely diverse at sequence level.[6] *Dt*HNL belongs to polyketide_cyc2 pf10604 protein family in release 28.0 of the pfam database.[7]

Herein, *Dt*HNLs were compared with other members of Bet v 1 superfamily, in order to find other similar proteins with HNL activity. *Dt*HNL was subjected to blastp search (**Supplementary Fig. 10a**). Several unknown proteins with low similarity and sequence coverage were obtained. The sequence alignment between *Dt*HNL1 and the closest protein found (XP_009405224) is reported (**Supplementary Fig. 10b**). The six residues important for HNL activity are marked, and Arg and two Tyr are conserved only. Furthermore, a Glu is present instead of Ser, which is unlikely compatible with HNL activity (**Results**). The closest related characterized protein is the lachrymatory factor synthase (LFS) from *Allium cepa* (Uniprot P59082).[8] The two superimposed structures are visualized in **Supplementary Fig. 10e**. The architecture is similar, however sequence identity is less than 30% and catalytic and binding residues are different (**Supplementary Fig. 10d**). Second representatives of the protein family are phytohormone abscisic acid (ABA) receptors. They are characterized proteins and their tertiary structured is solved.[9] They play a role in different biological functions including plant defense response from pathogen attack.[10] Again, important binding residues or *Dt*HNL are not conserved in *At*PYL (Q8VZS8).

Structural comparison among the Bet v 1 superfamily and *Dt*HNL is limited, due the low number of protein structures which belongs to this superfamily deposited in the PDB. 3DM database overcomes this limitation,[11] therefore, a specific 3DM database for the Bet v 1 superfamily was developed. Specifically, the database included 264 structures and 13,904 sequences (October 2014). The difference between *Dt*HNL and the other superfamily members was again remarkable. Even if each subfamily was constituted of proteins with very low sequence identity (up to 30%), *Dt*HNLs resulted in a new subfamily named 3NEWA (**Supplementary Fig. 10f**). The 3D numbers indicate the position of a

specific residue within the protein structure, and key residues and their 3D number are reported in supplementary Table 11. A sequences subset was created with the following requirements: Tyr at 3D positions 79 and 91 and a basic residue, Arg or Lys, at 3D positon 50. According to the described parameters, a subset of 491 sequences was obtained, corresponding to 3.5% of the entire database. Finally, the amino acid occurrence was investigated at 3D positions 63 and 65 within the created subset (Supplementary Fig.10g). Only *Dt*HNL isoenzymes show the desired residues Asp and Ser. Glu was relatively conserved at the 3D position 65, but unlikely compatible with HNL activity (Results). Based on these results and todays knowledge, it can be hypothesized that *Dt*HNL is a unique enzyme within the protein superfamily.

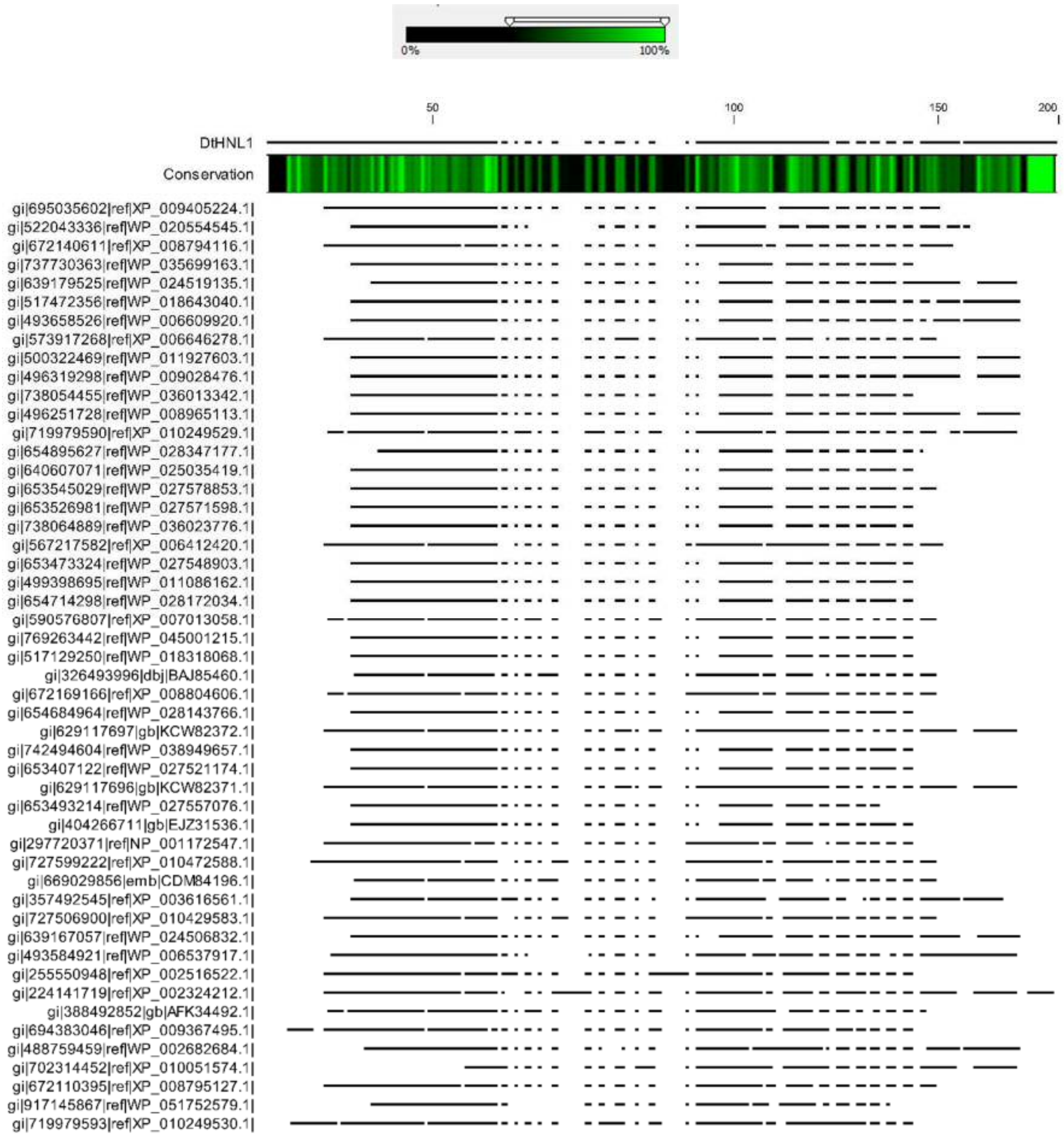

Supplementary Figure 10a. blastp of *Dt*HNL1 in NCBI.

A blastp search was performed with CLC Main Workbench 7.6.2 (QIAGEN Aarhus A/S), with the default parameters reported by the software (Protein matrix and gap costs: BLOSUM62 existence 11 extension 1; expectation value: 10.0; word size: 3; mask lower case: no; filter low complexity: yes; maximum number of hits: 50; limit by entry query: all organisms; database: nr).

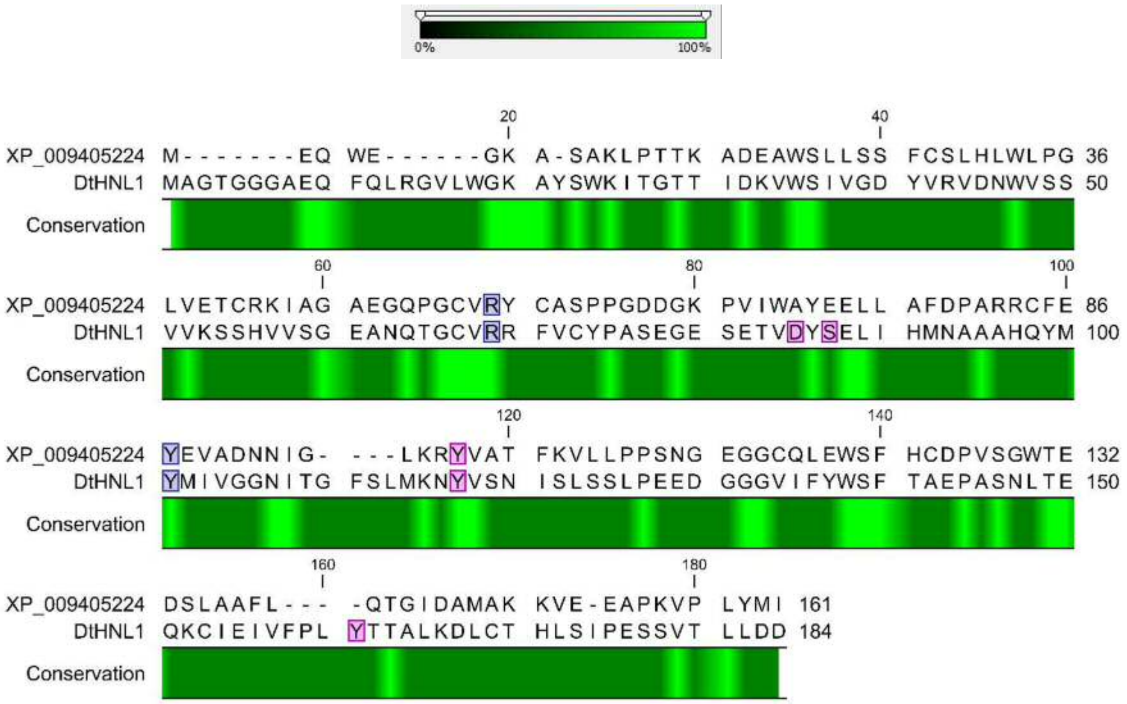

Supplementary Figure 10b. Sequence alignment of *Dt*HNL1 and XP_009405224.

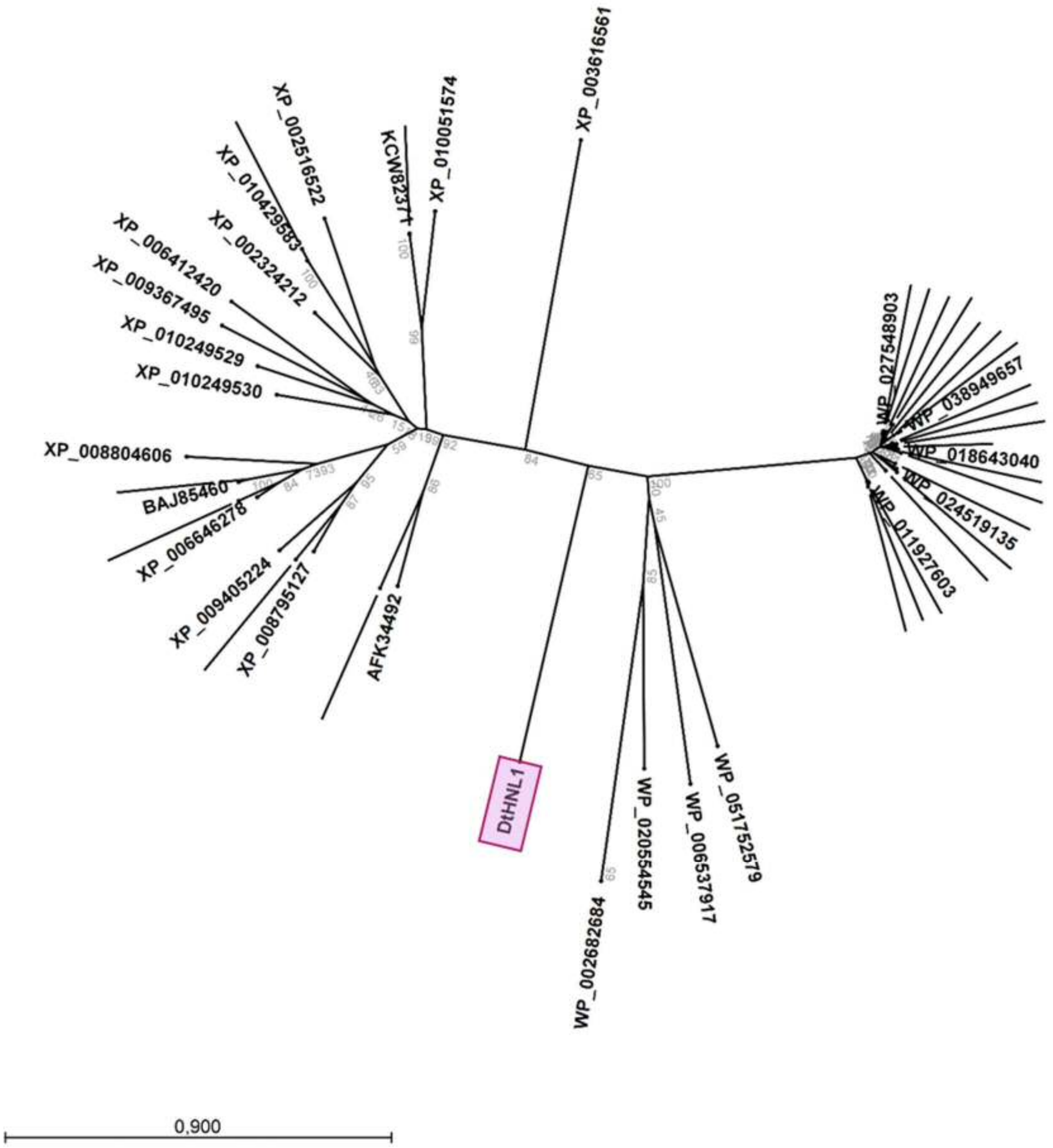

Supplementary Figure 10c. Phylogenetic tree.

Sequences of thirty members of pf10604 are confirmed at the protein level and they are deposited in the UniProtKB database[12] (Complete protein list Appendix, Supplementary Dataset 3). Most are non characterized proteins. A phylogenetic tree of the thirty sequences and *Dt*HNL1 is depicted. three main clusters can be distinguished, however *Dt*HNL does not belong to any of them.

The phylogenetic tree was built with CLC Main Workbench 7.6.2 (QIAGEN Aarhus A/S), with the default parameters reported by the software (Algorithm: neighbor Joining; distance measure: Jukes-Cantor; bootstrap: 100 replicates).

```
DtHNL1      MAGTGGGAEQFQ------------------------------------LRGVLWGKAY---SWKITGTTIDKVWSIVGDY 41

P59082      MELNPGAPAVVADSANGARK--------------------------------WSGKVH---ALLP-NTKPEQAWTLLKDF 44
Q8VZS8      MANSESSSSPVNEEENSQRISTLHHQTMPSDLTQDEFTQLSQSIAEFHTYQLGNGRCSSLLAQRI-HAPPETVWSVVRRF 79
            *  . ...  .                                           *:     :     :  : .*:::  :

DtHNL1      VRVDNWVSSVVKSSHVVSGEANQTGCVRRFVCYPA-SEGESETVDYSELIHMNAAAHQYMYMIVGGNITGFSLMKNYVSN 120
P59082      INLHKVM-PSLSVCELVEGEANVVGCVRYV-KGIM-HPIEEEFWAKEKLVALDNKNMSYSYIFTE----CFTGYEDYTAT 117
Q8VZS8      DRPQIYK-HFIKSCNVSEDFEMRVGCTRDV-NVISGLPAN---TSRERLDLLDDDRRVTGFSITG----GEHRLRNYKSV 150
             . .      :. ..: ..    .**.* .         :      ..*  ::       : :.          .:* :

DtHNL1      ISLSSLPEEDGGGVIFYWSFTAE-PASNLTEQKCIEIVFPLYTT----ALKDLCTHLSIPESSVTLLDD------- 184
P59082      MQIVEGPEHKGS--RFDWSFQCK-YIEGMTESAFTEILQHWATE-IGQKIEEVC------S--------------A 169
Q8VZS8      TTVHRFEKEEEEERI--WTVVLESYVVDVPEGNSEEDTRLFADTVIRLNLQKLA---SITEAMNRNNNNNNSSQVR 221
              :    :..       *:.  :    .:.*    *             ::.:.      .
```

Supplementary Figure 10d. Multiple sequence alignment of *Dt*HNL1, lachrymatory factor synthase and abscisic acid receptor.

Important residues for substrate binding and activity are highlighted: *Dt*HNL1 (red); *Ac*LFS (cyan);[13] *At*PYL1 (green).[9] Multiple sequence alignment was performed with Clustal Omega.[3]

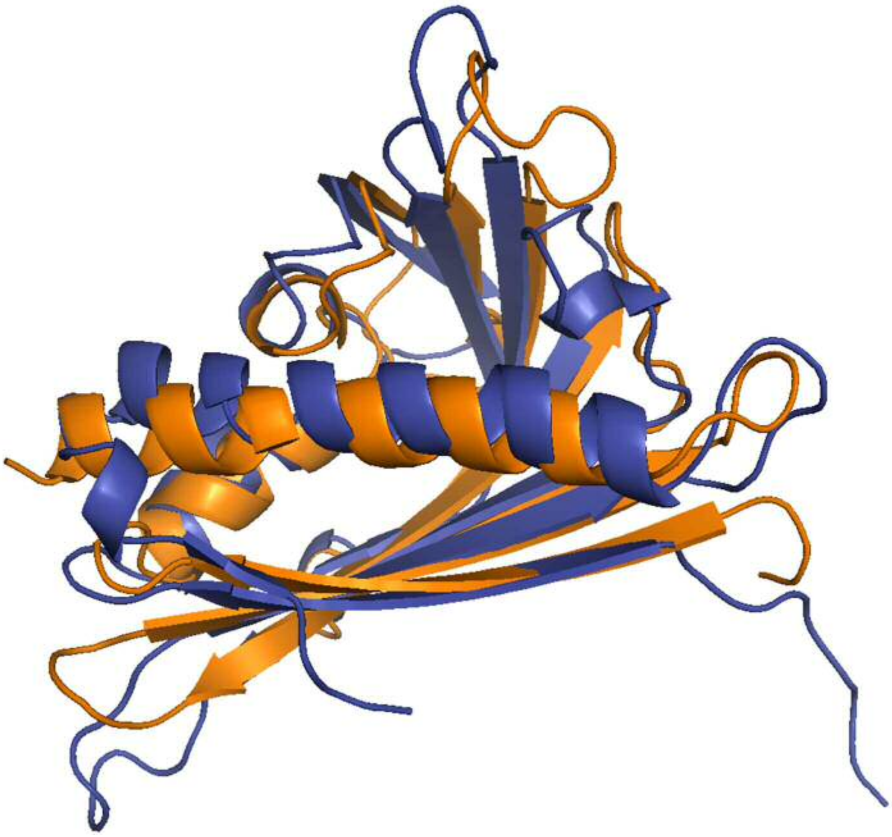

Supplementary Figure 10e. Superimposed structures of *Ac*LFS and *Dt*HNL1.

The *Ac*LFS structure was determined by homology modeling (Phyre2[14]).

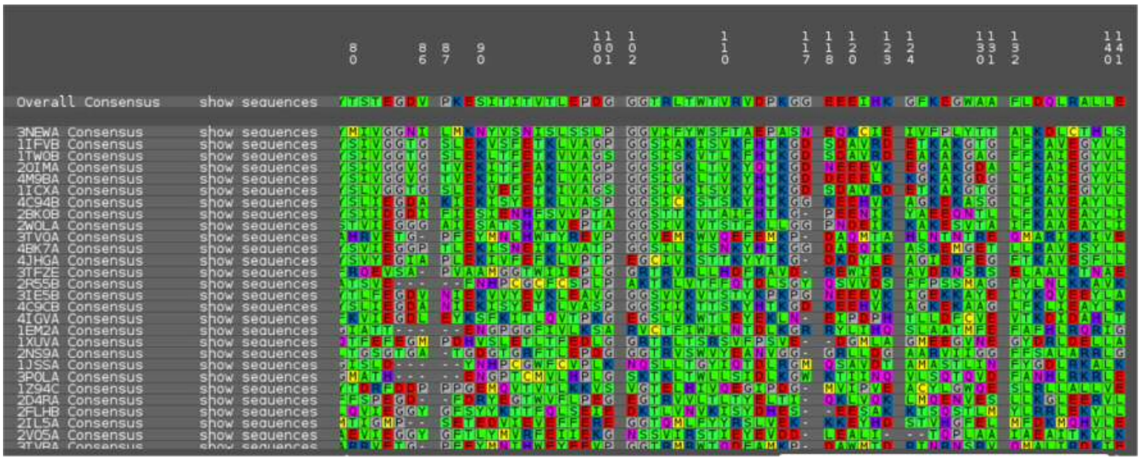

Supplementary Figure 10f. Snapshot of 3DM[11] of the Bet v 1 protein superfamily.

Part of the consensus alignment is represented. Specifically, the database includes 264 structures and 13,904 sequences. Subfamily 3NEWA includes *Dt*HNLs only. *Dt*HNL1 is the top entry (3NEWA).

Supplementary Table 10. 3D Number of *Dt*HNL relevant residues.

***Dt*HNL1 Residue**	**3D Number**
R69	50
D85	63
S87	65
Y101	79
Y117	91
Y161	129

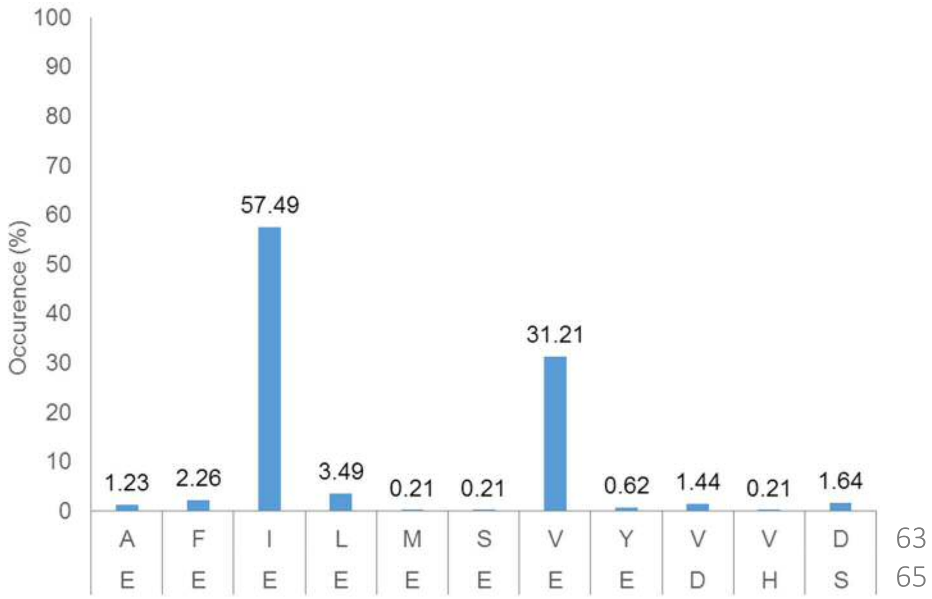

Supplementary Figure 10g. Residue occurrence in 3D positions 63 and 65.

The amino acid occurrence at 3D positions 63 and 65 within a created subset from Bet v 1 3DM database. Only the four *Dt*HNL isoenzymes show the desired residues at the two positions (1.64% occurrence). Subset parameters: Tyr at 3D positions 79 and 91, and a basic residue (Arg or Lys) at position 50. The subset consists of 491 sequences, 3.5% of the total database.

Supplementary Result 11. Appendix

Supplementary Table 11. Protein sequence list

Entry	Sequence
**1**	>*Dt*Isotig06604<br>MADSARTTVLVTGAGGRTGHIVYEKLKHKADKFHVRGLVRSEPSKAKIGGGEDVYIGDITKAESLGPAFAGVDVLII<br>LTSAAPQMKPGFDPSKGGRPEFYYEEGAYPEQVDWIGQKNQIDAAKEAGVKHIILVGSMGGTNPNHPLNSFGNGKIL<br>IWKRKSEQYLADSDTTYTLIRPGGLLDKEGGLRELLIGNNDELLATDTKTVPRADVAEVCVQAIVHDAVKNKAFDLT<br>SKPEGEGTPTTDFKILFSQVTASF
**2**	>*Dt*Isotig07200<br>MSYWKSKVVPKIKKFFDKGKKKGAAEFSKNFDSSKESLDKEIGEKSSDLSPKVVEIYRSSSTFIAKKLLKEPNEATV<br>KENSDATQGVLQELATAGFPGAQGIADAGKKYGPALLPGPVVYLFEKASVFLAEEPLPEEPKAETREVSAEDVKPAE<br>APATTSETPPPPPVADVPPPAVVEEEKKEAEPIVAAPPPEAAPPAAVEIPTSVDPTPPPPAPPADKPE
**3**	>*Dt*Isotig04065<br>MAKVHIMLLCTLCALSLSSLSAPQPALAASGPDHLDFYMYIAVQNNSNLDNPNVTFTAVQSAQPLSTQPNSFGIIHT<br>FDNPLTSAADLNSTQLGHVQGWYGDVGQNLLTLFLAQTFTWNDGTYNGTFSLLGVDVASDAVKFAPIVGGTGDFAYV<br>RGVAQQSLVSTATVNMETVSWFFYAIDFVY
**4**	>*Dt*Isotig04379<br>MGTSTWVVWSILLLAVAQVAGSIPIPRRYDGFVFNASSSSSPVLLEAFFDPLCPDSADAWPVVKKIAQYFQDDLLLI<br>VHPFPLPYHHNAYFASRALHIINNLNSSLTYPLLELFFENQDSFSTSETLAEAPSSVVDRIVQLAADSLNELVSSDF<br>ESQFKAGFSDTGTDLITRVSFKFGCSRVVVGTPYFFVNGIPLYDADSAWTFSEWAEIIEPLTGAQKIALA
**5**	>*Dt*Contig00505<br>MPFAQSLIVLFLAASALSYGGVLATTITAVNNCGTSGPLEFTGTSANGMNLAPAQSSGPIGVPDGWSGRVSLDPSPS<br>TLAEFSIVQNNKNTMDISLVDGFNVALGISYTGGNCIRNGEAAASNVACHISIDQCPASYRQGDRCVNPNKDAQTDY<br>SATVKGICPDAYSWSKDDATSTFTCDVGGDFTVTFCPP
**6**	>*Dt*HNL1<br>MAGTGGGAEQFQLRGVLWGKAYSWKITGTTIDKVWSIVGDYVRVDNWVSSVVKSSHVVSGEANQTGCVRRFVCYPAS<br>EGESETVDYSELIHMNAAAHQYMYMIVGGNITGFSLMKNYVSNISLSSLPEEDGGGVIFYWSFTAEPASNLTEQKCI<br>EIVFPLYTTALKDLCTHLSIPESSVTLLDD
**7**	>*Dt*HNL2<br>MAGTRGGAEEFQLRGVLWGKAYSWKITGTTIDKVWSIVGDYVRVDNWVSSVVKSSHVVSGDANQTGCVRRFVCYPAS<br>DGESETVDYSELIHMNAAAHQYMYMIVGGNITGFSLMKNYVSNISLSSLPEEDGGGVIFYWSFTAEPASNLTEQKCI<br>EIVFPLYTTALKDLCTHLSIPESSVTLLDD
**8**	>*Dt*HNL3<br>MAGTGGGAEEFQLRGVLWGKAYSWKISGTTIDKVWAIVGDYVRVDNWVSSVVKSSHVVSGDANKTGCVRRFVCYPAS<br>EGESETVDYSELIHMNAAAHQYMYMIVGGNITGFSLMKNYVSNISLNSLPEADGGGVILHWSFTAEPASNLTEQKCI<br>EIVFPLYTTALKDLCTHLSIPESSVTLLGD
**9**	>*Dt*HNL4<br>MAGTGGGAEEFQLRGVLWGKAYSWKITGTTIDKVWSIVGDYVRVDNWVSSVVKSSHVVSGDANKTGCVRRFVCYPAS<br>EGESETVDYSELIHMNAAAHQYMYMIVGGNITGFSLMKNYVSNISLNSLPEADGGGVIFHWSFTAEPASNLTEQKCI<br>EIVFPLYTTALKDLCTHLSIPESSVTLLGD
**10**	>*Pta*Isotig02775<br>METIQTATESMTAASRSYGEEEVLWGKAFKWEIKGVGEDEVWEVTGDFLGVARWATSLVESCELIEGEAHKPGCVRR<br>VLVYPQAPGEASTFALEKLLEMDALHHRYSYTILGGSTLPGFSLMQDYVSTFKLSSLRLVYPSAEIDQENGTLLHWS<br>FVCRPVSTLSEEETHNIAFSLYQAAVNDLKARLSLSDDRITLIP
**11**	>*Dt*HNL1_R69A<br>MAGTGGGAEQFQLRGVLWGKAYSWKITGTTIDKVWSIVGDYVRVDNWVSSVVKSSHVVSGEANQTGCVARFVCYPAS<br>EGESETVDYSELIHMNAAAHQYMYMIVGGNITGFSLMKNYVSNISLSSLPEEDGGGVIFYWSFTAEPASNLTEQKCI<br>EIVFPLYTTALKDLCTHLSIPESSVTLLDD
**12**	>*Dt*HNL1_S87A<br>MAGTGGGAEQFQLRGVLWGKAYSWKITGTTIDKVWSIVGDYVRVDNWVSSVVKSSHVVSGEANQTGCVRRFVCYPAS<br>EGESETVDYAELIHMNAAAHQYMYMIVGGNITGFSLMKNYVSNISLSSLPEEDGGGVIFYWSFTAEPASNLTEQKCI<br>EIVFPLYTTALKDLCTHLSIPESSVTLLDD
**13**	>*Dt*HNL1_D85A<br>MAGTGGGAEQFQLRGVLWGKAYSWKITGTTIDKVWSIVGDYVRVDNWVSSVVKSSHVVSGEANQTGCVRRFVCYPAS<br>EGESETVAYSELIHMNAAAHQYMYMIVGGNITGFSLMKNYVSNISLSSLPEEDGGGVIFYWSFTAEPASNLTEQKCI<br>EIVFPLYTTALKDLCTHLSIPESSVTLLDD
**14**	>*Dt*HNL1_Y101A<br>MAGTGGGAEQFQLRGVLWGKAYSWKITGTTIDKVWSIVGDYVRVDNWVSSVVKSSHVVSGEANQTGCVRRFVCYPAS<br>EGESETVDYSELIHMNAAAHQYMAMIVGGNITGFSLMKNYVSNISLSSLPEEDGGGVIFYWSFTAEPASNLTEQKCI<br>EIVFPLYTTALKDLCTHLSIPESSVTLLDD

Entry	Sequence
**15**	>*Dt*HNL1_Y117A<br>MAGTGGGAEQFQLRGVLWGKAYSWKITGTTIDKVWSIVGDYVRVDNWVSSVVKSSHVVSGEANQTGCVRRFVCYPAS<br>EGESETVDYSELIHMNAAAHQYMYMIVGGNITGFSLMKNAVSNISLSSLPEEDGGGVIFYWSFTAEPASNLTEQKCI<br>EIVFPLYTTALKDLCTHLSIPESSVTLLDD
**16**	>*Dt*HNL1_Y161A<br>MAGTGGGAEQFQLRGVLWGKAYSWKITGTTIDKVWSIVGDYVRVDNWVSSVVKSSHVVSGEANQTGCVRRFVCYPAS<br>EGESETVDYSELIHMNAAAHQYMYMIVGGNITGFSLMKNYVSNISLSSLPEEDGGGVIFYWSFTAEPASNLTEQKCI<br>EIVFPLATTALKDLCTHLSIPESSVTLLDD
**17**	>*Pta*Isotig02775_A92S<br>METIQTATESMTAASRSYGEEEVLWGKAFKWEIKGVGEDEVWEVTGDFLGVARWATSLVESCELIEGEAHKPGCVRR<br>VLVYPQAPGEASTFSLEKLLEMDALHHRYSYTILGGSTLPGFSLMQDYVSTFKLSSLRLVYPSAEIDQENGTLLHWS<br>FVCRPVSTLSEEETHNIAFSLYQAAVNDLKARLSLSDDRITLIP
**18**	>*Pta*Isotig02775_A92D_E94S<br>METIQTATESMTAASRSYGEEEVLWGKAFKWEIKGVGEDEVWEVTGDFLGVARWATSLVESCELIEGEAHKPGCVRR<br>VLVYPQAPGEASTFDLSKLLEMDALHHRYSYTILGGSTLPGFSLMQDYVSTFKLSSLRLVYPSAEIDQENGTLLHWS<br>FVCRPVSTLSEEETHNIAFSLYQAAVNDLKARLSLSDDRITLIP

Supplementary Table 12. Genes Deposited to GenBank® Database

<table>
<tr><th>Entry</th><th>Accession Number</th><th>Description</th><th>Ref. Protein</th></tr>
<tr><td rowspan="3">1</td><td>KT804569</td><td>Davallia tyermannii
Hydroxnyitrile lyase Isoform 1</td><td>DtHNL1</td></tr>
<tr><th colspan="3">Sequence</th></tr>
<tr><td colspan="3">ATGGCGGGAACGGGAGGGGGCGCAGAACAGTTCCAGCTCCGGGGAGTGCTGTGGGGGAAAGCCTACTCTTGGAAGATA
ACCGGAACGACAATCGACAAGGTGTGGTCGATTGTGGGCGATTATGTGCGCGTCGACAACTGGGTCTCTTCCGTCGTG
AAGAGCTCGCACGTCGTGTCTGGCGAGGCCAACCAGACGGGGTGCGTGAGGAGGTTCGTCTGCTACCCAGCCTCCGAG
GGAGAGTCGGAGACTGTGGACTACTCGGAGCTCATCCACATGAACGCTGCCGCGCACCAGTACATGTACATGATCGTG
GGAGGTAACATCACTGGCTTCTCTCTCATGAAGAACTATGTGAGCAATATCTCGCTGTCTTCTCTTCCTGAGGAGGAC
GGTGGTGGTGTAATCTTTTACTGGAGCTTCACAGCCGAGCCTGCCTCTAACCTCACGGAACAAAAATGCATAGAAATT
GTGTTCCCTCTCTATACCACTGCCCTGAAGGATTTATGCACTCACCTTTCCATACCCGAAAGCTCTGTTACACTTCTC
GATGATTAA</td></tr>
<tr><th></th><th>Accession Number</th><th>Description</th><th>Ref. Protein</th></tr>
<tr><td rowspan="3">2</td><td>KT805919</td><td>Davallia tyermannii
Hydroxnyitrile lyase Isoform 2</td><td>DtHNL2</td></tr>
<tr><th colspan="3">Sequence</th></tr>
<tr><td colspan="3">ATGGCGGGAACGAGAGGAGGCGCTGAAGAGTTCCAGCTCCGGGGAGTGCTGTGGGGGAAAGCCTACTCTTGGAAGATA
ACGGGAACGACAATCGACAAGGTGTGGTCGATTGTGGGTGATTATGTGCGCGTCGACAACTGGGTCTCTTCCGTCGTG
AAGAGCTCGCACGTCGTGTCCGGCGATGCCAACCAGACGGGGTGCGTGAGGAGGTTCGTCTGCTACCCAGCCTCCGAT
GGAGAGTCGGAGACTGTGGACTACTCGGAGCTCATCCACATGAACGCCGCCGCTCACCAATACATGTACATGATTGTG
GGAGGTAACATCACTGGCTTCTCTCTCATGAAGAACTATGTGAGCAATATCTCGCTGTCTTCTCTTCCTGAGGAGGAC
GGTGGTGGTGTCATCTTTTACTGGAGCTTCACAGCCGAGCCTGCCTCTAACCTCACGGAACAAAAATGCATAGAAATT
GTGTTTCCTCTCTACACCACTGCCCTGAAGGATTTATGCACTCACCTTTCCATACCCGAAAGCTCTGTTACACTTCTC
GATGATTAA</td></tr>
<tr><th></th><th>Accession Number</th><th>Description</th><th>Ref. Protein</th></tr>
<tr><td rowspan="3">3</td><td>KT805920</td><td>Davallia tyermannii
Hydroxnyitrile lyase Isoform 3</td><td>DtHNL3</td></tr>
<tr><th colspan="3">Sequence</th></tr>
<tr><td colspan="3">ATGGCAGGAACGGGAGGGGGCGCAGAAGAGTTCCAGCTGCGGGGAGTGCTGTGGGGGAAAGCCTACTCGTGGAAGATA
TCGGGAACGACAATTGACAAGGTGTGGGCGATTGTGGGCGACTATGTGCGCGTCGACAACTGGGTCTCTTCTGTAGTG
AAGAGCTCGCACGTCGTGTCTGGCGACGCTAACAAGACGGGGTGCGTGAGGAGGTTCGTCTGCTACCCAGCCTCCGAG
GGAGAGTCGGAGACTGTGGACTACTCGGAGCTCATCCACATGAACGCGGCCGCGCACCAGTACATGTACATGATTGTG
GGAGGTAACATCACTGGCTTCTCTCTCATGAAGAACTATGTGAGCAATATATCGCTCAATTCTCTTCCTGAGGCGGAC
GGTGGTGGTGTCATCCTCCACTGGAGCTTCACAGCCGAGCCTGCTTCTAACCTCACCGAACAAAAATGTATAGAAATT
GTGTTCCCTCTCTATACCACTGCCTTGAAGGATTTATGCACTCACCTTTCTATTCCGGAAAGCTCTGTTACACTCCTC
GGTGATTAA</td></tr>
<tr><th></th><th>Accession Number</th><th>Description</th><th>Ref. Protein</th></tr>
<tr><td rowspan="3">4</td><td>KT805921</td><td>Davallia tyermannii
Hydroxnyitrile lyase Isoform 4</td><td>DtHNL4</td></tr>
<tr><th colspan="3">Sequence</th></tr>
<tr><td colspan="3">ATGGCAGGAACGGGAGGGGGCGCAGAAGAGTTCCAGCTGCGGGGAGTGCTGTGGGGGAAAGCCTACTCTTGGAAGATA
ACGGGAACGACAATCGACAAGGTGTGGTCGATTGTGGGCGACTATGTCCGCGTCGATAACTGGGTCTCTTCCGTAGTG
AAGAGCTCGCACGTCGTGTCTGGCGATGCGAACAAGACGGGGTGCGTGAGGAGGTTCGTCTGCTACCCAGCCTCCGAG
GGAGAGTCGGAGACTGTGGACTACTCGGAGCTCATCCACATGAATGCGGCCGCGCACCAGTACATGTACATGATCGTG
GGAGGTAACATCACTGGCTTCTCTCTCATGAAGAACTATGTGAGCAATATATCGCTCAATTCTCTTCCTGAGGCGGAC
GGAGGTGGTGTCATCTTCCACTGGAGCTTCACAGCCGAGCCTGCTTCTAACCTCACCGAACAAAAATGCATCGAAATT
GTGTTCCCTCTCTATACCACTGCCTTGAAGGATTTATGCACTCACCTTTCTATCCCGGAAAGCTCTGTTACACTCCTC
GGTGATTAA</td></tr>
<tr><th></th><th>Accession Number</th><th>Description</th><th>Ref. Protein</th></tr>
<tr><td rowspan="3">5</td><td>KT818577</td><td>Davallia tyermannii
Unknown Protein</td><td>DtIsotig07200</td></tr>
<tr><th colspan="3">Sequence</th></tr>
<tr><td colspan="3">ATGAGTTATTGGAAGAGCAAGGTTGTGCCCAAAATCAAGAAGTTTTTTGACAAGGGGAAGAAGAAAGGAGCTGCTGAG
TTCTCCAAAAACTTTGATTCGTCCAAGGAGTCTTTGGACAAGGAGATAGGAGAGAAGAGTTCAGATCTGAGCCCCAAG
GTTGTGGAGATATACAGATCCTCTTCCACCTTCATTGCCAAGAAGTTGCTGAAGGAACCCAATGAGGCAACAGTGAAG
GAAAATTCCGATGCAACGCAAGGCGTGCTTCAGGAGCTGGCAACAGCAGGCTTTCCTGGAGCGCAGGGCATCGCTGAT
GCAGGCAAAAAGTATGGACCGGCGCTTTTACCGGGGCCGGTCGTGTACTTGTTCGAGAAAGCATCCGTGTTTTTGGCG
GAGGAGCCTTTGCCAGAGGAGCCCAAGGCAGAGACTAGAGAGGTGAGTGCGGAGGACGTCAAGCCAGCAGAAGCGCCA
GCGACGACTTCGGAAACTCCGCCGCCGCCACCTGTAGCAGACGTGCCTCCCCCAGCTGTCGTCGAGGAAGAAAAGAAG
GAAGCAGAGCCCATTGTTGCTGCCCCGCCGCCCGAAGCTGCCCCCCCTGCAGCTGTTGAGATCCCCACCTCCGTTGAC
CCTACGCCCCCGCCTCCTGCTCCGCCCGCCGACAAACCTGAGTAG</td></tr>
</table>

<table>
<tr><th></th><th>Accession Number</th><th>Description</th><th>Ref. Protein</th></tr>
<tr><td rowspan="3">6</td><td>KT818578</td><td>Davallia tyermannii
Unknown Protein</td><td>DtIsotig06604</td></tr>
<tr><td colspan="3">Sequence</td></tr>
<tr><td colspan="3">ATGGCAGACTCTGCTCGCACAACCGTACTCGTAACTGGTGCTGGTGGAAGAACGGGGCACATTGTATATGAGAAGCTG
AAACATAAGGCAGACAAGTTTCATGTGAGAGGTCTTGTGAGGTCAGAGCCAAGCAAGGCAAAGATTGGAGGGGGTGAG
GATGTGTACATAGGTGACATAACAAAGGCAGAGAGCCTGGGCCCAGCATTTGCAGGGGTGGATGTGCTCATCATCCTC
ACAAGTGCTGCTCCTCAAATGAAGCCAGGGTTTGATCCAAGCAAAGGAGGGCGCCCCGAGTTCTACTACGAAGAAGGC
GCATATCCTGAGCAGGTTGATTGGATCGGGCAAAAGAATCAAATCGATGCAGCCAAAGAAGCGGGGGTGAAGCATATC
ATTTTGGTTGGCTCGATGGGTGGCACAAATCCGAATCATCCTCTGAACTCTTTCGGAAATGGGAAGATTTTGATTTGG
AAAAGGAAGTCGGAGCAGTATTTGGCAGACTCTGATACAACCTACACCTTAATCAGGCCGGGTGGGCTGCTAGACAAA
GAAGGTGGGTTGCGAGAGCTCTTGATTGGAAACAACGACGAACTTCTCGCCACAGATACCAAAACGGTTCCACGAGCA
GATGTTGCGGAAGTCTGTGTACAGGCAATTGTTCATGATGCAGTAAAGAATAAGGCTTTTGACTTGACCTCGAAACCG
GAGGGTGAAGGTACGCCGACGACAGATTTCAAGATTCTCTTCAGTCAAGTAACTGCAAGCTTTTAG</td></tr>
<tr><th></th><th>Accession Number</th><th>Description</th><th>Ref. Protein</th></tr>
<tr><td rowspan="3">7</td><td>KT818579</td><td>Davallia tyermannii
Unknown Protein</td><td>DtIsotig04379</td></tr>
<tr><td colspan="3">Sequence</td></tr>
<tr><td colspan="3">ATGGGCACCTCAACGTGGGTGGTATGGAGCATTCTGTTGCTAGCAGTGGCGCAAGTAGCGGGGAGCATCCCCATCCCA
AGACGCTACGATGGCTTCGTCTTCAATGCTTCTTCATCCTCGTCGCCTGTGTTGCTGGAGGCCTTCTTCGATCCCCTC
TGCCCGGATAGCGCAGACGCTTGGCCTGTTGTCAAGAAAATCGCCCAATACTTCCAGGACGATCTGCTCCTCATTGTC
CACCCCTTCCCTCTCCCGTACCATCACAATGCATATTTTGCAAGTAGAGCATTGCACATCATCAATAACCTGAACAGT
TCTCTCACTTATCCATTGCTTGAGTTGTTTTTTGAAAACCAGGATAGCTTTTCAACGAGTGAAACGCTAGCGGAGGCA
CCATCCTCCGTCGTAGACAGAATCGTTCAACTGGCAGCAGATAGCTTGAATGAACTCGTGTCTTCCGATTTTGAGAGC
CAGTTCAAAGCAGGATTTTCTGATACAGGAACGGATCTGATCACTCGTGTTTCGTTCAAGTTTGGGTGCTCGCGCGTT
GTGGTCGGTACGCCGTACTTTTTTGTCAATGGCATACCTCTTTATGATCGGATTCGGCATGGACCTTCTCTGAATGGG
CAGAGATTATTGAGCCATTAACAGGGGCGCAAAAAATCGCGCTTGCATAA</td></tr>
<tr><th></th><th>Accession Number</th><th>Description</th><th>Ref. Protein</th></tr>
<tr><td rowspan="3">8</td><td>KT818580</td><td>Davallia tyermannii
Unknown Protein</td><td>DtIsotig04065</td></tr>
<tr><td colspan="3">Sequence</td></tr>
<tr><td colspan="3">ATGGCCAAGGTGCACATTATGCTCCTATGTACATTATGCGCTCTCTCCCTCTCCTCCCTCTCTGCCCCACAGCCAGCT
TTGGCTGCCTCGGGTCCTGACCATCTTGACTTCTACATGTACATTGCTGTTCAGAATAACAGCAATCTCGACAACCCC
AATGTCACCTTCACAGCCGTGCAGTCCGCGCAGCCGCTTTCGACGCAACCCAACTCATTCGGCATCATCCACACCTTC
GACAACCCACTCACCAGTGCCGCAGATCTCAACTCTACGCAGCTCGGGCACGTGCAGGGCTGGTATGGTGATGTGGGG
CAGAATCTTTTGACGTTGTTCCTGGCGCAGACCTTTACCTGGAACGACGGCACCTACAATGGCACATTCAGCCTGTTG
GGGGTGGACGTCGCGTCAGATGCGGTAAAGTTTGCACCCATTGTTGGCGGCACGGGCGACTTTGCGTATGTGCGCGGA
GTGGCTCAACAGTCCCTTGTCTCAACCGCTACTGTAAACATGGAAACGGTCTCATGGTTCTTTTATGCCATTGACTTT
GTATACTAG</td></tr>
<tr><th></th><th>Accession Number</th><th>Description</th><th>Ref. Protein</th></tr>
<tr><td rowspan="3">9</td><td>KT818581</td><td>Davallia tyermannii
Unknown Protein</td><td>DtContig00505</td></tr>
<tr><td colspan="3">Sequence</td></tr>
<tr><td colspan="3">ATGCCCTTTGCTCAATCCTTGATAGTGCTTTTCCTTGCGGCTTCAGCACTCAGCTATGGAGGAGTGTTGGCAACAACC
ATAACGGCTGTGAACAACTGTGGAACAAGCGGCCCACTTGAGTTCACAGGCACTAGTGCCAACGGCATGAACTTGGCA
CCTGCACAATCGTCTGGCCCCATCGGTGTACCTGACGGATGGTCGGGCCGAGTTTCGCTGGACCCTTCGCCGTCCACT
TTAGCAGAGTTCAGCATCGTCCAAAACAACAAGAATACCATGGATATTAGTCTGGTGGATGGCTTCAACGTTGCTCTG
GGAATCTCATACACCGGTGGTAATTGCATAAGGAATGGTGAAGCTGCAGCTAGCAACGTGGCATGCCACATTTCTATC
GACCAGTGTCCTGCAAGCTACAGACAAGGCGACCGATGCGTCAACCCTAACAAAGACGCCCAGACTGACTACTCCGCT
ACTGTGAAGGGGATATGTCCGGACGCCTATAGCTGGTCCAAGGATGATGCAACTAGCACGTTCACGTGCGATGTTGGT
GGTGACTTTACCGTCACATTCTGCCCTCCATGA</td></tr>
<tr><th></th><th>Accession Number</th><th>Description</th><th>Ref. Protein</th></tr>
<tr><td rowspan="3">10</td><td>KT818582</td><td>Pteridium aquilinum
Unknown Protein</td><td>PtaIsotig02775</td></tr>
<tr><td colspan="3">Sequence</td></tr>
<tr><td colspan="3">ATGGAGACGATTCAAACAGCGACGGAGTCGATGACAGCGGCTAGCAGGAGCTATGGAGAGGAGGAGGTATTATGGGGG
AAGGCGTTCAAGTGGGAGATAAAGGGTGTAGGGGAGGACGAGGTGTGGGAGGTAACCGGAGACTTTCTGGGAGTGGCC
AGGTGGGCAACCTCGCTGGTGGAGAGCTGTGAGCTTATAGAAGGAGAGGCCCATAAGCCAGGCTGCGTGAGAAGGGTC
CTTGTTTATCCCCAGGCTCCTGGGGAGGCCTCCACTTTTGCCCTTGAAAAGCTCTTAGAAATGGACGCGCTACACCAC
CGTTACTCTTACACTATCCTTGGCGGAAGCACCTTGCCTGGCTTCTCTCTCATGCAGGACTATGTCAGTACCTTCAAG
CTCTCTTCCCTACGCCTGGTGTACCCCTCTGCAGAAATTGACCAAGAAAATGGTACCCTCCTCCATTGGAGCTTTGTT
TGTCGCCCAGTCTCTACCTTGTCTGAGGAGGAAACCCACAACATTGCCTTCTCTCTCTACCAGGCTGCAGTCAACGAT
CTCAAAGCTCGCCTCTCCTTGTCTGACGACCGCATTACTCTCATCCCGTAA</td></tr>
</table>

Supplementary Table 13. Primer list

Entry	Primer Name	Sequence	Purpose
1	DtIsotig02643_fw	AGCTCCCTAGCAAGTCATG	amplification from gDNA
2	DtIsotig02641_fw	AGAGAGTGAGGCGAGGTAG	amplification from gDNA
3	DtIsotig02641/3_rev	GGAGGATGAAAAGCTTAATC	amplification from gDNA
3	DtIsotig07602_fw	TAGAAAATGTAATTAGGGGGGTGAG ATAAAG	amplification from gDNA
4	DtIsotig07602_rev	GAGAGTAAGGAGCAGTAGGCAAGC	amplification from gDNA
5	DtContig00751_fw	TATAATTAGGGAGGGGTGAGATAAA GC	amplification from gDNA
6	DtContig00751_rev	GTAAGGGGCAGTAGGCAAGC	amplification f rom gDNA
7	DtHNL1_Ec_NcoI_fw	AATGCCCATGGCAGGCACCGGTGGT G	Cloning pEHisTEV
8	DtHNL1_Ec_HindIII_rev	AATGCAAGCTTTTAATCATCCAGCA GGGTAACGC	Cloning pEHisTEV
9	DtHNL2_Dt_NcoI_fw	AATGCCCATGGCGGGAACGAGAGGA GGCG	Cloning pEHisTEV
10	DtHNL2_Dt_HindIII_rev	AATGCAAGCTTTTAATCATCGAGAA GTGTAACAGAGC	Cloning pEHisTEV
11	DtHNL3/4_Dt_NcoI_fw	AATGCCCATGGCAGGAACGGGAGGG GGC	Cloning pEHisTEV
12	DtHNL3/4_Dt_HindIII_rev	AATGCAAGCTTTTAATCACCGAGGA GTGTAACAG	Cloning pEHisTEV
13	PtaIsotig02775_Ec_NcoI_fw	AATGCCCATGGAAACCATTCAGACC	Cloning pEHisTEV
14	PtaIsotig02775_Ec_HindIII_rev	AATGCAAGCTTTTACGGAATCAGGG TAATAC	Cloning pEHisTEV
15	pEHisTEV_DtHNL1_gibson_fw	TACCCATCTGAGCATTCCGGAAAG	Gibson cloning pEHisTEV
16	pEHisTEV_DtHNL1_gibson_rev	CAGCTATATGCTTTACCCCACAGAA C	Gibson cloning pEHisTEV
17	pEHisTEV_PtaIsotig02775_gibson_fw	ATATTGCATTTAGCCTGTATCAGGC AG	Gibson cloning pEHisTEV
18	pEHisTEV_PtaIsotig02775_gibson_rev	AGGCTTTACCCCAAAGAACCTCTTC	Gibson cloning pEHisTEV
19	pEHisTEVseq1	CTTTAATAGTGGACTCTTGTTC	Sequencing
20	pEHisTEVseq2	GTTTATGCATTTCTTTCCAGAC	Sequencing
21	pEHisTEVseq3	GCAAGACGTTTCCCGTTGAATATG	Sequencing
22	pEHisTEVseq4	AGATACCTACAGCGTGAGCTATG	Sequencing
23	pEHisTEVseq5	GTGACTGGGTCATGGCTGCG	Sequencing
24	pEHisTEVseq6	TTCCACAGGGTAGCCAGCAGCATC	Sequencing
25	pEHisTEVseq7	TTGAAGGCTCTCAAGGGCATCG	Sequencing
26	pEHisTEVseq8	CGGCTGAATTTGATTGCGAGTG	Sequencing
27	pEHisTEVseq9	CACTTTTTCCCGCGTTTTCGCAG	Sequencing
28	pEHisTEVseq10	GGAATTGTGAGCGGATAACAATTC	Sequencing

Standard T7 forward and reverse primers provided by LGC Genomics or Microsynth AG were employed for the sequencing of CDS in pEHisTEV plasmid.

Supplementary Table 14. gBLock® Gene fragments list

Entry	Name	Sequence
**1**	DtHNL1_R69A	GTTCTGTGGGGTAAAGCATATAGCTGGAAAATTACCGGCACCACCATTGATAAAGTTTG<br>GAGCATTGTTGGTGATTATGTGCGTGTTGATAATTGGGTTAGCAGCGTTGTTAAAAGCA<br>GCCATGTTGTTAGCGGTGAAGCAAATCAGACCGGTTGTGTTGCACGTTTTGTTTGTTAT<br>CCGGCAAGCGAAGGTGAAAGCGAAACCGTTGATTATAGCGAACTGATTCACATGAATGC<br>AGCAGCACATCAGTATATGTATATGATTGTGGGTGGCAACATTACCGGTTTTAGCCTGA<br>TGAAAAACTACGTGAGCAATATTAGCCTGAGCAGCCTGCCGGAAGAGGATGGTGGTGGC<br>GTTATCTTTTATTGGAGCTTTACCGCAGAACCGGCAAGCAATCTGACCGAACAGAAATG<br>TATTGAAATTGTGTTTCCGCTGTATACCACCGCACTGAAAGACCTGTGTACCCATCTGA<br>GCATTCCGGAAAG
**2**	DtHNL1_D85A	GTTCTGTGGGGTAAAGCATATAGCTGGAAAATTACCGGCACCACCATTGATAAAGTTTG<br>GAGCATTGTTGGTGATTATGTGCGTGTTGATAATTGGGTTAGCAGCGTTGTTAAAAGCA<br>GCCATGTTGTTAGCGGTGAAGCAAATCAGACCGGTTGTGTTCGTCGTTTTGTTTGTTAT<br>CCGGCAAGCGAAGGTGAAAGCGAAACCGTTGCATATAGCGAACTGATTCACATGAATGC<br>AGCAGCACATCAGTATATGTATATGATTGTGGGTGGCAACATTACCGGTTTTAGCCTGA<br>TGAAAAACTACGTGAGCAATATTAGCCTGAGCAGCCTGCCGGAAGAGGATGGTGGTGGC<br>GTTATCTTTTATTGGAGCTTTACCGCAGAACCGGCAAGCAATCTGACCGAACAGAAATG<br>TATTGAAATTGTGTTTCCGCTGTATACCACCGCACTGAAAGACCTGTGTACCCATCTGA<br>GCATTCCGGAAAG
**3**	DtHNL1_S87A	GTTCTGTGGGGTAAAGCATATAGCTGGAAAATTACCGGCACCACCATTGATAAAGTTTG<br>GAGCATTGTTGGTGATTATGTGCGTGTTGATAATTGGGTTAGCAGCGTTGTTAAAAGCA<br>GCCATGTTGTTAGCGGTGAAGCAAATCAGACCGGTTGTGTTCGTCGTTTTGTTTGTTAT<br>CCGGCAAGCGAAGGTGAAAGCGAAACCGTTGATTATGCAGAACTGATTCACATGAATGC<br>AGCAGCACATCAGTATATGTATATGATTGTGGGTGGCAACATTACCGGTTTTAGCCTGA<br>TGAAAAACTACGTGAGCAATATTAGCCTGAGCAGCCTGCCGGAAGAGGATGGTGGTGGC<br>GTTATCTTTTATTGGAGCTTTACCGCAGAACCGGCAAGCAATCTGACCGAACAGAAATG<br>TATTGAAATTGTGTTTCCGCTGTATACCACCGCACTGAAAGACCTGTGTACCCATCTGA<br>GCATTCCGGAAAG
**4**	DtHNL1_Y101A	GTTCTGTGGGGTAAAGCATATAGCTGGAAAATTACCGGCACCACCATTGATAAAGTTTG<br>GAGCATTGTTGGTGATTATGTGCGTGTTGATAATTGGGTTAGCAGCGTTGTTAAAAGCA<br>GCCATGTTGTTAGCGGTGAAGCAAATCAGACCGGTTGTGTTCGTCGTTTTGTTTGTTAT<br>CCGGCAAGCGAAGGTGAAAGCGAAACCGTTGATTATAGCGAACTGATTCACATGAATGC<br>AGCAGCACATCAGTATATGGCAATGATTGTGGGTGGCAACATTACCGGTTTTAGCCTGA<br>TGAAAAACTACGTGAGCAATATTAGCCTGAGCAGCCTGCCGGAAGAGGATGGTGGTGGC<br>GTTATCTTTTATTGGAGCTTTACCGCAGAACCGGCAAGCAATCTGACCGAACAGAAATG<br>TATTGAAATTGTGTTTCCGCTGTATACCACCGCACTGAAAGACCTGTGTACCCATCTGA<br>GCATTCCGGAAAG
**5**	DtHNL1_Y117A	GTTCTGTGGGGTAAAGCATATAGCTGGAAAATTACCGGCACCACCATTGATAAAGTTTG<br>GAGCATTGTTGGTGATTATGTGCGTGTTGATAATTGGGTTAGCAGCGTTGTTAAAAGCA<br>GCCATGTTGTTAGCGGTGAAGCAAATCAGACCGGTTGTGTTCGTCGTTTTGTTTGTTAT<br>CCGGCAAGCGAAGGTGAAAGCGAAACCGTTGATTATAGCGAACTGATTCACATGAATGC<br>AGCAGCACATCAGTATATGTATATGATTGTGGGTGGCAACATTACCGGTTTTAGCCTGA<br>TGAAAAACGCAGTGAGCAATATTAGCCTGAGCAGCCTGCCGGAAGAGGATGGTGGTGGC<br>GTTATCTTTTATTGGAGCTTTACCGCAGAACCGGCAAGCAATCTGACCGAACAGAAATG<br>TATTGAAATTGTGTTTCCGCTGTATACCACCGCACTGAAAGACCTGTGTACCCATCTGA<br>GCATTCCGGAAAG
**6**	DtHNL1_Y161A	GTTCTGTGGGGTAAAGCATATAGCTGGAAAATTACCGGCACCACCATTGATAAAGTTTG<br>GAGCATTGTTGGTGATTATGTGCGTGTTGATAATTGGGTTAGCAGCGTTGTTAAAAGCA<br>GCCATGTTGTTAGCGGTGAAGCAAATCAGACCGGTTGTGTTCGTCGTTTTGTTTGTTAT<br>CCGGCAAGCGAAGGTGAAAGCGAAACCGTTGATTATAGCGAACTGATTCACATGAATGC<br>AGCAGCACATCAGTATATGTATATGATTGTGGGTGGCAACATTACCGGTTTTAGCCTGA<br>TGAAAAACTACGTGAGCAATATTAGCCTGAGCAGCCTGCCGGAAGAGGATGGTGGTGGC<br>GTTATCTTTTATTGGAGCTTTACCGCAGAACCGGCAAGCAATCTGACCGAACAGAAATG<br>TATTGAAATTGTGTTTCCGCTGGCAACCACCGCACTGAAAGACCTGTGTACCCATCTGA<br>GCATTCCGGAAAG
**7**	DtHNL1_Y101F	GTTCTGTGGGGTAAAGCATATAGCTGGAAAATTACCGGCACCACCATTGATAAAGTTTG<br>GAGCATTGTTGGTGATTATGTGCGTGTTGATAATTGGGTTAGCAGCGTTGTTAAAAGCA<br>GCCATGTTGTTAGCGGTGAAGCAAATCAGACCGGTTGTGTTCGTCGTTTTGTTTGTTAT<br>CCGGCAAGCGAAGGTGAAAGCGAAACCGTTGATTATAGCGAACTGATTCACATGAATGC<br>AGCAGCACATCAGTATATGTTTATGATTGTGGGTGGCAACATTACCGGTTTTAGCCTGA<br>TGAAAAACTACGTGAGCAATATTAGCCTGAGCAGCCTGCCGGAAGAGGATGGTGGTGGC<br>GTTATCTTTTATTGGAGCTTTACCGCAGAACCGGCAAGCAATCTGACCGAACAGAAATG<br>TATTGAAATTGTGTTTCCGCTGTATACCACCGCACTGAAAGACCTGTGTACCCATCTGA<br>GCATTCCGGAAAG

Entry	Name	Sequence
**8**	DtHNL1_Y117F	GTTCTGTGGGGTAAAGCATATAGCTGGAAAATTACCGGCACCACCATTGATAAAGTTTG<br>GAGCATTGTTGGTGATTATGTGCGTGTTGATAATTGGGTTAGCAGCGTTGTTAAAAGCA<br>GCCATGTTGTTAGCGGTGAAGCAAATCAGACCGGTTGTGTTCGTCGTTTTGTTTGTTAT<br>CCGGCAAGCGAAGGTGAAAGCGAAACCGTTGATTATAGCGAACTGATTCACATGAATGC<br>AGCAGCACATCAGTATATGTATATGATTGTGGGTGGCAACATTACCGGTTTTAGCCTGA<br>TGAAAAACTTTGTGAGCAATATTAGCCTGAGCAGCCTGCCGGAAGAGGATGGTGGTGGC<br>GTTATCTTTTATTGGAGCTTTACCGCAGAACCGGCAAGCAATCTGACCGAACAGAAATG<br>TATTGAAATTGTGTTTCCGCTGTATACCACCGCACTGAAAGACCTGTGTACCCATCTGA<br>GCATTCCGGAAAG
**9**	DtHNL1_Y161F	GTTCTGTGGGGTAAAGCATATAGCTGGAAAATTACCGGCACCACCATTGATAAAGTTTG<br>GAGCATTGTTGGTGATTATGTGCGTGTTGATAATTGGGTTAGCAGCGTTGTTAAAAGCA<br>GCCATGTTGTTAGCGGTGAAGCAAATCAGACCGGTTGTGTTCGTCGTTTTGTTTGTTAT<br>CCGGCAAGCGAAGGTGAAAGCGAAACCGTTGATTATAGCGAACTGATTCACATGAATGC<br>AGCAGCACATCAGTATATGTATATGATTGTGGGTGGCAACATTACCGGTTTTAGCCTGA<br>TGAAAAACTACGTGAGCAATATTAGCCTGAGCAGCCTGCCGGAAGAGGATGGTGGTGGC<br>GTTATCTTTTATTGGAGCTTTACCGCAGAACCGGCAAGCAATCTGACCGAACAGAAATG<br>TATTGAAATTGTGTTTCCGCTGTTTACCACCGCACTGAAAGACCTGTGTACCCATCTGA<br>GCATTCCGGAAAG
**10**	DtHNL1_<br>D85S_S87D	GTTCTGTGGGGTAAAGCATATAGCTGGAAAATTACCGGCACCACCATTGATAAAGTTTG<br>GAGCATTGTTGGTGATTATGTGCGTGTTGATAATTGGGTTAGCAGCGTTGTTAAAAGCA<br>GCCATGTTGTTAGCGGTGAAGCAAATCAGACCGGTTGTGTTCGTCGTTTTGTTTGTTAT<br>CCGGCAAGCGAAGGTGAAAGCGAAACCGTTAGCTATGATGAACTGATTCACATGAATGC<br>AGCAGCACATCAGTATATGTATATGATTGTGGGTGGCAACATTACCGGTTTTAGCCTGA<br>TGAAAAACTACGTGAGCAATATTAGCCTGAGCAGCCTGCCGGAAGAGGATGGTGGTGGC<br>GTTATCTTTTATTGGAGCTTTACCGCAGAACCGGCAAGCAATCTGACCGAACAGAAATG<br>TATTGAAATTGTGTTTCCGCTGTATACCACCGCACTGAAAGACCTGTGTACCCATCTGA<br>GCATTCCGGAAAG
**11**	DtHNL1_R69K	GTTCTGTGGGGTAAAGCATATAGCTGGAAAATTACCGGCACCACCATTGATAAAGTTTG<br>GAGCATTGTTGGTGATTATGTGCGTGTTGATAATTGGGTTAGCAGCGTTGTTAAAAGCA<br>GCCATGTTGTTAGCGGTGAAGCAAATCAGACCGGTTGTGTTAAACGTTTTGTTTGTTAT<br>CCGGCAAGCGAAGGTGAAAGCGAAACCGTTGATTATAGCGAACTGATTCACATGAATGC<br>AGCAGCACATCAGTATATGTATATGATTGTGGGTGGCAACATTACCGGTTTTAGCCTGA<br>TGAAAAACTACGTGAGCAATATTAGCCTGAGCAGCCTGCCGGAAGAGGATGGTGGTGGC<br>GTTATCTTTTATTGGAGCTTTACCGCAGAACCGGCAAGCAATCTGACCGAACAGAAATG<br>TATTGAAATTGTGTTTCCGCTGTATACCACCGCACTGAAAGACCTGTGTACCCATCTGA<br>GCATTCCGGAAAG
**12**	Ptaiso02775_<br>A92S	AGCAAGCCGTAGCTATGGTGAAGAAGAGGTTCTTTGGGGTAAAGCCTTTAAATGGGAAA<br>TTAAAGGTGTGGGCGAAGATGAAGTTTGGGAAGTTACCGGTGATTTTCTGGGTGTTGCA<br>CGTTGGGCAACCAGCCTGGTGGAAAGCTGTGAACTGATTGAAGGTGAAGCACATAAACC<br>GGGTTGTGTTCGTCGTGTTCTGGTTTATCCGCAGGCACCGGGTGAAGCAAGCACCTTTA<br>GCCTGGAAAAACTGCTGGAAATGGATGCACTGCATCATCGTTATAGTTATACCATTCTG<br>GGTGGTAGCACCCTGCCTGGTTTTAGCCTGATGCAGGATTATGTTAGCACCTTTAAACT<br>GAGCAGCCTGCGTCTGGTGTATCCGAGCGCAGAAATTGATCAAGAAAATGGCACCCTGC<br>TGCATTGGAGCTTTGTTTGTCGTCCGGTGAGCACCCTGAGCGAAGAAGAAACCCATAAT<br>ATTGCATTTAGCCTGTATCAGGCAGCCG
**13**	Ptaiso02775_<br>A92D_E94S	AGCAAGCCGTAGCTATGGTGAAGAAGAGGTTCTTTGGGGTAAAGCCTTTAAATGGGAAA<br>TTAAAGGTGTGGGCGAAGATGAAGTTTGGGAAGTTACCGGTGATTTTCTGGGTGTTGCA<br>CGTTGGGCAACCAGCCTGGTGGAAAGCTGTGAACTGATTGAAGGTGAAGCACATAAACC<br>GGGTTGTGTTCGTCGTGTTCTGGTTTATCCGCAGGCACCGGGTGAAGCAAGCACCTTTG<br>ATCTGAGCAAACTGCTGGAAATGGATGCACTGCATCATCGTTATAGTTATACCATTCTG<br>GGTGGTAGCACCCTGCCTGGTTTTAGCCTGATGCAGGATTATGTTAGCACCTTTAAACT<br>GAGCAGCCTGCGTCTGGTGTATCCGAGCGCAGAAATTGATCAAGAAAATGGCACCCTGC<br>TGCATTGGAGCTTTGTTTGTCGTCCGGTGAGCACCCTGAGCGAAGAAGAAACCCATAAT<br>ATTGCATTTAGCCTGTATCAGGCAGCCG

Supplementary Table 15. Isotig List

Entry	Sequence
1	>DtIsotig06604<br>TTTTAAACTTGTGTAGCAATAAATATACAAATATAAGTGATACACATCAACGAGTTTCGGTAGTAATTTATGTACA<br>AACTGCACATAAATTAATTATTGCACAGATAACAAAATGGGGAGAACCCCTACAGTATGCTGCAAAATTTGCAATA<br>TACATCACATCGAGGGTACCCACCATCATCTAAAAGCTTGCAGTTACTTGACTGAAGAGAATCTTGAAATCTGTCG<br>TCGGCGTACCTTCACCCTCCGGTTTCGAGGTCAAGTCAAAAGCCTTATTCTTTACTGCATCATGAACAATTGCCTG<br>TACACAGACTTCCGCAACATCTGCTCGTGGAACCGTTTTGGTATCTGTGGCGAGAAGTTCGTCGTTGTTTCCAATC<br>AAGAGCTCTCGCAACCCACCTTCTTTGTCTAGCAGCCCACCCGGCCTGATTAAGGTGTAGGTTGTATCAGAGTCTG<br>CCAAATACTGCTCCGACTTCCTTTTCCAAATCAAAATCTTCCCATTTCCGAAAGAGTTCAGAGGATGATTCGGATT<br>TGTGCCACCCATCGAGCCAACCAAAATGATATGCTTCACCCCCGCTTCTTTGGCTGCATCGATTTGATTCTTTTGC<br>CCGATCCAATCAACCTGCTCAGGATATGCGCCTTCTTCGTAGTAGAACTCGGGGCGCCCTCCTTTGCTTGGATCAA<br>ACCCTGGCTTCATTTGAGGAGCAGCACTTGTGAGGATGATGAGCACATCCACCCCTGCAAATGCTGGGCCCAGGCT<br>CTCTGCCTTTGTTATGTCACCTATGTACACATCCTCACCCCCTCCAATCTTTGCCTTGCTTGGCTCTGACCTCACA<br>AGACCTCTCACATGAAACTTGTCTGCCTTATGTTTCAGCTTCTCATATACAATGTGCCCCGTTCTTCCACCAGCAC<br>CAGTTACGAGTACGGTTGTGCGAGCAGAGTCTGCCATTGAAGCTGCAAGAAATGAAGATGGCGCAAAATGCTGAGA<br>CGAGGAGATGGCGCGACAAACCCTATATAAGCGTAATCTCAAGGTCTTCCAGAAGCTGCCATTTGTCGGCGATTTT<br>TGGGCGGTGGGCCTTTCCTAATTCTGTGTGTTCA
2	>DtIsotig07200<br>ATCTGCCTCCATTCAGAGCTATTCTTAGCCTGTGCTCCCCACGCTCCCTCCCTCCTCGGATTATCACCCCTCACTT<br>ACGCAGTATTATTTGGCGTGTTTTTTCATATTTGATATCGAGAGCAGCCATGAGTTATTGGAAGAGCAAGGTTGTG<br>CCCAAAATCAAGAAGTTTTTTGACAAGGGGAAGAAGAAAGGAGCTGCTGAGTTCTCCAAAAACTTTGATTCGTCCA<br>AGGAGTCTTTGGACAAGGAGATAGGAGAGAAGAGTTCAGATCTGAGCCCCAAGGTTGTGGAGATATACAGATCCTC<br>TTCCACCTTCATTGCCAAGAAGTTGCTGAAGGAACCCAATGAGGCAACAGTGAAGGAAAATTCCGATGCAACGCAA<br>GGCGTGCTTCAGGAGCTGGCAACAGCAGGCTTTCCTGGAGCGCAGGGCATCGCTGATGCAGGCAAAAAGTATGGAC<br>CGGCGCTTTTACCGGGGCCGGTCGTGTACTTGTTCGAGAAAGCATCCGTGTTTTTGGCGGAGGAGCCTTTGCCAGA<br>GGAGCCCAAGGCAGAGACTAGAGAGGTGAGTGCGGAGGACGTCAAGCCAGCAGAAGCGCCAGCGACGACTTCGGAA<br>ACTCCGCCGCCGCCACCTGTAGCAGACGTGCCTCCCCCAGCTGTCGTCGAGGAAGAAAAGAAGGAAGCAGAGCCCA<br>TTGTTGCTGCCCCGCCGCCCGAAGCTGCCCCCCCTGCAGCTGTTGAGATCCCCACCTCCGTTGACCCTACGCCCCC<br>GCCTCCTGCTCCGCCCGCCGACAAACCTGAGTAGTTTTCGCCTTCGATGCATAGTCGCACACCCATCCACACATCC<br>ATTGTCTTCTGTGCATTCATAAATTTTAATTACAATGTCCTTGTTCATAGTTTAATTACAATTTCTTTATTTTCAT<br>ATATAATCA
3	>DtIsotig04065<br>GTAAAAAGTTATGGTTTTTTCCATTGATACTTATCTAGAGACGTGTAAGAAGAAAAAAATCGCTTTAAACGGGTAA<br>TTTAAGAGTATCTTTTATTCAATGCATAACAACAATAGCTGCCTGACTAGCCAGGCAGCTGGGAAATTCATTTGGG<br>AGAGAAGCACAAACACACTGAATTGTAGCAGCTAGCTAGTATACAAAGTCAATGGCATAAAAGAACCATGAGACCG<br>TTTCCATGTTTACAGTAGCGGTTGAGACAAGGGACTGTTGAGCCACTCCGCGCACATACGCAAAGTCGCCCGTGCC<br>GCCAACAATGGGTGCAAACTTTACCGCATCTGACGCGACGTCCACCCCCAACAGGCTGAATGTGCCATTGTAGGTG<br>CCGTCGTTCCAGGTAAAGGTCTGCGCCAGGAACAACGTCAAAAGATTCTGCCCCACATCACCATACCAGCCCTGCA<br>CGTGCCCGAGCTGCGTAGAGTTGAGATCTGCGGCACTGGTGAGTGGGTTGTCGAAGGTGTGGATGATGCCGAATGA<br>GTTGGGTTGCGTCGAAAGCGGCTGCGCGGACTGCACGGCTGTGAAGGTGACATTGGGGTTGTCGAGATTGCTGTTA<br>TTCTGAACAGCAATGTACATGTAGAAGTCAAGATGGTCAGGACCCGAGGCAGCCAAAGCTGGCTGTGGGGCAGAGA<br>GGGAGGAGAGGGAGAGAGCGCATAATGTACATAGGAGCATAATGTGCACCTTGGCCATGAGATCGACAGAGGAGGG<br>GGAGGCTGAGAGTGAGCTTGTAGGGAGACGCGCGGACAATACGTCAAGTAGGAGTGTATGTAAGCTGTCGACAAAA<br>GCTGGGCTTGTGTGTGTATGAGAGGTAGAGAAGAAGAGAAGACTGTTATAGGTGATGAATGAGTTCTTCCATTTAT<br>AACGTTCTGGCCCTTCTGAGTGGATCAGACGTGCTTTGACAAGTCGCTCTACTGTGAGTGCCGGTGTGTGTGTGTG<br>CGCCGGATCGGAGGTCAAAGTGAGATTCCGGAAGCCTGATGTATTTCATAGAGAAAGCGATAAACGTTACAGACTG<br>AA
4	>DtIsotig04379<br>CTCTGGACCTTGGCTGTCACTCAAATTTTtGTTTAGAGGAGGAGGAAATGGGCACCTCAACGTGGGTGGTATGGAG<br>CATTCTGTTGCTAGCAGTGGCGCAAGTAGCGGGGAGCATCCCCATCCCAAGACGCTACGATGGCTTCGTCTTCAAT<br>GCTTCTTCATCCTCGTCGCCTGTGTTGCTGGAGGCCTTCTTCGATCCCCTCTGCCCGGATAGCGCAGACGCTTGGC<br>CTGTTGTCAAGAAAATCGCCCAATACTTCCAGGACGATCTGCTCCTCATTGTCCACCCCTTCCCTCTCCCGTACCA<br>TCACAATGCATATTTTGCAAGTAGAGCATTGCACATCATCAATAACCTGAACAGTTCTCTCACTTATCCATTGCTT<br>GAGTTGTTTTTTGAAAACCAGGATAGCTTTTCAACGAGTGAAACGCTAGCGGAGGCACCATCCTCCGTCGTAGACA<br>GAATCG5TTCAACTGGCAGCAGATAGCTTGAATGAACTCGTGTCTTCCGATTTTGAGAGCCAGTTCAAAGCAG6GA<br>TTTTCTGATACAGGAACGGATCTGATCACTCGTGTTTCGTTCAAGTTTGGGTGCTCGCGCGTTGTGGTCGGTACGC<br>CGTACTTTTTTGTCAATGGCATACCTCTTTATGATGCGGATTCGGCATGGACCTTCTCTGAATGGGCAGAGATTAT<br>TGAGCCATTAACAGGGGCGCAAAAAATCGCGCTTGCATAACGACCTCGGCAACACCAAAGCTGTATATTTGATCAT<br>TTAAGGgTGCtaCATtGCATGGAAGgTGCAGATACACTTTGCTTTtCTACTTGATTTtCctATCTCACAGTCTAAT<br>TTTAtACCaTAT

Entry	Sequence
5	>DtContig00505<br>CTCTGGACCTTGGCTGTCACTCAAATTTTTGTTTAGAGGAGGAGGAAATGGGCACCTCAACGTGGGTGGTATGGAGCA<br>TTCTGTTGCTAGCAGTGGCGCAAGTAGCGGGGAGCATCCCCATCCCAAGACGCTACGATGGCTTCGTCTTCAATGCTT<br>CTTCATCCTCGTCGCCTGTGTTGCTGGAGGCCTTCTTCGATCCCCTCTGCCCGGATAGCGCAGACGCTTGGCCTGTTG<br>TCAAGAAAATCGCCCAATACTTCCAGGACGATCTGCTCCTCATTGTCCACCCCTTCCCTCTCCCGTACCATCACAATG<br>CATATTTTGCAAGTAGAGCATTGCACATCATCAATAACCTGAACAGTTCTCTCACTTATCCATTGCTTGAGTTGTTTT<br>TTGAAAACCAGGATAGCTTTTCAACGAGTGAAACGCTAGCGGAGGCACCATCCTCCGTCGTAGACAGAATCG5TTCAA<br>CTGGCAGCAGATAGCTTGAATGAACTCGTGTCTTCCGATTTTGAGAGCCAGTTCAAAGCAG6GATTTTCTGATACAGG<br>AACGGATCTGATCACTCGTGTTTCGTTCAAGTTTGGGTGCTCGCGCGTTGTGGTCGGTACGCCGTACTTTTTTGTCAA<br>TGGCATACCTCTTTATGATGCGGATTCGGCATGGACCTTCTCTGAATGGGCAGAGATTATTGAGCCATTAACAGGGGC<br>GCAAAAAATCGCGCTTGCATAACGACCTCGGCAACACCAAAGCTGTATATTTGATCATTTAAGGGTGCTACATTGCAT<br>GGAAGGTGCAGATACACTTTGCTTTTCTACTTGATTTTCCTATCTCACAGTCTAATTTTATACCATAT
6	>DtIsotig02643<br>CTCTGGACCTTGGCTGTCACTCAAAAGAGAAGGTAAGGAGAGGAAAGCCATTAGGGGTTTGAGGAGAGTGAGGCGAGG<br>TAGGGATTATTAAGGGGGCAGCTCCCTAGCAAGTCATGGCGGGAACGGGAGGGGGCGCAGAACAGTTCCAGCTCCGGG<br>GAGTGCTGTGGGGGAAAGCCTACTCTTGGAAGATAACCGGAACGACAATCGACAAGGTGTGGTCGATTGTGGGCGATT<br>ATGTGCGCGTCGACAACTGGGTCTCTTCCGTCGTGAAGAGCTCGCACGTCGTGTCTGGCGAGGCCAACCAGACGGGGT<br>GCGTGAGGAGGTTCGTCTGCTACCCAGCCTCCGAGGGAGAGTCGGAGACTGTGGACTACTCGGAGCTCATCCACATGA<br>ACGCTGCCGCGCACCAGTACATGTACATGATCGTGGGAGGTAACATCACTGGCTTCTCTCTCATGAAGAACTATGTGA<br>GCAATATCTCGCTGTCTTCTCTTCCTGAGGAGGACGGTGGTGGTGTAATCTTTTACTGGAGCTTCACAGCCGAGCCTG<br>CCTCTAACCTCACGGAACAAAAATGCATAGAAATTGTGTTTCCTCTCTACACCACTGCCCTGAAGGATTTATGCACTC<br>ACCTTTCCATACCCGAAAGCTCTGTTACACTTCTCGATGATTAAGCTTTTCATCCTCCCCCCCCCAAAAAAAAAACAA<br>AACAAAACAAAACAAACTTTCTGCGTTCCCTAATACTTGTGCTGCGTTTCCTATCTTTTCCTTAATGCTCTACATCTT<br>GCGCTTCCTTACCCTAATAATTGCCGCTTTACACTTTCTAATAGATTTTCTGCTTTCTTGACTTGCCAAGGACTAAAT<br>ATAT
7	>DtIsotig02641<br>GTCTCTGGACCTTGGCTGTCACTCAAAAAAGAGAGGCGGAGGGAAGCCATTAGGAGTTGTGGGTTGAGAGAGTGAGGC<br>GAGGTAGGGATTATTAAGGGGGCAAGCCATGGCGGGAACGAGAGGAGGCGCTGAAGAGTTCCAGCTCCGGGGAGTGCT<br>GTGGGGGAAAGCCTACTCTTGGAAGATAACGGGAACGACAATCGACAAGGTGTGGTCGATTGTGGGTGATTATGTGCG<br>CGTCGACAACTGGGTCTCTTCCGTCGTGAAGAGCTCGCACGTCGTGTCCGGCGATGCCAACCAGACGGGGTGCGTGAG<br>GAGGTTCGTCTGCTACCCAGCCTCCGATGGAGAGTCGGAGACTGTGGACTACTCGGAGCTCATCCACATGAACGCCGC<br>CGCTCACCAATACATGTACATGATTGTGGGAGGTAACATCACTGGCTTCTCTCTCATGAAGAACTATGTGAGCAATAT<br>CTCGCTGTCTTCTCTTCCTGAGGAGGACGGTGGTGTGTCAATCTTTTACTGGAGCTTCACAGCCGAGCCTGCCTCTAA<br>CCTCACGGAACAAAAATGCATAGAAATTGTGTTTCCTCTCTACACCACTGCCCTGAAGGATTTATGCACTCACCTTTC<br>CATACCCGAAAGCTCTGTTACACTTCTCGATGATTAAGCTTTTCATCCTCCCCCCCCCAAAAAAAAAACAAAACAAAA<br>CAAAACAAACTTTCTGCGTTCCCTAATACTTGTGCTGCGTTTCCTATCTTTTCCTTAATGCTCTACATCTTGCGCTTC<br>CTTACCCTAATAATTGCCGCTTTACACTTTCTAATAGATTTTCTGCTTTCTTGACTTGCCAAGGACTAAATATAT
8	>DtIsotig07602<br>AGAATTAATGGTAGTAGGAACGAGTTGGTCCTGCCAACTATGTGCATATATTTAATAAAACCTTGGCAAACAAAACAT<br>TACTAACGGTATAGCAGCAATTATTAGGTGAGGTAGCTGCCGAGTTGGCTAAGGTAGAAAATGTAATTAGGGGGGTGA<br>GATAAAGCTTAATCACCGAGGAGTGTAACAGAGCTTTCCGGAATAGAAAGGTGAGTGCATAAATCCTTCAAGGCAGTG<br>GTATAGAGAGGGAACACAATTTCTATACATTTTTGTTCGGTGAGGTTAGAAGCAGGCTCGGCTGTGAAGCTCCAGTGG<br>AGGATGACACCACCACCGTCCGCCTCAGGAAGAGAATTGAGCGATATATTGCTCACATAGTTCTTCATGAGAGAGAAG<br>CCAGTGATGTTACCTCCCACAATCATGTACATGTACTGGTGCGCGGCCGCGTTCATGTGGATGAGCTCCGAGTAGTCC<br>ACAGTCTCCGACTCTCCCTCGGAGGCTGGGTAGCAGACGAACCTCCTCACGCACCCCGTCTTGTTAGCGTCGCCAGAC<br>ACGACGTGCGAGCTCTTCACTACAGAAGAGACCCAGTTGTCGACGCGCACATAGTCGCCCACAATCGCCCACACCTTG<br>TCAATTGTCGTTCCCGATATCTTCCACGAGTAGGCTTTCCCCCACAGCACTCCCCGCAGCTGGAACTCTTCTGCGCCC<br>CCTCCCGTTCCTGCCATGGCTTGCAAGCTTGCCTACTGCTCCTTACTCTCTCTCACTCTCTACCCTCAGCTCCCTCCT<br>CCGACTCTACTTTCCTTCTCTACTTCCCCAGGCTGATTGCAGCTGCTTTATAAAGTTT
9	>DtContig00751<br>ATTATTTAGCGGTGTAGCAGCAATTATTAGCTGAGGACATTAAGGGGTAGCTGCCGAGTTGGCTAAGGTAGAAAATAT<br>AATTAGGGAGGGGTGAGATAAAGCTTAATCACCGAGGAGTGTAACAGAGCTTTCCGGGATAGAAAGGTGAGTGCATAA<br>ATCCTTCAAGGCAGTGGTATAGAGAGGGAACACAATTTCGATGCATTTTTGTTCGGTGAGGTTAGAAGCAGGCTCGGC<br>TGTGAAGCTCCAGTGGAAGATGACACCACCTCCGTCCGCCTCAGGAAGAGAATTGAGCGATATATTGCTCACATAGTT<br>CTTCATGAGAGAGAAGCCAGTGATGTTACCTCCCACGATCATGTACATGTACTGGTGCGCGGCCGCATTCATGTGGAT<br>GAGCTCCGAGTAGTCCACAGTCTCCGACTCTCCCTCGGAGGCTGGGTAGCAGACGAACCTCCTCACGCACCCCGTCTT<br>GTTCGCATCGCCAGACACGACGTGCGAGCTCTTCACTACGGAAGAGACCCAGTTATCGACGCGGACATAGTCGCCCAC<br>AATCGACCACACCTTGTCGATTGTCGTTCCCGTTATCTTCCAAGAGTAGGCTTTCCCCCACAGCACTCCCCGCAGCTG<br>GAACTCTTCTGCGCCCCCTCCCGTTCCTGCCATGGCTTGGAAGCTTGCCTACTGCCCCTTACTCTCTCTCACTCTCTC<br>AATGCGCAGATCCCTCTTCCGGCTCTACTTACCTTATCTTTTGAGTGACAGCCAAGGTCCAGAGACGAGT

Entry	Sequence
**10**	>PtaIsotig02775<br>CTCTGGACCTTGGCTGTCACTCAAAGTGTGTGGCATTCACCGTAAGGAGGAGAGTTAAGGTGAGGCATCTGAGGTGCG<br>GTTGGGGCAACTTGGAGGACAGCGAGCTGTGTAAGGGAAGGATATTACAGCACAGGTGATGGAGACGATTCAAACAGC<br>GACGGAGTCGATGACAGCGGCTAGCAGGAGCTATGGAGAGGAGGAGGTATTATGGGGGAAGGCGTTCAAGTGGGAGAT<br>AAAGGGTGTAGGGGAGGACGAGGTGTGGGAGGTAACCGGAGACTTTCTGGGAGTGGCCAGGTGGGCAACCTCGCTGGT<br>GGAGAGCTGTGAGCTTATAGAAGGAGAGGCCCATAAGCCAGGCTGCGTGAGAAGGGTCCTTGTTTATCCCCAGGCTCC<br>TGGGGAGGCCTCCACTTTTGCCCTTGAAAAGCTCTTAGAAATGGACGCGCTACACCACCGTTACTCTTACACTATCCT<br>TGGCGGAAGCACCTTGCCTGGCTTCTCTCTCATGCAGGACTATGTCAGTACCTTCAAGCTCTCTTCCCTACGCCTGGT<br>GTACCCCTCTGCAGAAATTGACCAAGAAAATGGTACCCTCCTCCATTGGAGCTTTGTTTGTCGCCCAGTCTCTACCTT<br>GTCTGAGGAGGAAACCCACAACATTGCCTTCTCTCTCTACCAGGCTGCAGTCAACGATCTCAAAGCTCGCCTCTCCTT<br>GTCTGACGACCGCATTACTCTCATCCCGTAAACGTCTTTACCTAGCTAGGTGTGCGGTTGCAGGTAGTGGATTGCAAG<br>CCCTAAATGCCGATAGTCCCCCGCCTCCCAACCGTGCGCAGTGCATACCTAGGTATGCAGGGTCTCCTCAACAAAAGC<br>CCATGATGTTGTAGGCCACAAAGTGGAGATTACATTGGTAATTTGCAGGGTGAACTATGTATAAATGCTCTTCCTGGT<br>CATCTTAAAAGGTACCATAGTTGAAGGAGTTGCAAAGGGAGTGGTTCCTCACAGTAAAGTAAAAGTGTTATGTGAGTA<br>AAAGTGTATCAGATAACGTAAGCCTTGTTGTTAAGGTGGTTGCTTAGT
**11**	>PtaIsotig02778<br>CTCTGGACCTTGGCTGTCACTCAAAAAGGTGCGGTTGGGGCAACTTGGGGGGCAGCGAGCTGTGTAAGGGAAGGATAT<br>TTCAGCACAGGTGATGGAGACGATTCAAACAGCGGCTAGCAGGAGCTATGGAGAGGAGGAGGTATTATGGGGGAAGGC<br>GTTCAAGTGGGAGATAAAGGGTGCAGGGGAGGACGAGGTGTGGGAGGTAACCGGAGACTTTCTGGGAGTGGCCAGGTG<br>GGCAACCTCGCTGGTGGAGAGCTGTGAGCTTATAGAAGGAGAGGCCCATAAGCCAGGCTGCGTGAGAAGGGTCCTTGT<br>TTATCCCCAGGCTCCTGGGGAGGCCTCCACTTTTGCCCTTGAAAAGCTCTTAGAAATGGACGCGCTACACCACCGTTA<br>CTCTTACACTATCCTTGGCGGAAGCACCTTGCCTGGCTTCTCTCTCATGCAGGACTATGTCAGTACCTTCAAGCTCTC<br>TTCCCTACGCCTGGTGTACCCCTCTGCAGAAATTGACCAAGAAAATGGTACCCTCCTCCATTGGAGCTTTGTTTGTCG<br>CCCAGTCTCTACCTTGTCTGAGGAGGAAACCCACAACATTGCCTTCTCTCTCTACCAGGCTGCAGTCAACGATCTCAA<br>AGCTCGCCTCTCCTTGTCTGACGACCGCATTACTCTCATCCCGTAAACGTCTTTACCTAGCTAGGTGTGCGGTTGCAG<br>GTAGTGGATTGCAAGCCCTAAATGCCGATAGTCCCCCGCCTCCCAACCGTGCGCAGTGCATACCTAGGTATGCAGGGT<br>CTCCTCAACAAAAGCCCATGATGTTGTAGGCCACAAAGTGGAGATTACATTGGTAATTTGCAGGGTGAACTATGTATA<br>AATGCTCTTCCTGGTCATCTTAAAAGGTACCATAGTTGAAGGAGTTGCAAAGGGAGTGGTTCCTCACAGTAAAGTAAA<br>AGTGTTATGTGAGTAAAAGTGTATCAGATAACGTAAGCCTTGTTGTTAAGGTGGTTGCTTAGT
**12**	>PtaContig4149<br>AGACTCCTTCAACTATGGTACCTTTAAAGATGACCAGGAAGAGCATTTATACATAGTTCACCCTGCAAATTACCAATG<br>TAATCTCCACTTTGTGGCCTACAACATCATGGGCTTTTGTTGGGGAGACCCTGCATACCTAGGTATGCATTGCGCACT<br>GTTGGGAGGCGGGGGACTATCGCCATTTAGGGCTTGCAATCCACTACCTGCAACCACACACCTAGCTAGGTAAAGATG<br>CTTACGAGATGAGAGTAATGCGGTCGTCAGACAAGGAGAGGCGAGCTTTGAGATCGTTGACTGCAGCCTGGTAGAGAG<br>AGAAGGCAATGTTGTGGGTTTCCTCTTCAGACAAGGTAGAGACTGGGCGACAAACGAAGCTCCAATGGAGGAGGGTAC<br>CATTTTCTTGGTCAATTTCTGCAGAGGGGTACACCAGGCGTAGGGAAGAGAGCTTGAAGGTACTGACATAATCCTGCA<br>TGAGAGAGAAGCCAGGCAAGGTGCTTCCGCCAAGGATAGTGTAAGAGTAATGGTGGTGTAGGGCGTCCATTTCTAAGA<br>GCTTTTCAAGGGCAAAAGTGGAGGCTTCCCCAGGAGCCTGGGGATAAACAAGGACCCTTCTCACGCAGCCTGGCTTAT<br>GGGCCTCTCCTTCTATAAGCTCACAGCTCTCCACCAGCGAGGTTGCCCACCTGGCCACTCCCAGAAAGTCTCCGGTTA<br>CCTCCCACACCTCGTCCTCCCCTACACCCTTTATCTCCCACTTGAACGCCTTCCCCCATAATACCTCCTCCTCTCCAT<br>AGCTCCTGCTAGCCGCTGTCATCGACTCCGTCGCTGTTTGAATCGTCTCCATCACCTGTGCTGTAATATCCTTCCCTT<br>ACACAGCTCGCTGTCCTCCAAGTTGCCCCAACCGCACCTCAGATGCCTCACCTGTGTTTACCTTAACTCTCCTCCTTA<br>CGGTGAATGCCACACCCCCGCGT
**13**	>DtIsotig4300<br>CAAGTATTCCTAAGTAATACAAGGTACACAATCCTCAATCTAGAAGAGTGATGGAATCGTCTGGTAAAGCAAGGTGCG<br>TTTTCAAATCATTGATGGCTGTTTCGTAGAGCGAAAATGCAATTTTATGGGTGTCTTGTTCGGAGAGGGTGGAGACAG<br>GATGGCAAGTGAAGCTCCACTGAAGAAGGGTAGCATCATCGTCCTTAGAGGTATCAAGGATGTCAATATTGGTGTTTG<br>TAGTATGAAGACTAGAGGGCACGTGATGGCCATTGGACTTACTTGTTGTAGTAAGAGAGGTAAGCTTAAACGAGCTAA<br>CATAGCCTTGCATAAGAGAGAAACCAGGTAAAGTACCTCCTATAATGGCATATTGAAAGTGGTGAGAGGCGGGGTCCA<br>TGAGAACAAGCTTCTCAAGGCCGAAGGTGGAGGATTGCCCCGGTGAGGCTGGGTAGATGACGGCCCGCCTCACACAGC<br>CCGGCTTCTGAGGCTCTCCTTGGATGAGCTCGCAGCTCTGCACGAGCATGGTGGCCCACTTGTCGACGCATAGGAAGT<br>CGCTCGTGATCGCCCACACCTTCTCTACCCCGGCTCCTCTGATCTTCCACGTGAAGGATTTCCCCCACAGCCCTCCCT<br>TCTGTGGTTCTTCTTCCATGGCGTGCCCTTAGCTGCTGGCTTGCAGAAGTTGCAGCTGCGCTTCTGTTGCCTCACGAC<br>TGCCTCTGCTCTCCCCTTCTGTATATTTACACGTCTTTAAAGGCAAAGGCTCCAGCGCTCGCGCACGCACACACACTC<br>ACTCGCCAATACGACACGTGTATGTGCTCTATTCGCTACCTTATCTACTGCTGTTTTGACATATCGGATGGGCTACGA<br>ATTCTG

References

1. Krammer, B., Rumbold, K., Tschemmernegg, M., Pöchlauer, P. & Schwab, H. A novel screening assay for hydroxynitrile lyases suitable for high-throughput screening. *J. Biotechnol.* **129**, 151–61 (2007).
2. Wajant, H., Forster, S., Selmar, D., Effenberger, F. & Pfizenmaier, K. Purification and Characterization of a Novel (*R*)-Mandelonitrile Lyase from the Fern *Phlebodium aureum*. *Plant Physiol.* **109**, 1231–1238 (1995).
3. Sievers, F. *et al.* Fast, scalable generation of high-quality protein multiple sequence alignments using Clustal Omega. *Mol. Syst. Biol.* **7**, 539 (2011).
4. Der, J. P., Barker, M. S., Wickett, N. J., dePamphilis, C. W. & Wolf, P. G. *De novo* characterization of the gametophyte transcriptome in bracken fern, *Pteridium aquilinum*. *BMC Genomics* **12**, 99 (2011).
5. Lanfranchi, E. *et al.* Bioprospecting for Hydroxynitrile Lyases by Blue Native PAGE Coupled HCN Detection. 111–117 (2015).
6. Radauer, C., Lackner, P. & Breiteneder, H. The Bet v 1 fold: an ancient, versatile scaffold for binding of large, hydrophobic ligands. *BMC Evol. Biol.* **8**, 286 (2008).
7. Finn, R. D. et al. Pfam: The protein families database. *Nucleic Acids Res.* **42**, 222–230 (2014).
8. Imai, S. *et al.* Plant biochemistry: an onion enzyme that makes the eyes water. *Nature* **419**, 685 (2002).
9. Yin, P. *et al.* Structural insights into the mechanism of abscisic acid signaling by PYL proteins. *Nat. Struct. Mol. Biol.* **16**, 1230–1236 (2009).
10. Lim, C. W. & Lee, S. C. Arabidopsis abscisic acid receptors play an important role in disease resistance. *Plant Mol. Biol.* **88**, 313–324 (2015).
11. Kuipers, R. K. *et al.* 3DM: systematic analysis of heterogeneous superfamily data to discover protein functionalities. *Proteins* **78**, 2101–13 (2010).
12. Consortium, T. U. UniProt: a hub for protein information. *Nucleic Acids Res.* **43**, D204–D212 (2014).
13. Masamura, N. *et al.* Identification of Amino Acid Residues Essential for Onion Lachrymatory Factor Synthase Activity. *Biosci. Biotechnol. Biochem.* **76**, 447–453 (2012).

14. Kelley, L. A., Mezulis, S., Yates, C. M., Wass, M. N. & Sternberg, M. J. E. The Phyre2 web portal for protein modeling, prediction and analysis. *Nat. Protoc.* 10, 845–858 (2015).
